# Supplementary material for: ERAP1 polymorphisms interactions and their association with Behçet’s disease susceptibly: Application of Model-Based Multifactor Dimension Reduction Algorithm (MB-MDR)
Source: PLoS One. 2020 Feb 5;15(2):e0227997. doi: 10.1371/journal.pone.0227997 (PMC7001967; doi:10.1371/journal.pone.0227997)
Supplement: S1 Table — (DOCX) [file pone.0227997.s001.docx]

**Table.** Model-based multifactor dimensionality reduction algorithm for assessing the main and interaction effects of 11 ERAP1 SNPs on Behçet’s disease risk (748 Iranian BD patients and 776 healthy individuals)

| Order | Significant Effects | Synergistic Effect | | | | | Antagonism Effect | | | | | Permutation Test |
| --- | --- | --- | --- | --- | --- | --- | --- | --- | --- | --- | --- | --- |
|  |  | **N. levels** | **Genotypes** | **Coefficient** | | **Adj. P-value** | **N. levels** | **Genotypes** | **Coefficient** | | **Adj. P-value** | **Perm. P-value** |
| Main Effects | rs1065407 | 1 | TT | 0.23 | | 0.034 | 0 | NA | NA | | NA | 0.019 |
|  | rs30187 | 0 | NA | NA | | NA | 1 | TT | -0.26 | | 0.041 | 0.18 |
|  | rs469876 | 0 | NA | NA | | NA | 1 | AA | -0.20 | | 0.046 | 0.054 |
| # 2 Interaction Effects studies | rs27434+rs26653 | 1 | GG+CC | 12.79 | | 1.77×${10}^{-10}$ | 1 | AA+CG | -0.31 | | 7.24×${10}^{-2}$ | 0.009 |
|  | rs17481856+rs26653 | 1 | AA+CG | 12.79 | | 1.79×${10}^{-10}$ | 0 | NA | NA | | NA | 0.009 |
|  | rs27434+rs30187 | 1 | GG+TT | 12.78 | | 1.81×${10}^{-10}$ | 0 | NA | NA | | NA | 0.011 |
|  | rs17482078+rs30187 | 1 | TT+CT | 12.78 | | 1.81×${10}^{-10}$ | 0 | NA | NA | | NA | 0.014 |
|  | rs28096+rs17481856 | 1 | AA+AG | 12.78 | | 1.83×${10}^{-10}$ | 0 | NA | NA | | NA | 0.019 |
|  | rs13167972+rs27044 | 1 | AG+GG | 12.77 | | 1.88×${10}^{-10}$ | 1 | AA+CG | -0.40 | | 8.92×${10}^{-2}$ | 0.011 |
|  | rs27044+rs1065407 | 1 | GG+GT | 12.77 | | 1.90×${10}^{-10}$ | 1 | CG+TT | -0.44 | | 3.65×${10}^{-2}$ | 0.013 |
|  | rs27044+rs2287987 | 1 | CC+CC | 12.76 | | 1.98×${10}^{-10}$ | 1 | GG+CT | -0.42 | | 1.72×${10}^{-2}$ | 0.014 |
|  | rs27434+rs2287987 | 1 | GG+CC | 12.75 | | 2.01×${10}^{-10}$ | 0 | NA | NA | | NA | 0.017 |
|  | rs30187+rs2287987 | 1 | CC+CC | 12.75 | | 2.01×${10}^{-10}$ | 1 | TT+CT | -0.28 | | 9.92×${10}^{-2}$ | 0.017 |
|  | rs13167972+rs1065407 | 2 | AG+GG  AA+GG | 12.75 | | 2.01×${10}^{-10}$ | 0 | NA | NA | | NA | 0.018 |
|  | rs10050860+rs2287987 | 2 | CC+TT  CT+CT | 12.75 | | 2.05×${10}^{-10}$ | 0 | NA | NA | | NA | 0.058 |
|  | rs2287987+rs1065407 | 1 | CC+GG | 12.74 | | 2.12×${10}^{-10}$ | 0 | NA | NA | | NA | 0.065 |
|  | rs30187+rs1065407 | 0 | NA | NA | | NA | 1 | TT+CT | -0.39 | | 1.98×${10}^{-3}$ | 0.053 |
|  | rs469876+rs1065407 | 2 | GG+AA  GT+GG | 0.32 | | 4.73×${10}^{-3}$ | 0 | NA | NA | | NA | 0.181 |
|  | rs13167972+rs30187 | 2 | GG+CC  AA+TT | 0.68 | | 8.33×${10}^{-3}$ | 1 | AG+CT | -0.40 | | 9.55×${10}^{-2}$ | 0.126 |
|  | rs469876+rs27434 | 1 | AA+AA | 0.50 | | 1.02×${10}^{-2}$ | 1 | AG+AG | -0.38 | | 7.36×${10}^{-2}$ | 0.152 |
|  | rs28096+rs469876 | 1 | AA +GG | 0.62 | | 1.13×${10}^{-2}$ | 1 | AG+AG | -1.22 | | 6.65×${10}^{-2}$ | 0.302 |
|  | rs26653+rs27044 | 1 | CC+CC | 0.87 | | 1.18×${10}^{-2}$ | 1 | CG+CG | -0.39 | | 5.93×${10}^{-2}$ | 0.036 |
|  | rs469876+rs27044 | 2 | AA+ CC  AG +GG | 0.42 | | 2.03×${10}^{-2}$ | 0 | NA | NA | | NA | 0.028 |
|  | rs10050860+rs27044 | 1 | TT+GG | 1.13 | | 9.37×${10}^{-2}$ | 1 | CT+CG | -0.40 | | 2.16×${10}^{-2}$ | 0.059 |
|  | rs30187+rs469876 | 2 | CC+AA  TT+AG | 0.32 | | 2.39×${10}^{-2}$ | 0 | NA | NA | | NA | 0.091 |
|  | rs26653+rs1065407 | 1 | CC+GG | 0.76 | | 2.49×${10}^{-2}$ | 0 | NA | NA | | NA | 0.210 |
|  | rs26653+rs469876 | 2 | CC+AA  GG+AG | 0.54 | | 2.83×${10}^{-2}$ | 1 | CT+AG | -0.42 | | 7.38×${10}^{-2}$ | 0.193 |
|  | rs17482078+rs28096 | 3 | TT+AA  CT+AG | 1.42 | | 2.90×${10}^{-2}$ | 0 | NA | NA | | NA | 0.098 |
|  | rs17482078+rs27044 | 2 | TT+GG  CT+CG | 1.23 | | 6.35×${10}^{-2}$ | 1 | CT+CG | -0.38 | | 3.08×${10}^{-2}$ | 0.057 |
|  | rs26653+rs13167972 | 1 | CC+GG | 0.71 | | 3.57×${10}^{-2}$ | 0 | NA | NA | | NA | 0.177 |
|  | rs13167972+rs27434 | 1 | GG+AA | 0.59 | | 3.66×${10}^{-2}$ | 0 | NA | NA | | NA | 0.221 |
|  | rs1065407+rs27434 | 1 | AA+GG | 0.66 | | 4.09×${10}^{-2}$ | 0 | NA | NA | | NA | 0.312 |
|  | rs10050860+rs28096 | 2 | TT+AA  CT+AG | 1.33 | | 4.23×${10}^{-2}$ | 0 | NA | NA | | NA | 0.162 |
|  | rs1065407+rs28096 | 1 | GG+AA | 0.63 | | 4.57×${10}^{-2}$ | 0 | NA | NA | | NA | 0.549 |
| #3Interaction Effects studies | rs30187+rs10050860+rs27434 | 1 | TT+TT+ AA | 12.78 | | 1.82×${10}^{-10}$ | 1 | CT+CT+ AG | NA | | NA | 0.120 |
|  | rs30187+rs17481856+rs17482078 | 1 | TT+AA+CC | 12.78 | | 1.86×${10}^{-10}$ | 0 | NA | NA | | NA | 0.061 |
|  | rs27044+rs27434+rs17481856 | 2 | CC +AA+AA    GG +AG+AG | 12.78 | | 1.86×${10}^{-10}$ | 0 | NA | NA | | NA | 0.118 |
|  | rs30187+rs10050860+rs469876 | 2 | CC+TT+ AA  TT+CT+ AG | 12.77 | | 1.90×${10}^{-10}$ | 0 | NA | NA | | NA | 0.163 |
|  | rs1065407+rs27044+rs17481856 | 2 | GG+GG+ AA  GT+CG+ AG | 12.77 | | 1.90×${10}^{-10}$ | 0 | NA | NA | | NA | 0.139 |
|  | rs27044+rs17481856+rs13167972 | 3 | GG+AA+ GG  CG+AA+ AA | 12.77 | | 1.90×${10}^{-10}$ | 0 | NA | NA | | NA | 0.151 |
|  | rs2287987+rs27044+rs27434 | 1 | CC+CC +AA | 12.75 | | 2.01×${10}^{-10}$ | 0 | NA | NA | | NA | 0.184 |
|  | rs2287987+rs27044+rs17481856 | 1 | CC+CC+ AA | 12.75 | | 2.03×${10}^{-10}$ | 1 | TT+CG+ AG | -0.47 | | 9.36×${10}^{-2}$ | 0.118 |
|  | rs2287987+rs10050860+rs27044 | 3 | CC+NA+ GG | 12.75 | | 2.06×${10}^{-10}$ | 1 | CT+NA+ CG | -0.42 | | 1.72×${10}^{-2}$ | 0.057 |
|  | rs2287987+rs27434+rs17481856 | 1 | CC+AA+ AA | 12.75 | | 2.06×${10}^{-10}$ | 0 | NA | NA | | NA | 0.111 |
|  | rs2287987+rs30187+rs17481856 | 1 | CC+ TT+ AA | 12.75 | | 2.06×${10}^{-10}$ | 0 | NA | NA | | NA | 0.123 |
|  | rs2287987+rs30187+rs27434 | 2 | CC+CC+ AA  CT+TT+ AG | 12.75 | | 2.06×${10}^{-10}$ | 0 | NA | NA | | NA | 0.109 |
|  | rs2287987+rs10050860+rs27434 | 3 | CC+NA+AA  TT+NA +AG | 12.74 | | 2.08×${10}^{-10}$ | 0 | NA | NA | | NA | 0.058 |
|  | rs2287987 +rs10050860+rs30187 | 3 | CC+NA+ TT  TT+NA +TT | 12.74 | | 2.08×${10}^{-10}$ | 1 | CT+NA+ CT | -0.28 | | 9.92×${10}^{-2}$ | 0.044 |
|  | rs1065407+rs17481856+rs13167972 | 4 | GG+ AA +GG  GT+ AG +AA | 12.74 | | 2.08×${10}^{-10}$ | 1 | TT+ AG+ AG | -0.57 | | 6.49×${10}^{-2}$ | 0.134 |
|  | rs2287987+rs10050860+rs17481856 | 2 | CC+NA+AA  TT+NA+AG | 12.74 | | 2.10×${10}^{-10}$ | 1 | CT+NA+AG | -0.44 | | 7.18×${10}^{-2}$ | 0.088 |
|  | rs2287987+rs469876+rs17481856 | 1 | CC+AA+AA | 12.74 | | 2.10×${10}^{-10}$ | 1 | TT+ AG+ AG | -0.83 | | 7.17×${10}^{-2}$ | 0.131 |
|  | rs2287987+rs17481856+rs28096 | 2 | CC+ AA+ AA  CT+ AG+ AG | 12.74 | | 2.11×${10}^{-10}$ | 1 | TT+ AG+ AG | -0.61 | | 4.×${10}^{-2}$ | 0.132 |
|  | rs1065407+rs27044+rs13167972 | 5 | GG+GG +GG  GT+CG+  AA | 12.74 | | 2.11×${10}^{-10}$ | 1 | TT+CG+ AG | -0.44 | | 6.93×${10}^{-2}$ | 0.161 |
|  | rs1065407+rs30187+rs13167972 | 5 | GG+ TT +GG  GT+ CT+ AA | 12.74 | | 2.11×${10}^{-10}$ | 1 | TT+ CT +AG | -0.47 | | 5.56×${10}^{-2}$ | 0.169 |
|  | rs2287987+rs27044+rs469876 | 3 | CC+CC+ AA  TT+GG+ AG | 12.74 | | 2.13×${10}^{-10}$ | 0 | NA | NA | | NA | 0.034 |
|  | rs2287987+rs10050860+rs469876 | 3 | CC+ NA +AA  TT+ NA +AG | 12.74 | | 2.13×${10}^{-10}$ | 0 | NA | NA | | NA | 0.050 |
|  | rs1065407+rs2287987+rs17481856 | 1 | GG+ CC+ AA | 12.74 | | 2.13×${10}^{-10}$ | 1 | TT+ CT+AG | -0.59 | | 4.12×${10}^{-2}$ | 0.120 |
|  | rs1065407+rs2287987+rs26653 | 1 | GG+ CC+ CC | 12.74 | | 2.13×${10}^{-10}$ | 0 | NA | NA | | NA | 0.243 |
|  | rs1065407+rs2287987+rs27434 | 1 | GG+ CC+ AG | 12.73 | | 2.15×${10}^{-10}$ | 0 | NA | NA | | NA | 0.380 |
|  | rs1065407+rs2287987+rs30187 | 1 | GG+ CC+ TT | 12.73 | | 2.15×${10}^{-10}$ | 1 | TT+ CT+ CT | -0.39 | | 5.95×${10}^{-2}$ | 0.230 |
|  | rs1065407+rs2287987+rs10050860 | 3 | GG+ CC+ NA  TT+ TT+ NA | 12.73 | | 2.20×${10}^{-10}$ | 0 | NA | NA | | NA | 0.187 |
|  | rs1065407+rs30187+rs469876 | 3 | GG+ TT +AG  GT+ CT + AG | 0.43 | | 2.87×${10}^{-2}$ | 1 | TT+ CT+ AG | -0.67 | | 1.26×${10}^{-3}$ | 0.169 |
|  | rs1065407+rs27044+rs26653 | 6 | GG+CC+ GG  GT+CG+ GG | 0.99 | | 1.34×${10}^{-3}$ | 0 | NA | NA | | NA | 0.066 |
|  | rs27044+rs26653+rs469876 | 4 | CC+CC+ AA  GG+GG+ AG | 0.77 | | 1.35×${10}^{-3}$ | 1 | CG+CG+ AG | -0.45 | | 7.26×${10}^{-2}$ | 0.021 |
|  | rs27044+rs26653+rs17482078 | 3 | CC+CC+TT  GG+GG+CT | 0.98 | | 1.53×${10}^{-3}$ | 0 | NA | NA | | NA | 0.087 |
|  | rs1065407+rs26653+rs13167972 | 10 | GG+CC+GG  GT+GG+AA | 0.97 | | 1.79×${10}^{-3}$ | 0 | NA | NA | | NA | 0.083 |
|  | rs10050860+rs27044+rs26653 | 2 | TT+ CC+ CC  CT+GG+ GG | 0.95 | | 2.12×${10}^{-3}$ | 1 | CT+ CG+ CG | -0.37 | | 8.98×${10}^{-2}$ | 0.042 |
|  | rs26653+rs27434+rs469876 | 3 | CC+GG+ AA  CG+AG+ AG | 0.51 | | 3.73×${10}^{-2}$ | 2 | GG+AA+ AG  CG+AG+ AA | -0.52 | | 2.45×${10}^{-3}$ | 0.187 |
|  | rs27044+rs27434+rs13167972 | 8 | GG+AA+ GG  CG+ AG+AA | 0.81 | | 3.62×${10}^{-3}$ | 0 | NA | NA | | NA | 0.239 |
|  | rs27044+rs26653+rs28096 | 4 | CC+CC+ AA  GG+GG+ AG | 0.77 | | 3.88×${10}^{-3}$ | 0 | NA | NA | | NA | 0.058 |
|  | rs30187+rs27044+rs469876 | 5 | CC+CC+ AA  GG+TT+ AG | 0.60 | | 4.19×${10}^{-3}$ | 0 | NA | NA | | NA | 0.032 |
|  | rs30187+rs17481856+rs13167972 | 3 | CC+AA+ GG  TT +AG +AA | 0.70 | | 6.08×${10}^{-3}$ | 0 | NA | NA | | NA | 0.098 |
|  | rs1065407+rs27434+rs469876 | 2 | GG+GG+ AA  GT+AG+ AG | 0.58 | | 6.62×${10}^{-3}$ | 1 | TT+AG+ AG | -0.52 | | 6.59×${10}^{-2}$ | 0.199 |
|  | rs1065407+rs27434+rs13167972 | 5 | GG+AA+ GG  GT+AG+ AA | 0.85 | | 7.25×${10}^{-3}$ | 0 | NA | NA | | NA | 0.160 |
|  | rs27044+rs469876+rs28096 | 3 | CC+AA+ AA  GG+AG+ AG | 0.66 | | 7.81×${10}^{-3}$ | 2 | CG+AG+ AG  GG+AA+ AG | -1.10 | | 8.74×${10}^{-3}$ | 0.034 |
|  | rs30187+rs469876+rs13167972 | 3 | CC+AA+ GG  TT+AG+ AA | 0.78 | | 9.39×${10}^{-3}$ | 1 | CT+AG+AG | -0.50 | | 6.13×${10}^{-2}$ | 0.567 |
|  | rs27044+rs26653+rs13167972 | 4 | GG+CC+ AG  CG+GG+ AA | 0.87 | | 1.14×${10}^{-2}$ | 1 | CG+CG+ AA | -0.54 | | 7.96×${10}^{-2}$ | 0.072 |
|  | rs27044+rs27434+rs469876 | 2 | CC+AA +AA  GG+AG+ AG | 0.57 | | 1.23×${10}^{-2}$ | 0 | NA | NA | | NA | 0.045 |
|  | rs469876+rs17481856+rs28096 | 2 | AA+AA+ AA  AG+AG+ AG | 0.62 | | 1.24×${10}^{-2}$ | 1 | AG+AG+ AG | -1.22 | | 6.49×${10}^{-2}$ | 0.344 |
|  | rs27434+rs469876+rs17481856 | 2 | AA+AA+ GG  AG+AG+ AA | 0.56 | | 1.36×${10}^{-2}$ | 0 | NA | NA | | NA | 0.112 |
|  | rs27434+rs469876+rs28096 | 2 | AA+AA+ AA  AG+AG+ AG | 0.60 | | 1.45×${10}^{-2}$ | 1 | AG+AG+ AG | -1.11 | | 9.99×${10}^{-2}$ | 0.317 |
|  | rs30187+rs26653+rs13167972 | 3 | CC+CC+ GG | 0.84 | | 1.53×${10}^{-2}$ | 0 | NA | NA | | NA | 0.271 |
|  | rs1065407+rs27044+rs27434 | 5 | GG+GG+ AA  GT+GC+ AG | 0.77 | | 1.54×${10}^{-2}$ | 0 | NA | NA | | NA | 0.195 |
|  | rs469876+rs17481856+ rs30187 | 2 | GG+GG+ CT  TT+AG+ AG | 0.56 | | 1.61×${10}^{-2}$ | 0 | NA | NA | | NA | 0.127 |
|  | rs26653+rs28096+rs13167972 | 8 | CC+AA +GG  GG+AG+AA | 0.76 | | 1.64×${10}^{-2}$ | 0 | NA | NA | | NA | 0.069 |
|  | rs10050860+rs27434+rs469876 | 3 | TT+AA+ AA  CT+AG+ AG | 0.64 | | 1.74×${10}^{-2}$ | 1 | CT+AG+ AG | -0.71 | | 8.81×${10}^{-2}$ | 0.110 |
|  | rs1065407+rs26653+rs17481856 | 2 | GG+CC+ AA  GT+GG+ AG | 0.80 | | 1.78×${10}^{-2}$ | 0 | NA | NA | | NA | 0.108 |
|  | rs27044+rs469876+rs17481856 | 2 | CC+AA+ AA  AG+GG+ AG | 0.43 | | 1.81×${10}^{-2}$ | 0 | NA | NA | | NA | 0.052 |
|  | rs469876+rs28096+rs13167972 | 5 | GG+GG+ AA  AA+AG+ AA | 0.67 | | 1.85×${10}^{-2}$ | 1 | AG+AG+ AG | -0.48 | | 6.44×${10}^{-2}$ | 0.339 |
|  | rs1065407+rs469876+rs13167972 | 8 | GG+GG+ GG  AA+AA+ GT | 2.46 | | 1.90×${10}^{-2}$ | 1 | AG+AG+ TT | -0.49 | | 5.08×${10}^{-2}$ | 0.434 |
|  | rs27434+rs469876+rs17482078 | 4 | AA+AA+ TT  AG+AG+ CT | 0.62 | | 1.94×${10}^{-2}$ | 1 | AG+AG+ CT | -0.71 | | 8.66×${10}^{-2}$ | 0.125 |
|  | rs27434+ rs26653+rs13167972 | 3 | GG+CC+ GG  AG+CG+ AA | 0.76 | | 1.99×${10}^{-2}$ | 0 | NA | NA | | NA | 0.084 |
|  | rs17481856+rs28096+rs17482078 | 5 | AA+AA+ TT  AG+AG+CT | 1.50 | | 2.04×${10}^{-2}$ | 1 | AG+AG+CT | -0.48 | | 9.19×${10}^{-2}$ | 0.090 |
|  | rs30187+rs27044+rs13167972 | 8 | TT+CC+ GG  CT+GG+ AA | 0.64 | | 2.10×${10}^{-2}$ | 1 | CT+CG+ AG | -0.40 | | 9.19×${10}^{-2}$ | 0.277 |
|  | rs1065407+rs469876+rs28096 | 4 | GG+GG+ AA  GT+AA+ AG | 0.74 | | 2.14×${10}^{-2}$ | 1 | TT+AG+ AG | -1.95 | | 6.91×${10}^{-2}$ | 0.451 |
|  | rs2287987+rs27044+rs26653 | 2 | CC+CC+ CC  CT+GG+ GG | 0.78 | | 2.16×${10}^{-2}$ | 0 | NA | NA | | NA | 0.047 |
|  | rs10050860+rs26653+rs27434 | 3 | TT +CC+GG  CT+ CG+AG | 0.89 | | 2.19×${10}^{-2}$ | 0 | NA | NA | | NA | 0.091 |
|  | rs1065407+rs27044+rs28096 | 2 | GG+GG+ AA  GT+CG+ AG | 0.73 | | 2.21×${10}^{-2}$ | 1 | TT+CT+ AG | -0.66 | | 9.89×${10}^{-2}$ | 0.098 |
|  | rs28096+ rs30187+rs13167972 | 7 | AA+TT+ GG  AG+CT+ AA | 0.67 | | 2.25×${10}^{-2}$ | 0 | NA | NA | | NA | 0.218 |
|  | rs30187+ rs1065407+rs26653 | 4 | CC+GG+CC  TT+GT+GG | 0.77 | | 2.36×${10}^{-2}$ | 0 | NA | NA | | NA | 0.137 |
|  | rs27434+rs469876+rs13167972 | 1 | GG+GG+ AA | 0.52 | | 4.76×${10}^{-2}$ | 1 | AG+AG+AG | -0.61 | | 2.43×${10}^{-2}$ | 0.142 |
|  | rs27044+rs28096+rs17482078 | 6 | GG+AA+ TT  CG+AG+CT | 1.29 | | 2.43×${10}^{-2}$ | 1 | CG+AG+CT | -0.36 | | 9.36×${10}^{-2}$ | 0.070 |
|  | rs30187+rs469876+rs28096 | 4 | GG+CC+ AA  AA+TT+ AG | 0.56 | | 2.46×${10}^{-2}$ | 0 | NA | NA | | NA | 0.219 |
|  | rs2287987+rs27044+rs17482078 | 5 | TT+GG+ TT  CT+CG+CT | -1.37 | | 2.22×${10}^{-1}$ | 1 | CT+CG+CT | -0.40 | | 2.48×${10}^{-2}$ | 0.034 |
|  | rs10050860+rs26653+rs469876 | 3 | TT+CC+ GG  CT+GG+AA | 0.61 | | 2.78×${10}^{-1}$ | 1 | CT+CG+ AG | -0.46 | | 2.51×${10}^{-2}$ | 0.092 |
|  | rs26653+rs17481856+rs13167972 | 3 | CC+GG+AA  GG+AG+ AA | 0.75 | | 2.57×${10}^{-2}$ | 0 | NA | NA | | NA | 0.091 |
|  | rs30187+rs27434+rs469876 | 3 | CC+AA+ AA  TT+AG+ AG | 0.51 | | 2.64×${10}^{-2}$ | 0 | NA | NA | | NA | 0.402 |
|  | rs30187+rs26653+rs28096 | 5 | CC+CC+ AA  TT+TT+AG | 0.68 | | 2.68×${10}^{-2}$ | 0 | NA | NA | | NA | 0.155 |
|  | rs1065407+rs26653+rs27434 | 3 | CC+GG+ GG  CG+GT+ AG | 0.73 | | 2.70×${10}^{-2}$ | 0 | NA | NA | | NA | 0.066 |
|  | rs2287987+rs30187+rs13167972 | 5 | CC+ TT+GG  CT+CT+ AA | 0.69 | | 2.70×${10}^{-2}$ | 0 | NA | NA | | NA | 0.199 |
|  | rs1065407+rs26653+rs28096 | 3 | GG+CC+ AA  GT+GG+ AG | 0.72 | | 2.77×${10}^{-2}$ | 0 | NA | NA | | NA | 0.092 |
|  | rs469876+rs28096+rs17482078 | 3 | GG+AA+ TT  AG+CT+ AA | 1.42 | | 2.86×${10}^{-2}$ | 1 | AG+CT+ AG | -1.40 | | 7.81×${10}^{-2}$ | 0.123 |
|  | rs26653+rs28096+rs17482078 | 4 | CC+TT+ AA  GG+CT+ AG | 1.42 | | 2.90×${10}^{-2}$ | 0 | NA | NA | | NA | 0.064 |
|  | rs17481856+ rs10050860+rs28096 | 4 | AA+TT+AA  AG+CT+AG | 1.42 | | 2.93×${10}^{-2}$ | 1 | AG+CT+ AG | -0.48 | | 9.44×${10}^{-2}$ | 0.135 |
|  | rs1065407+rs2287987+rs27044 | 4 | GG+CC+GG  GT+TT+ CG | 0.45 | | 6.26×${10}^{-1}$ | 1 | TT+CT+ CG | -0.46 | | 2.95×${10}^{-2}$ | 0.115 |
|  | rs27044+rs10050860+ rs17482078 | 4 | GG+CT+ TT  CG+TT+ CT | 0.71 | | 1.98×${10}^{-1}$ | 1 | CG+TT+ CT | -0.38 | | 3.08×${10}^{-2}$ | 0.002 |
|  | rs1065407+rs2287987+rs469876 | 2 | GG+CC+ GG  GT+TT+ AA | 0.04 | | 9.77×${10}^{-1}$ | 1 | TT+CT+AG | -0.92 | | 3.18×${10}^{-2}$ | 0.229 |
|  |  |  |  |  | |  |  |  |  | |  |  |
|  |  |  |  |  | |  |  |  |  | |  |  |
| #4 Interaction Effects studies | rs30187+rs10050860+rs469876+rs17481856 | 3 | CC+TT+ AA+AA  TT+CT+ AG+AG | 12.76 | | 1.94×${10}^{-10}$ | 0 | NA | NA | | NA | 0.189 |
|  | rs30187+rs10050860+rs27434+rs469876 | 4 | CC+TT+ AA+AA  TT+CT+ AG+AG | 12.76 | | 1.94×${10}^{-10}$ | 0 | NA | NA | | NA | 0.228 |
|  | rs2287987+rs10050860+rs27044+rs27434 | 3 | CT+TT+ NA+CC  TT+AG+ NA+GG | 12.74 | | 2.08×${10}^{-10}$ | 0 | NA | NA | | NA | 0.064 |
|  | rs2287987+rs10050860+rs27044+rs17481856 | 3 | CC+NA+ GG+AA  TT+NA+ CG+AG | 12.74 | | 2.10×${10}^{-10}$ | 1 | CT+NA+ CG+AG | -0.47 | | 9.36E×${10}^{-2}$ | 0.048 |
|  | rs2287987+rs27044+rs27434+rs17481856 | 3 | CC+AA+ AA+CC  GG+AG+ AG+CT | 12.74 | | 2.13×${10}^{-10}$ | 0 | NA | NA | | NA | 0.110 |
|  | rs2287987+rs10050860+rs27434+rs17481856 | 3 | AA+AA+ CC+NA  AG+AG+ TT+NA | 12.74 | | 2.13×${10}^{-10}$ | 0 | NA | NA | | NA | 0.043 |
|  | rs2287987+rs30187+rs10050860+rs17481856 | 3 | AA+CC+ NA+TT  AG+TT+ NA+CT | 12.74 | | 2.13×${10}^{-10}$ | 0 | NA | NA | | NA | 0.047 |
|  | rs2287987+rs30187+rs10050860+rs27434 | 4 | CC+NA+ CC+AA  TT+NA+ TT+AG | 12.74 | | 2.13×${10}^{-10}$ | 0 | NA | NA | | NA | 0.040 |
|  | rs1065407+rs2287987+rs27434+rs17481856 | 1 | CC+AA+ GG+AA | 12.73 | | 2.15×${10}^{-10}$ | 1 | CT+AG+ TT+AG | -0.69 | | 6.89×${10}^{-2}$ | 0.121 |
|  | rs1065407+rs2287987+rs30187+rs17481856 | 1 | GG+CC+ TT+AA | 12.73 | | 2.15×${10}^{-10}$ | 1 | TT+CT+ CT+AG | -0.69 | | 6.89×${10}^{-2}$ | 0.158 |
|  | rs1065407+rs30187+rs17481856+rs13167972 | 6 | GG+CC+ AA+GG  GT+TT+ AG+AA | 12.73 | | 2.16×${10}^{-10}$ | 1 | TT+CT+ AG+AG | -0.75 | | 6.80×${10}^{-2}$ | 0.163 |
|  | rs1065407+rs27044+rs17481856+rs13167972 | 7 | GG+AA+ GG+GG  GT+AG+ AA+CG | 12.73 | | 2.16×${10}^{-10}$ | 1 | TT+AG+ AG+CG | -0.76 | | 6.55×${10}^{-2}$ | 0.164 |
|  | rs2287987+rs27044+rs469876+rs17481856 | 4 | AA+CC+ CC+AA  AG+GG+ TT+AG | 12.73 | | 2.18×${10}^{-10}$ | 1 | AG+CG+ CT+AG | -0.83 | | 7.17×${10}^{-2}$ | 0.089 |
|  | rs2287987+rs10050860+rs27044+rs469876 | 6 | CC+CC+ AA+NA  GG+TT+ AG+NA | 12.73 | | 2.20×${10}^{-10}$ | 0 | NA | NA | | NA | 0.027 |
|  | rs2287987+rs10050860+rs469876+rs17481856 | 4 | CC+AA+ NA+AA  TT+AG+ NA+AG | 12.73 | | 2.20×${10}^{-10}$ | 1 | CT+AG+ NA+AG | -0.83 | | 7.17×${10}^{-2}$ | 0.052 |
|  | rs1065407+rs2287987+rs10050860+rs17481856 | 3 | CC+NA+ AA+GG  TT+NA+ AG+GT | 12.73 | | 2.20×${10}^{-10}$ | 1 | CT+NA+ AG+TT | -0.59 | | 4.12×${10}^{-2}$ | 0.073 |
|  | rs1065407+rs2287987+rs10050860+rs26653 | 3 | CC+NA+ GG+GG  TT+NA+ GT+CG | 12.73 | | 2.20×${10}^{-10}$ | 0 | NA | NA | | NA | 0.055 |
|  | rs1065407+rs2287987+rs10050860+rs27434 | 3 | CC+NA+ GG+AA  TT+NA+ GT+AG | 12.72 | | 2.23×${10}^{-10}$ | 0 | CT+NA+ TT+AG | NA | | NA | 0.073 |
|  | rs1065407+rs2287987+rs30187+rs10050860 | 3 | CC+NA+ GG+TT  TT+NA+ GT+CT | 12.72 | | 2.23×${10}^{-10}$ | 1 | CT+NA+ TT+CT | -0.39 | | 5.95×${10}^{-2}$ | 0.070 |
|  | rs1065407+rs27044+rs27434+rs13167972 | 15 | GG+GG+ AA+GG  GT+CG+ AG+AA | 1.09 | | 3.78×${10}^{-4}$ | 1 | TT+CG+ AG+AG | -0.70 | | 9.30×${10}^{-2}$ | 0.291 |
|  | rs1065407+rs27044+rs26653+rs13167972 | 17 | GG+GG+ AA+CC  GT+CG+ AG+GG | 1.02 | | 6.74×${10}^{-4}$ | 0 | NA | NA | | NA | 0.057 |
|  | rs1065407+rs26653+rs27434+rs13167972 | 13 | GG+GG+ CC+GG  GT+AA+ CG+AG | 1.00 | | 9.36×${10}^{-4}$ | 0 | NA | NA | | NA | 0.071 |
|  | rs27044+rs26653+rs27434+rs13167972 | 11 | GG+CC+ GG+GG  AG+CG+ AG+CG | 1.07 | | 9.90×${10}^{-4}$ | 0 | NA | NA | | NA | 0.072 |
|  | rs1065407+rs26653+rs17481856+rs13167972 | 12 | GG+CC+ GG+AA  AA+GG+ GT+AG | 1.00 | | 1.23×${10}^{-3}$ | 1 | AG+CG+ TT+AG | -0.77 | | 9.84×${10}^{-2}$ | 0.090 |
|  | rs1065407+rs26653+rs28096+rs13167972 | 20 | GG+CC+ GG+ AA  AA+GG+ GT+AG | 0.93 | | 1.28×${10}^{-3}$ | 0 | NA | NA | | NA | 0.070 |
|  | rs1065407+rs10050860+rs27044+rs26653 | 7 | CC+GG+ TT+GG  GG+GT+ CT+CG | 0.99 | | 1.34×${10}^{-3}$ | 1 | CG+TT+ CT+CG | -0.83 | | 5.47×${10}^{-2}$ | 0.087 |
|  | rs27044+rs26653+rs469876+rs17482078 | 8 | CC+CC+ AA+TT  GG+GG+ AG+CT | 0.82 | | 1.56×${10}^{-3}$ | 0 | NA | NA | | NA | 0.040 |
|  | rs1065407+rs469876+rs28096+rs13167972 | 18 | GG+GG+ AA+GG  AA+GT+ AG+AA | 0.91 | | 1.75×${10}^{-3}$ | 1 | AG+TT+ AG+AG | -1.94 | | 7.01×${10}^{-2}$ | 0.528 |
|  | rs10050860+rs27044+rs26653+rs27434 | 5 | TT+GG+ CC+GG  CT+CG+ CG+AG | 0.96 | | 2.03×${10}^{-3}$ | 0 | NA | NA | | NA | 0.068 |
|  | rs1065407+rs27044+rs28096+rs13167972 | 12 | GG+GG+ AA+GG  CG+GT+ AG+AA | 0.94 | | 2.04×${10}^{-3}$ | 0 | NA | NA | | NA | 0.169 |
|  | rs10050860+rs27044+rs26653+rs469876 | 7 | CC+TT+ CC+AA  GG+CT+ GG+AG | 0.80 | | 2.09×${10}^{-3}$ | 1 | CG+CT+CG+AG | -0.50 | | 7.89×${10}^{-2}$ | 0.039 |
|  | rs27044+rs26653+rs27434+rs17482078 | 7 | GG+CC+ GG+TT  CG+CG+ AG+CT | 0.92 | | 2.43×${10}^{-3}$ | 0 | NA | NA | | NA | 0.052 |
|  | rs27044+rs26653+rs469876+rs17481856 | 8 | CC+CC+ AA+AA  GG+GG+ AG+AG | 0.71 | | 2.48×${10}^{-3}$ | 0 | NA | NA | | NA | 0.055 |
|  | rs27044+rs26653+rs28096+rs17482078 | 9 | CC+CC+ AA+TT  GG+GG+ AG+CT | 1.14 | | 2.53×${10}^{-3}$ | 0 | NA | NA | | NA | 0.063 |
|  | rs1065407+rs27044+rs26653+rs17481856 | 9 | GG+CC+ CC+AA  GT+GG+ GG+AG | 0.90 | | 2.54×${10}^{-3}$ | 0 | NA | NA | | NA | 0.068 |
|  | rs30187+rs26653+rs28096+rs13167972 | 18 | CC+CC+ AA+GG  TT+GG +AG+ AA | 0.90 | | 3.06×${10}^{-3}$ | 0 | NA | NA | | NA | 0.145 |
|  | rs27044+rs27434+rs469876+rs13167972 | 13 | AA+AA+ CC+AA  AG+GG+ AG+AG | 0.68 | | 3.12×${10}^{-3}$ | 1 | AG+CG+ AG+AG | -0.55 | | 4.26×${10}^{-2}$ | 0.120 |
|  | rs1065407+rs27434+rs469876+rs13167972 | 11 | GG+GG+ AA+TT  AA+AA+ AG+GT | 0.77 | | 3.18×${10}^{-3}$ | 1 | AG+AG+ AG+ TT | -0.62 | | 3.44×${10}^{-2}$ | 0.174 |
|  | rs30187+rs27044+rs469876+rs17481856 | 5 | TT+CC+ AA+AA  CT+GG+ AG+AG | 0.62 | | 3.35×${10}^{-3}$ | 0 | NA | NA | | NA | 0.170 |
|  | rs10050860+rs27044+rs26653+rs28096 | 8 | TT+CC+ CC+AA  CT+GG+ GG+AG | 1.10 | | 3.58×${10}^{-3}$ | 0 | NA | NA | | NA | 0.077 |
|  | rs1065407+rs27434+rs28096+rs13167972 | 13 | GG+GG+ AA+GG  GT+AG+ AG+AA | 0.85 | | 3.58×${10}^{-3}$ | 0 | NA | NA | | NA | 0.246 |
|  | rs10050860+rs27044+rs26653+rs17482078 | 5 | CT+CC+ CC+TT  TT+GG+ GG+CT | 0.85 | | 4.10×${10}^{-3}$ | 0 | NA | NA | | NA | 0.002 |
|  | rs1065407+rs27044+rs26653+rs469876 | 9 | GG+CC+ CC+AA  GT+GG+ GG+AG | 0.84 | | 4.92×${10}^{-3}$ | 1 | TT+CG+ CG+AG | -0.52 | | 8.98×${10}^{-2}$ | 0.067 |
|  | rs1065407+rs27434+rs17481856+rs13167972 | 7 | GG+GG+ AA+GG  GT+AG+ AG+AA | 0.88 | | 5.09×${10}^{-3}$ | 1 | TT+AG+ AG+AG | -0.76 | | 6.67×${10}^{-2}$ | 0.141 |
|  | rs1065407+rs2287987+rs469876+rs17481856 | 3 | GG+CC+ AA+AA  GT+TT+ AG+AG | -0.66 | | 5.93×${10}^{-1}$ | 2 | TT+CT+ AG+AG  GT+CT+AA+AA | -0.92 | | 5.32×${10}^{-3}$ | 0.148 |
|  | rs30187+rs27044+rs26653+rs13167972 | 13 | CC+GG+ CC+GG  CT+CG+ GG+AG | 0.87 | | 5.49×${10}^{-3}$ | 0 | NA | NA | | NA | 0.126 |
|  | rs30187+rs27044+rs27434+rs469876 | 7 | TT+CC+ AA+AA  CT+CG+ AG+AG | 0.58 | | 5.51×${10}^{-3}$ | 0 | NA | NA | | NA | 0.073 |
|  | rs1065407+rs30187+rs28096+rs13167972 | 14 | TT+GG+ AA+GG  CT+GT+ AG+AA | 2.09 | | 5.83×${10}^{-3}$ | 1 | CT+TT+ AG+AG | -0.47 | | 9.63×${10}^{-2}$ | 0.252 |
|  | rs27044+rs26653+rs28096+rs13167972 | 20 | GG+CC+ AA+GG  CG+GG+ AG+AA | 0.76 | | 5.86×${10}^{-3}$ | 0 | NA | NA | | NA | 0.071 |
|  | rs27044+rs469876+rs17481856+rs28096 | 5 | CC+AA+ AA+GG  GG+AG+ AG+AA | 0.68 | | 6.29×${10}^{-3}$ | 1 | CG+AG+ AG+AG | -1.22 | | 6.49×${10}^{-2}$ | 0.065 |
|  | rs10050860+rs27044+rs26653+rs17481856 | 5 | TT+TT+ CC+AA  CT+GG+ GG+AG | 0.79 | | 6.73×${10}^{-3}$ | 0 | NA | NA | | NA | 0.032 |
|  | rs27044+rs27434+rs28096+rs13167972 | 17 | GG+AA +AA+GG  CG+AG+ AG+AA | 0.72 | | 6.78×${10}^{-3}$ | 0 | NA | NA | | NA | 0.260 |
|  | rs1065407+rs27044+rs26653+rs27434 | 8 | GG+GG+ CC+GG  GT+GG+ CG+AG | 0.87 | | 6.88×${10}^{-3}$ | 0 | NA | NA | | NA | 0.092 |
|  | rs1065407+rs2287987+rs27044+rs26653 | 6 | GG+CC+ GG+CC  GT+CT+ CG+GG | 1.08 | | 6.84×${10}^{-2}$ | 2 | TT+TT+ CG+CG  CT+CT+ GG+GG | -0.89 | | 7.25×${10}^{-3}$ | 0.088 |
|  | rs1065407+rs2287987+rs26653+rs17481856 | 2 | GG+CC+ CC+AA  TT+CT+ GG+AG | 0.04 | | 9.79×${10}^{-1}$ | 2 | TT+TT+ CG+ AG  GT+CT+ GG+AA | -0.89 | | 7.25×${10}^{-3}$ | 0.106 |
|  | rs1065407+rs2287987+rs30187+rs26653 | 7 | GG+CC+ CC+CC  GT+CT+ TT+CG | 0.53 | | 1.94×${10}^{-1}$ | 2 | TT+TT+CT+ GG  GT+CT+TT+CG | -0.88 | | 7.50×${10}^{-3}$ | 0.184 |
|  | rs27044+rs26653+rs17481856+rs28096 | 9 | CC+CC+ AA+AA  GG+GG+ AG+AG | 0.68 | | 7.66×${10}^{-3}$ | 0 | NA | NA | | NA | 0.042 |
|  | rs27044+rs26653+rs17481856+rs17482078 | 7 | CC+CC+ AA+TT  GG+GG+ AG+AG | 0.76 | | 7.81×${10}^{-3}$ | 0 | NA | NA | | NA | 0.041 |
|  | rs2287987+rs469876+rs17481856+rs28096 | 4 | CC+AA+ AA+AA  CT+AG+ AG+AG | 0.03 | | 9.78×${10}^{-1}$ | 2 | TT+AG +AG+AG  CT+AA+ AA+ AA | -1.17 | | 8.12×${10}^{-3}$ | 0.188 |
|  | rs26653+rs17481856+rs28096+rs13167972 | 10 | CC+AA+ AA+GG  GG+AG+ AG+ AA | 0.83 | | 8.35×${10}^{-3}$ | 0 | NA | NA | | NA | 0.087 |
|  | rs1065407+rs30187+rs26653+rs13167972 | 16 | GG+CC+ CC+GG  GT+TT+ GG+AA | 0.75 | | 8.39×${10}^{-3}$ | 0 | NA | NA | | NA | 0.143 |
|  | rs1065407+rs17481856+rs28096+rs13167972 | 11 | GG+AA+ AA+GG  GT+AG+ AG+ AA | 2.54 | | 1.48×${10}^{-2}$ | 2 | TT+AG+ AG+AG  GT+AA+ AA+AA | -0.73 | | 8.48×${10}^{-3}$ | 0.127 |
|  | rs2287987+rs30187+rs27044+rs13167972 | 13 | GG+CC+ CC+TT  AA+GG+ CT+CT | 0.78 | | 8.56×${10}^{-3}$ | 0 | NA | NA | | NA | 0.200 |
|  | rs1065407+rs27044+rs469876+rs13167972 | 11 | GG+CC +AA+AA  GT+GG+ AG+AG | 2.71 | | 9.10×${10}^{-3}$ | 0 | NA | NA | | NA | 0.083 |
|  | rs1065407+rs27044+rs26653+rs17482078 | 8 | GG+GG+ CC+TT  GT+CG+ GG+CT | 1.16 | | 9.13×${10}^{-3}$ | 1 | TT+CG+CG+CT | -0.84 | | 5.37×${10}^{-2}$ | 0.074 |
|  | rs27044+rs469876+rs28096+rs13167972 | 9 | CC+AA+ AA+AA  GG+AG+ AG+AG | 0.73 | | 9.80×${10}^{-3}$ | 0 | NA | NA | | NA | 0.075 |
|  | rs27044+rs26653+rs27434+rs469876 | 6 | CC+CC+ GG+AA  GG+GG+ AG+AG | 0.68 | | 1.02×${10}^{-2}$ | 0 | NA | NA | | NA | 0.051 |
|  | rs27044+rs27434+rs469876+rs28096 | 5 | CC+AA+ AA+GG  GG+AG+ AG+AA | 0.63 | | 1.08×${10}^{-2}$ | 1 | CG+AG+ AG+AG | -1.11 | | 9.99×${10}^{-2}$ | 0.053 |
|  | rs1065407+rs30187+rs469876+rs13167972 | 12 | GG+CC+ AA+GG  GT+TT+ AG+AA | 1.94 | | 1.11×${10}^{-2}$ | 1 | TT+CT+ AG+AG | -0.50 | | 8.75×${10}^{-2}$ | 0.135 |
|  | rs1065407+rs10050860+rs26653+rs13167972 | 11 | GG+TT+ CC+GG  GT+CT+ GG+AA | 1.93 | | 1.15×${10}^{-2}$ | 0 | NA | NA | | NA | 0.089 |
|  | rs1065407+rs2287987+rs26653+rs469876 | 5 | GG+CC+ CC+GG  GT+CT+ GG+AA | -0.66 | | 4.49×${10}^{-1}$ | 2 | TT+TT+ CG+AG  GT+CT+GG+AG | -0.65 | | 1.18×${10}^{-2}$ | 0.219 |
|  | rs30187+rs27044+rs469876+rs13167972 | 11 | TT+CC+ AA+AA  CT+GG +AG+AG | 0.84 | | 1.18×${10}^{-2}$ | 1 | CT+CG+ AG+AG | -0.50 | | 6.13×${10}^{-2}$ | 0.228 |
|  | rs30187+rs26653+rs17481856+rs13167972 | 6 | CC+CC+ AA+GG  CT+CG+ AG+AA | 0.84 | | 1.22×${10}^{-2}$ | 0 | NA | NA | | NA | 0.066 |
|  | rs10050860+rs26653+rs27434+rs469876 | 5 | TT+CC+ GG+GG  CT+CG+ AG+AA | 0.70 | | 5.44×${10}^{-2}$ | 2 | CT+GG+ AA+AG  TT+CG+AG+AA | -0.53 | | 1.24×${10}^{-2}$ | 0.079 |
|  | rs1065407+rs10050860+rs469876+rs13167972 | 12 | GT+TT+  GG+GG  TT+CT+ AA+AA | 1.60 | | 1.25×${10}^{-2}$ | 1 | TT+CT+ AG+AG | -0.88 | | 4.08×${10}^{-2}$ | 0.169 |
|  | rs1065407+rs27044+rs26653+rs28096 | 11 | GG+GG+ CC+AA  GT+CG+ GG+AG | 0.77 | | 1.27×${10}^{-2}$ | 0 | NA | NA | | NA | 0.098 |
| #5 Interaction Effects | rs2287987+rs10050860+rs27044+rs27434+rs17481856 | 5 | CC+NA+ CC+AA+ AA  TT+NA+ GG+AG+ AG | 12.73 | | 2.20×${10}^{-10}$ | 0 | NA | NA | | NA | 0.046 |
|  | rs1065407+rs2287987+rs10050860+rs27434+rs17481856 | 3 | GG+CC+ NA+GG+ AA  GT+TT+ NA+AG+ AG | 12.72 | | 2.23×${10}^{-10}$ | 1 | TT+CT+ NA+AG+ AG | -0.69 | | 6.89×${10}^{-2}$ | 0.084 |
|  | rs1065407+rs2287987+rs30187+rs10050860+rs17481856 | 3 | GG+CC+ CT+NA+ AG  GT+CT+ CT+NA+ AG | 12.72 | | 2.23×${10}^{-10}$ | 1 | TT+CT+ CT+NA +AG | -0.69 | | 6.89×${10}^{-2}$ | 0.077 |
|  | rs1065407+rs27044+rs27434+rs469876+rs13167972 | 23 | GG+CC+ AA+AA+ AA  GT+GG+ AG+AG+ AG | 1.08 | | 2.23×${10}^{-4}$ | 1 | TT+CG+ AG+AG+ AG | -0.55 | | 6.06×${10}^{-2}$ | 0.165 |
|  | rs1065407+rs27044+rs26653+rs17481856+rs13167972 | 20 | GG+CC+ CC+AA+ AA  GT+GG+ GG+AG+ AG | 1.01 | | 5.34×${10}^{-4}$ | 0 | NA | NA | | NA | 0.039 |
|  | rs1065407+rs26653+rs17481856+rs28096+rs13167972 | 24 | GG+CC+ AA+AA+ GG  GT+GG+ AG+AG+ AA | 0.93 | | 1.01×${10}^{-3}$ | 0 | NA | NA | | NA | 0.083 |
|  | rs1065407+rs30187+rs27044+rs469876+rs13167972 | 19 | GG+TT+ CC+AA+ AA  GT+CT+ GG+AG+ AG | 1.99 | | 1.45×${10}^{-3}$ | 1 | TT+CT+ CG+AG+ AG | -0.50 | | 8.75×${10}^{-2}$ | 0.287 |
|  | rs1065407+rs27044+rs26653+rs27434+rs13167972 | 26 | GG+GG+ CC+GG+ GG  GT+CG+ CG+AG+ AA | 0.84 | | 1.53×${10}^{-3}$ | 0 | NA | NA | | NA | 0.074 |
|  | rs1065407+rs27434+rs17481856+rs28096+rs13167972 | 17 | GG+AA+ AA+AA+ GG  GT+AG+ AG+AG+ AA | 0.91 | | 1.76×${10}^{-3}$ | 1 | TT+AG+ AG+AG+ AG | -0.77 | | 9.99×${10}^{-2}$ | 0.133 |
|  | rs1065407+rs27044+rs27434+rs17481856+rs13167972 | 18 | GG+GG+ AA+AA+ GG  GT+CG+ AG+AG+ AA | 0.91 | | 1.76×${10}^{-3}$ | 1 | TT+CG+ AG+AG+ AG | -0.70 | | 9.30×${10}^{-2}$ | 0.207 |
|  | rs1065407+rs2287987+rs26653+rs469876+rs17481856 | 11 | GG+CC+ CC+GG+ AA  GT+TT+ GG+AA+ AG | -0.25 | | 6.44×${10}^{-3}$ | 3 | TT+CT+ CG+AG+ AG  GT+TT+ GG+AA+ AA | -0.96 | | 1.81×${10}^{-3}$ | 0.161 |
|  | rs10050860+rs27044+rs26653+rs27434+rs469876 | 10 | TT+CC+ CC+GG+ AA  CT+GG+ CG+AG+ AG | 0.80 | | 1.99×${10}^{-3}$ | 0 | NA | NA | | NA | 0.088 |
|  | rs1065407+rs30187+rs27044+rs28096+rs13167972 | 24 | GG+TT+ CC+AA+ GG  GT+CT+ GG+AG+ AA | 1.43 | | 2.11×${10}^{-3}$ | 0 | NA | NA | | NA | 0.412 |
|  | rs1065407+rs27044+rs26653+rs28096+rs13167972 | 34 | GG+GG+ GG+AA+ AA  GT+CG+  GG+AG+ AG | 0.78 | | 2.16×${10}^{-3}$ | 0 | NA | NA | | NA | 0.080 |
|  | rs27044+rs26653+rs27434+rs469876+rs17482078 | 12 | CC+CC+ GG+AA+ TT  GG+CG+ AG+AG+ CT | 0.78 | | 2.30×${10}^{-3}$ | 0 | NA | NA | | NA | 0.081 |
|  | rs1065407+rs27044+rs27434+rs28096+rs13167972 | 29 | GG+GG+ AA+AA+ GG  TT+CG+ AG+AG+ AA | 0.79 | | 2.38×${10}^{-3}$ | 0 | NA | NA | | NA | 0.310 |
|  | rs1065407+rs10050860+rs27044+rs26653+rs469876 | 15 | GG+TT+ CC+CC+ AA  GT+CT+ GG+GG+ AG | 0.85 | | 2.44×${10}^{-3}$ | 1 | TT+CT+ CG+CG+ AG | -0.77 | | 7.88×${10}^{-2}$ | 0.067 |
|  | rs1065407+rs10050860+rs27044+rs26653+rs17481856 | 10 | GG+TT+ CC+CC+ AA  GT+CT+ GG+GG+ AG | 0.90 | | 2.54×${10}^{-3}$ | 1 | TT+CT+ CG+CG+ AG | -0.83 | | 5.47×${10}^{-2}$ | 0.051 |
|  | rs1065407+rs10050860+rs27044+rs26653+rs17482078 | 10 | TT+CT+ GG+CC+ TT  GT+TT+ GT+GG+ CT | 0.89 | | 2.65×${10}^{-3}$ | 1 | TT+TT+ CG+CG+ CT | -0.84 | | 5.37×${10}^{-2}$ | 0.002 |
|  | rs1065407+rs26653+rs27434+rs28096+rs13167972 | 27 | TT+CC+ GG+GG+ GG  GT+CG+ AG+AA+ AA | 0.79 | | 3.04×${10}^{-3}$ | 0 | NA | NA | | NA | 0.057 |
|  | rs1065407+rs30187+rs17481856+rs28096+rs13167972 | 16 | GG+CC+ AA+AA+ GG  GT+TT+ AG+AG+ AA | 2.22 | | 3.15×${10}^{-3}$ | 0 | NA | NA | | NA | 0.149 |
|  | rs10050860+rs27044+rs26653+rs469876+rs17482078 | 10 | CT+CC+ CC+AA+ TT  TT+GG+ GG+ AG+ CT | 0.74 | | 3.70×${10}^{-3}$ | 0 | NA | NA | | NA | 0.002 |
|  | rs1065407+rs30187+rs10050860+rs28096+rs13167972 | 22 | GT+TT+ TT+AA+ GG  TT+TT +CT+AG+ AA | 1.47 | | 3.79×${10}^{-3}$ | 1 | TT+CT+ CT+AG+ AG | -0.47 | | 9.63×${10}^{-2}$ | 0.281 |
|  | rs10050860+rs27044+rs26653+rs469876+rs17481856 | 11 | TT+CC+ CC+AA+ AA  CT+TT+ GG+AG+ AG | 0.73 | | 3.88×${10}^{-3}$ | 0 | NA | NA | | NA | 0.056 |
|  | rs10050860+rs27044+rs26653+rs27434+rs17482078 | 8 | CT+GG+ CC+GG+ TT  TT+CG+ CG+AG+ CT | 0.86 | | 3.93×${10}^{-3}$ | 0 | NA | NA | | NA | 0.002 |
|  | rs1065407+rs10050860+rs27044+rs28096+rs13167972 | 18 | GT+TT+ GG+AA+ GG  TT+CT+ CG+AG+ AA | 1.80 | | 4.28×${10}^{-3}$ | 0 | NA | NA | | NA | 0.190 |
|  | rs30187+rs27044+rs26653+rs17481856+rs13167972 | 16 | CC+GG+ CC+AA+ GG  CT+CG+ GG+AG+ AG | 0.87 | | 4.37×${10}^{-3}$ | 0 | NA | NA | | NA | 0.060 |
|  | rs1065407+rs30187+rs26653+rs28096+rs13167972 | 31 | GG+CC+ CC+GG+ GG  GT+TT+ TT+AA+ AA | 0.75 | | 4.44×${10}^{-3}$ | 0 | NA | NA | | NA | 0.134 |
|  | rs27044+rs26653+rs469876+rs17481856+rs17482078 | 13 | CC+CC+ AA+AA+ TT  GG+GG+ AG+AG+ CT | 0.71 | | 4.45×${10}^{-3}$ | 0 | NA | NA | | NA | 0.052 |
|  | rs1065407+rs30187+rs27044+rs26653+rs13167972 | 27 | GG+CC+ GG+CC+ GG  GT+CT+ CG+GG+ AG | 0.75 | | 4.45×${10}^{-3}$ | 0 | NA | NA | | NA | 0.105 |
|  | rs1065407+rs2287987+rs26653+rs27434+rs17481856 | 7 | GG+CC+ CC+AG+AA  GT+TT+ CG+AG+ AG | 0.53 | | 1.94×${10}^{-1}$ | 2 | TT+CT+ GG+AA+ AG  GT+TT+ CG+AG+ AA | -0.96 | | 4.53×${10}^{-3}$ | 0.081 |
|  | rs1065407+rs2287987+rs27044+rs26653+rs27434 | 11 | GG+TT+ GG+CC+ TT  GT+CT+ CG+CG+ CT | 0.62 | | 1.11×${10}^{-1}$ | 2 | TT+CT+CG+AA+CT  GT+TT+GG+AG+TT | -0.96 | | 4.53×${10}^{-3}$ | 0.029 |
|  | rs1065407+rs30187+rs28096+rs13167972+rs17482078 | 24 | GT+TT+ AA+GG+ TT  TT+CT+ AG+AA+ CT | 1.33 | | 4.67×${10}^{-3}$ | 1 | TT+CT+ AG+ AG+ CT | -0.47 | | 9.36×${10}^{-2}$ | 0.223 |
|  | rs1065407+rs2287987+rs30187+rs26653+rs27434 | 9 | GG+CC+ TT+CC+ GG  GT+TT+ CT+CT+ AG | 0.50 | | 2.03×${10}^{-1}$ | 2 | TT+CT+ CT+GG+ AA  GT+TT+ TT+CG+ AG | -0.96 | | 4.69×${10}^{-3}$ | 0.087 |
|  | rs1065407+rs2287987+rs17481856+rs28096+rs13167972 | 16 | GT+CC+ AA+GG+ GG  TT+TT+ AG+AG+ AA | 1.17 | | 4.43×${10}^{-2}$ | 2 | TT+CT+ AG+AG+ AG  GT+TT+AA+AA+ AA | -0.80 | | 4.72×${10}^{-3}$ | 0.120 |
|  | rs1065407+rs469876+rs17481856+rs28096+rs13167972 | 22 | GG+GG+ AA+AA+ GG  GT+AA+ AG+AG+ AA | 0.78 | | 4.91×${10}^{-3}$ | 1 | TT+AG+ AG+AG+ AG | -1.94 | | 7.01×${10}^{-2}$ | 0.147 |
|  | rs1065407+rs27044+rs28096+rs13167972+rs17482078 | 20 | GT+GG+ AA+GG+ TT  TT+CG+ AG+AA+ CT | 1.57 | | 4.95×${10}^{-3}$ | 0 | NA | NA | | NA | 0.168 |
|  | rs27044+rs26653+rs27434+rs17481856+rs13167972 | 16 | GG+CC+ GG+AA +GG  CG+CG+ AG+AG+ AG | 0.84 | | 5.13×${10}^{-3}$ | 0 | NA | NA | | NA | 0.081 |
|  | rs1065407+rs26653+rs469876+rs28096+rs13167972 | 30 | GG+CC+ GG+AA+ GG  GT+GG+ AA+AG+ AA | 1.02 | | 5.23×${10}^{-3}$ | 0 | NA | NA | | NA | 0.152 |
|  | rs1065407+rs2287987+rs469876+rs17481856+rs13167972 | 16 | GG+CC+ AA+AA+ GG  GT+TT+ AG+AG+ AA | 0.85 | | 9.02×${10}^{-2}$ | 2 | TT+CT+ AG+AG+ AG  GT+TT+ AA+AA+ AA | -0.91 | | 5.71×${10}^{-3}$ | 0.152 |
|  | rs30187+rs27044+rs26653+rs27434+rs13167972 | 15 | TT+GG+ CC+GG+ GG  CT+CG+ CG+AG+ AG | 0.84 | | 5.97×${10}^{-3}$ | 0 | NA | NA | | NA | 0.106 |
|  | rs1065407+rs30187+rs26653+rs17481856+rs13167972 | 17 | GG+CC+ CC+AA+ GG  GT+CT+ CG+AG+ AA | 0.78 | | 6.00×${10}^{-3}$ | 0 | NA | NA | | NA | 0.066 |
|  | rs30187+rs27044+rs27434+rs469876+rs17481856 | 8 | TT+CC+ GG+AA+ GG  CT+GG+ AG+AG+ AA | 0.57 | | 6.20×${10}^{-3}$ | 0 | NA | NA | | NA | 0.108 |
|  | rs27044+rs27434+rs469876+rs17481856+rs13167972 | 15 | CC+AA+ AA+AA+ AA  GG+AG+ AG+AG+ AG | 0.62 | | 6.52×${10}^{-3}$ | 1 | CG+AG+ AG+AG+ AG | -0.55 | | 4.26×${10}^{-2}$ | 0.221 |
|  | rs1065407+rs30187+rs27434+rs28096+rs13167972 | 18 | GG+CC+ GG+GG+ GG  TT+CT+ AA+AG+ AA | 1.52 | | 6.65×${10}^{-3}$ | 0 | NA | NA | | NA | 0.338 |
|  | rs1065407+rs27044+rs26653+rs469876+rs13167972 | 24 | GT+CC+ CC+ AA+AA  TT+GG+ GG+AG+ AG | 0.75 | | 6.87×${10}^{-3}$ | 2 | TT+CG+ CG+AG+ AG  GT+GG +GG+AA+ AA | -0.72 | | 1.09×${10}^{-2}$ | 0.080 |
|  | rs1065407+rs26653+rs27434+rs17481856+rs13167972 | 16 | GT+CC+ GG+AA+ GG  TT+CG+ AG+AG+ AA | 0.75 | | 6.89×${10}^{-3}$ | 1 | TT+GG+ AA+AG+ AG | -0.77 | | 9.99×${10}^{-2}$ | 0.086 |
|  | rs1065407+rs10050860+rs27044+rs27434+rs13167972 | 19 | GT+TT+ GG+AA+ GG  TT+CT+ CG+AG+ AA | 1.52 | | 6.93×${10}^{-3}$ | 1 | TT+CT+ CG+AG+ AG | -0.70 | | 9.30×${10}^{-2}$ | 0.246 |
|  | rs1065407+rs27044+rs469876+rs28096+rs13167972 | 26 | GT+CC+ AA+GG+ AA  TT+GG+ AG+AA+ AG | 0.70 | | 6.98×${10}^{-3}$ | 1 | TT+CG+ AG+AG +AG | -1.94 | | 7.01×${10}^{-2}$ | 0.086 |
|  | rs1065407+rs2287987+rs26653+rs17481856+rs17482078 | 6 | GT+TT+ CC+AA+ TT  TT+CT+ GG+AG+CT | -0.67 | | 4.44×${10}^{-1}$ | 2 | TT+CT+ CG+AG+ AG  GT+TT+ GG+AA+ AA | -0.89 | | 7.01×${10}^{-3}$ | 0.038 |
|  | rs1065407+rs2287987+rs27044+rs26653+rs17482078 | 11 | GT+TT+ GG+CC+ TT  TT+CT+ CG+GG+ CT | 0.69 | | 2.78×${10}^{-2}$ | 2 | TT+CT+ CG+CG+ CT  GT+TT+ GG + GG +CT | -0.89 | | 7.01×${10}^{-3}$ | 0.035 |
|  | rs1065407+rs10050860+rs27044+rs26653+rs13167972 | 18 | TT+TT+ GG+CC+ AA  GT+CG+ CT+ GG+AG | 1.51 | | 7.06×${10}^{-3}$ | 1 | GT+CT+ CG+CG+ AG | -0.83 | | 5.68×${10}^{-2}$ | 0.066 |
|  | rs1065407+rs2287987+rs10050860+rs27044+rs26653 | 9 | GT+CC+ NA+GG+ CC  TT+TT+ NA+CG+ GG | 0.72 | | 2.47×${10}^{-2}$ | 2 | TT+CT+ NA+CG+ CG | -0.89 | | 7.25×${10}^{-3}$ | 0.066 |
|  | rs1065407+rs2287987+rs10050860+rs26653+rs17481856 | 4 | GT+CC+ NA+CC+ AA  TT+TT+ NA+GG+ AG | -1.36 | | 2.25×${10}^{-1}$ | 2 | TT+CT+ NA+CG+ AG  CT+TT+NA+GG+AA | -0.89 | | 7.25×${10}^{-3}$ | 0.065 |
|  | rs1065407+rs2287987+rs30187+rs26653+rs17482078 | 11 | GT+TT+ CC+CC+ TT  TT+CT+ TT+CG+ CT | 0.32 | | 3.93×${10}^{-1}$ | 2 | TT+CT+ CT+GG+ CT  GT+TT+ TT+CG+ TT | -0.89 | | 7.25×${10}^{-3}$ | 0.032 |
|  | rs1065407+rs2287987+rs469876+rs17481856+rs17482078 | 7 | GT+CT+ AA+AA+ TT  TT+TT+ AG+AG+ CT | -0.89 | | 2.91×${10}^{-1}$ | 2 | TT+TT+ AG+AG+ AG  GT+CT+AA+AA+ AA | -0.89 | | 7.25×${10}^{-3}$ | 0.018 |
|  | rs10050860+rs27044+rs26653+rs28096+rs17482078 | 11 | CT+CC+ CC+AA+ TT  TT+GG+ GG+AG+ CT | 0.95 | | 7.39×${10}^{-3}$ | 0 | NA | NA | | NA | 0.002 |
|  | rs1065407+rs2287987+rs30187+rs26653+rs17481856 | 8 | GG+CC+ CC+CC+ AA  TT+CT+ CT+CG+ AG | 0.60 | | 1.41×${10}^{-1}$ | 2 | TT+TT+ TT+GG+ AG  GT+CT+CT+CG+AA | -0.88 | | 7.50×${10}^{-3}$ | 0.084 |
|  | rs1065407+rs2287987+rs30187+rs10050860+rs26653 | 9 | GG+CC+ CC+NA+ CC  GT+TT +TT+NA+CG | 0.26 | | 4.94×${10}^{-1}$ | 2 | TT+CT+CT+NA+GG  GT+TT+TT+NA+CG | -0.88 | | 7.50×${10}^{-3}$ | 0.072 |
|  | rs1065407+rs2287987+rs10050860+rs469876+rs17481856 | 6 | GG+CC+ NA+AA+ AA  GT+TT+ NA+AG+ AG | -1.58 | | 1.50×${10}^{-1}$ | 2 | TT+CT+ NA+AG+ AG  GT+TT+ NA+AA+ AA | -0.88 | | 7.50×${10}^{-3}$ | 0.053 |
|  | rs1065407+rs2287987+rs27044+rs469876+rs17481856 | 9 | GT+CC+ CC+AA+ AA  TT+TT+ GG+AG+ AG | -0.18 | | 7.85×${10}^{-1}$ | 2 | TT+CT+CG+AG+AG  GT+TT+GG+AA+AA | -0.88 | | 7.50×${10}^{-3}$ | 0.105 |
|  | rs10050860+rs27044+rs26653+rs17481856+rs28096 | 11 | TT+CC+ CC+AA+ AA  CT+GG+ GG+AG+ AG | 0.94 | | 7.61×${10}^{-3}$ | 0 | NA | NA | | NA | 0.049 |
|  | rs1065407+rs2287987+rs26653+rs17481856+rs13167972 | 13 | GG+CC +CC+GG+GG  GT+TT+ GG+AG+ AA | 1.55 | | 1.65×${10}^{-2}$ | 2 | TT+CT+ CG+AG+ AG  GT_TT+GG+AA+AA | -0.88 | | 7.76×${10}^{-3}$ | 0.080 |
|  | rs1065407+rs30187+rs469876+rs28096+rs13167972 | 29 | TT+CC+ AA+GG+ GG  GT+TT+ AG+AA+ AA | 0.49 | | 1.29×${10}^{-1}$ | 2 | TT+CT+ AG+AG+ AG  GT+TT+AA+AA+AA | -1.69 | | 7.82×${10}^{-3}$ | 0.172 |
|  | rs2287987+rs469876+rs17481856+rs28096+rs17482078 | 9 | CT+AA+ AA+AA+ TT  TT+AG+ AG+AG+ CT | -0.20 | | 7.65×${10}^{-1}$ | 2 | TT+AG+ AG+AG+ CT  CT+AA+ AA+AA+ TT | -1.17 | | 7.93×${10}^{-3}$ | 0.020 |
|  | rs1065407+rs2287987+rs30187+rs26653+rs13167972 | 20 | GG+CC+ CC+CC+ GG  GT+TT+ TT+CG+ AA | 0.42 | | 2.80×${10}^{-1}$ | 2 | TT+CT+ CT+GG+ AG  GT+TT+TT+CG+AA | -0.88 | | 8.02×${10}^{-3}$ | 0.147 |
|  | rs2287987+rs27044+rs469876+rs17481856+rs28096 | 11 | CC+CC+ AA+AA+ GG  TT+GG+ AG+AG+ AG | -0.16 | | 7.96×${10}^{-1}$ | 2 | CT+CG+ AG+AG+ AA  TT+GG+ AA+AA+ AG | -1.17 | | 8.12×${10}^{-3}$ | 0.060 |
|  | rs2287987+rs10050860+rs469876+rs17481856+rs28096 | 8 | CC+AA +AA+AA+NA  TT+AG +AG+AG+NA | -0.49 | | 5.05×${10}^{-1}$ | 2 | CT+AG +AG+AG+NA  TT+AA +AA+AA+NA | -1.17 | | 8.12×${10}^{-3}$ | 0.069 |
|  | rs1065407+rs17481856+rs28096+rs13167972+rs17482078 | 18 | GT+AA +AA+GG+TT  TT+AG +AG+AA+CT | 1.67 | | 8.89×${10}^{-3}$ | 2 | TT+AG +AG+AG+CT  GT+AA +AA+AA +TT | -0.73 | | 8.14×${10}^{-3}$ | 0.121 |
|  | rs1065407+rs10050860+rs17481856+rs28096+rs13167972 | 16 | GT+TT +AA+AA+GG  TT+CT+ AG+AG+ AA | 2.01 | | 8.26×${10}^{-3}$ | 2 | TT+CT+AG+AG+AG  GT+TT+AA+AA+AA | -0.73 | | 8.48×${10}^{-3}$ | 0.118 |
|  | rs1065407+rs10050860+rs26653+rs17481856+rs13167972 | 13 | GG+TT +CC+AA+GG  GT+CT+ GG+AG+ AA | 2.01 | | 8.27×${10}^{-3}$ | 2 | TT+CT+ CG+AG +AG  GT+TT+GG+AA+AA | -0.83 | | 9.95×${10}^{-3}$ | 0.096 |
| #6 Interaction Effects | rs1065407+rs27044+rs27434+rs469876+rs17481856+rs13167972 | 25 | GG+CC +AA+AA+AA+AA  GT+GG +AA+AG+AG+AG | 0.96 | | 6.31×${10}^{-4}$ | 1 | GT+CG +AG+AG+AG+AG | -0.55 | | 6.06×${10}^{-2}$ | 0.226 |
|  | rs1065407+rs2287987+rs26653+rs469876+rs17481856+rs13167972 | 24 | GG+AA +AA+GG+CC+CC  GT+AG+ AG+AA+ GG+TT | 0.42 | | 2.80×${10}^{-1}$ | 3 | TT+AG+ AG+AG+ CG+CT  GT+AA +AA+AA +GG+TT | -0.99 | | 1.67×${10}^{-3}$ | 0.139 |
|  | rs1065407+rs30187+rs10050860+rs27044+rs28096+rs13167972 | 33 | GT+TT +TT+CC+AA+GG  TT+CT +CT+GG+AG+AA | 1.23 | | 1.68×${10}^{-3}$ | 0 | NA | NA | | NA | 0.326 |
|  | rs1065407+rs2287987+rs26653+rs469876+rs17481856+rs17482078 | 15 | GG+CC +CC+GG+AA+CC  GT+CT+  GG+AA+  AG+CT | -0.33 | | 5.12×${10}^{-1}$ | 3 | TT+CT+ CG+AG+ AG+CT  GT+TT+GG+AA+AA +TT | -0.97 | | 1.74×${10}^{-3}$ | 0.020 |
|  | rs1065407+rs2287987+rs10050860+rs26653+rs469876+rs17481856 | 14 | GG+CC+ NA+CC +GG+AA  GT+TT+ NA+GG+ AA+AG | -0.48 | | 3.56×${10}^{-1}$ | 3 | TT+CT+NA+ CG+AG +AG  GT+TT+NA+GG+AA+AA | -0.96 | | 1.81×${10}^{-3}$ | 0.075 |
|  | rs1065407+rs30187+rs27044+rs28096+rs13167972+rs17482078 | 35 | GT+TT+ CC+AA+ GG+TT  TT+CT+ GG+AG+  AA+CT | 1.16 | | 2.07×${10}^{-3}$ | 0 | NA | NA | | NA | 0.227 |
|  | rs1065407+rs30187+rs27044+rs26653+rs17481856+rs13167972 | 29 | GG+TT+ CC+CC+ AA+AA  GT+CT+ GG+CG+ AG+AG | 0.80 | | 2.27×${10}^{-3}$ | 0 | NA | NA | | NA | 0.047 |
|  | rs1065407+rs2287987+rs27044+rs26653+rs469876+rs13167972 | 29 | GT+CC+ CC+CC+ AA+AA  TT+TT+ GG+GG+ AG+AG | 0.56 | | 1.23×${10}^{-1}$ | 3 | TT+CT+CG+CG+AG+AG  GT+TT+GG+GG+AA+AA | -0.92 | | 2.31×${10}^{-3}$ | 0.062 |
|  | rs1065407+rs2287987+rs30187+rs26653+rs469876+rs13167972 | 33 | GG+CC +CC+CC+  AA+AA  GT+TT +TT+CG+AG+AG | -0.04 | | 8.93×${10}^{-1}$ | 3 | TT+CT+CT+CG+AG+AG  GT+TT+TT+GG+AA+AA | -0.92 | | 2.41×${10}^{-3}$ | 0.226 |
|  | rs1065407+rs30187+rs27044+rs469876+rs17481856+rs13167972 | 22 | GG+TT+ CC+AA+ AA+AA  GT+CT+ GG+AG+ AG+AG | 1.52 | | 2.52×${10}^{-3}$ | 1 | TT+CT+ CG+AG+ AG+AG | -0.50 | | 8.75×${10}^{-2}$ | 0.171 |
|  | rs1065407+rs30187+rs10050860+rs17481856+rs28096+rs13167972 | 24 | GT+CC+ TT+AA+ AA+GG  TT+TT +CT+AG+AG+ AA | 1.52 | | 2.58×${10}^{-3}$ | 0 | NA | NA | | NA | 0.147 |
|  | rs1065407+rs27044+rs26653+rs27434+rs17481856+rs13167972 | 29 | TT+GG +CC+GG+AA+GG  GT+CG+ CG+AG+ AG+AA | 0.77 | | 2.62×${10}^{-3}$ | 0 | NA | NA | | NA | 0.072 |
|  | rs1065407+rs30187+rs17481856+rs28096+rs13167972+rs17482078 | 26 | GG+CC +AA+AA+GG+GG  GT+TT +AG+AG+AA+AA | 1.38 | | 3.17×${10}^{-3}$ | 1 | TT+CT+ AG+AG +AG+AG | -0.77 | | 9.99×${10}^{-2}$ | 0.175 |
|  | rs1065407+rs30187+rs27044+rs26653+rs27434+rs13167972 | 30 | GG+CC+ GG+CC+ GG+AA  GT+TT+ CG+CG+ AGAG | 0.76 | | 3.31×${10}^{-3}$ | 0 | NA | NA | | NA | 0.066 |
|  | rs10050860+rs27044+rs26653+rs27434+rs469876+rs17482078 | 13 | CT+CC+ CC+GG+ AA+TT  TT+GG+ CG+AG +AG+CT | 0.74 | | 3.52×${10}^{-3}$ | 0 | NA | NA | | NA | 0.002 |
|  | rs1065407+rs30187+rs27044+rs27434+rs469876+rs13167972 | 26 | GG+CC+ CC+GG+ AA+AA  GT+CT+ GG+AA+ AG+AG | 1.28 | | 3.54×${10}^{-3}$ | 1 | TT+CT+CG+AG+AG+AG | -0.55 | | 6.42×${10}^{-2}$ | 0.372 |
|  | rs1065407+rs10050860+rs27434+rs17481856+rs28096+rs13167972 | 24 | GT+TT +AA+AA+AA+GG  TT+CT+ AG+AG +AG +AA | 1.46 | | 3.88×${10}^{-3}$ | 1 | TT+CT+AG+AG+AG+AG | -0.77 | | 9.99×${10}^{-2}$ | 0.153 |
|  | rs1065407+rs10050860+rs27044+rs26653+rs469876+rs17481856 | 18 | GG+TT+ CC+CC +GG+AA  GT+CT+ GG+GG+ AA+AG | 0.77 | | 4.24×${10}^{-3}$ | 2 | TT+CT+CGCG+AG +AG  GT+TT+GG+GG+AA+AA | -0.97 | | 1.58×${10}^{-2}$ | 0.089 |
|  | rs1065407+rs27044+rs27434+rs17481856+rs28096+rs13167972 | 33 | TT+GG +AA+AA+AA+GG  GT+GG+ AG+AG +AG+AA | 0.72 | | 4.32×${10}^{-3}$ | 0 | NA | NA | | NA | 0.197 |
|  | rs1065407+rs2287987+rs27044+rs26653+rs27434+rs17482078 | 15 | GT+TT+ GG+CC+ GG+TT  GT+CT+ CG+CG+ AG+CT | 0.42 | | 2.43×${10}^{-1}$ | 2 | TT+CT+CG+GG+AA+CT  GT+TT+GG+CG+AG+ TT | -0.97 | | 4.37×${10}^{-3}$ | 0.022 |
|  | rs1065407+rs2287987+rs26653+rs27434+rs17481856+rs17482078 | 11 | GG+CT+ CC+GG+ AA+TT  GT+TT +CG+AG+AG+ CT | 0.32 | | 3.93×${10}^{-1}$ | 2 | TT+TT+GG+AA+AG+CT  GT+CT+CG+AG+AA +TT | -0.97 | | 4.37×${10}^{-3}$ | 0.026 |
|  | rs1065407+rs10050860+rs27044+rs26653+rs469876+rs17482078 | 18 | GT+CT +CC+CC+AA+TT  TT+TT +GG+CG+AG+CT | 0.77 | | 4.43×${10}^{-3}$ | 1 | TT+TT+ CG+CG +AG+CT | -0.77 | | 7.74×${10}^{-2}$ | 0.002 |
|  | rs1065407+rs30187+rs10050860+rs27434+rs28096+rs13167972 | 26 | GT+TT+ TT+GG +AA+GG  TT+CT +CT+AA  +AG+AA | 1.34 | | 4.4×${10}^{-3}$ | 0 | NA | NA | | NA | 0.381 |
|  | rs1065407+rs2287987+rs17481856+rs28096+rs13167972+rs17482078 | 19 | GT+TT+ AA+AA+ GG+TT  TT+CT+ AG+AG+ AA+CT | 0.84 | | 9.30×${10}^{-2}$ | 2 | TT+CT+ AG+AG+ AG+CT  GT+TT+ AA+AA+ AA+TT | -0.81 | | 4.52×${10}^{-3}$ | 0.023 |
|  | rs1065407+rs2287987+rs10050860+rs26653+rs27434+rs17481856 | 9 | GG+CC +NA+CC+GG+AA  GT+CC+ NA+CG +AG+AG | 0.26 | | 4.94×${10}^{-1}$ | 2 | TT+CT+ NA+GG +AA+AG  GT+TT+NA+CG+AG+AA | -0.96 | | 4.53×${10}^{-3}$ | 0.071 |
|  | rs1065407+rs2287987+rs10050860+rs27044+rs26653+rs27434 | 13 | GG+CC+ NA+GG+ CC+GG  GT+TT +NA+CG+CG+AG | 0.37 | | 3.11×${10}^{-1}$ | 2 | TT+CT+NA+CG+GG+AA  GT+TT+NA+GG+CG+AG | -0.96 | | 4.53×${10}^{-3}$ | 0.082 |
|  | rs1065407+rs2287987+rs30187+rs26653+rs27434+rs17482078 | 13 | GG+CT+ TT+CC+ GG+TT  GT+TT+ CT+CG +AG+CT | 0.31 | | 3.98×${10}^{-1}$ | 2 | TT+TT+ CT+GG+ AA+CT  GT+CT+ TT+CG+  AA+TT | -0.96 | | 4.53×${10}^{-3}$ | 0.015 |
|  | rs1065407+rs2287987+rs30187+rs26653+rs27434+rs17481856 | 9 | GG+CC+ TT+CC+ GG+GG  GT+TT+ CT+CG+ AG+AA | 0.50 | | 2.03×${10}^{-1}$ | 2 | TT+CT+CT+GG+AA+ AG  GT+TT+TT+CG+AG+AA | -0.96 | | 4.69×${10}^{-3}$ | 0.102 |
|  | rs1065407+rs2287987+rs30187+rs10050860+rs26653+rs27434 | 11 | CT+CC+ GG+GG+ NA+TT  TT+CG +AG+GT+NA+CT | 0.25 | | 4.96×${10}^{-1}$ | 2 | TT+GG+ AA+TT+ NA+CT  CT+CG+ AG+GT+ NA+TT | -0.96 | | 4.69×${10}^{-3}$ | 0.101 |
|  | rs1065407+rs2287987+rs10050860+rs17481856+rs28096+rs13167972 | 18 | GT+CC+ NA+AA+ AA+GG  TT+TT+ NA+AG +AG+ AA | 0.76 | | 1.33×${10}^{-1}$ | 2 | TT+CT+NA+AG+AG+ AG  GT+TT+NA+AA+AA+ AA | -0.80 | | 4.72×${10}^{-3}$ | 0.082 |
|  | rs1065407+rs10050860+rs27044+rs26653+rs17481856+rs17482078 | 13 | GG+CT+ CC+CC+ AA+TT  GT+TT+ GG+CG +AG+CT | 0.81 | | 4.75×${10}^{-3}$ | 1 | TT+TT+ CG+GG+ AG+CT | -0.84 | | 5.37×${10}^{-2}$ | 0.002 |
|  | rs1065407+rs27434+rs17481856+rs28096+rs13167972+rs17482078 | 26 | GT+AA+ AA+AA+ GG+TT  TT+AG+ AG+AG+ AG+CT | 1.33 | | 4.78×${10}^{-3}$ | 1 | TT+AG+ AG+AG+ AG+CT | -0.77 | | 9.84×${10}^{-2}$ | 0.162 |
|  | rs1065407+rs2287987+rs26653+rs27434+rs17481856+rs13167972 | 20 | GG+CC+ CC+GG+ AA+GG  GT+TT+ CG+ AG+  AG+AA | 0.42 | | 2.80×${10}^{-1}$ | 2 | TT+CT+GG+AA+AG+ AG  GT+TT+CG+AG+AA+ AA | -0.95 | | 4.85×${10}^{-3}$ | 0.077 |
|  | rs1065407+rs2287987+rs30187+rs26653+rs27434+rs13167972 | 22 | TT+CC+ CC+CC+ GG+GG  GT+TT+ CT+CG+ AG+AA | 0.40 | | 2.87×${10}^{-1}$ | 2 | TT+CT+CT+ GG+AA+ AG  GT+TT+TT+CG+AG+AA | -0.95 | | 5.02×${10}^{-3}$ | 0.086 |
|  | rs10050860+rs27044+rs26653+rs27434+rs469876+rs17481856 | 13 | TT+CC +CC+GG+AA+AA  CT+CG +GG+ AG+AG | 0.71 | | 5.09×${10}^{-3}$ | 0 | NA | NA | | NA | 0.136 |
|  | rs1065407+rs30187+rs27434+rs28096+rs13167972+rs17482078 | 28 | GT+TT+ GG+AA +GG+TT  TT+CT +AA+AG+ AA+ CT | 1.23 | | 5.49×${10}^{-3}$ | 0 | NA | NA | | NA | 0.266 |
|  | rs27044+rs26653+rs27434+rs469876+rs17481856+rs17482078 | 15 | CC+CC +GG+AA+AA+TT  GG+CG +AG+AG +AG+CT | 0.69 | | 5.80×${10}^{-3}$ | 0 | NA | NA | | NA | 0.104 |
|  | rs1065407+rs10050860+rs27044+rs26653+rs17481856+rs13167972 | 21 | GG+TT +CC+CC+AA+AA  GT+CT +GG+CG+AG+AG | 1.41 | | 5.82×${10}^{-3}$ | 1 | TT+CT+ CG+GG +AG+ AG | -0.83 | | 5.68×${10}^{-2}$ | 0.056 |
|  | rs1065407+rs26653+rs27434+rs17481856+rs13167972+rs17482078 | 21 | GG+CC +GG+AA+GG+TT  GT+CG +AG+AG+AA+CT | 0.60 | | 1.24×${10}^{-1}$ | 2 | TT+GG+ AA+AG +AG+AA  GT+CG+ AG+AA+ AA+AG | -0.91 | | 6.13×${10}^{-3}$ | 0.069 |
|  | rs1065407+rs10050860+rs26653+rs27434+rs17481856+rs13167972 | 19 | GG+TT +CC+GG+AA+GG  GT+CT+ CG+AG +AG+AA | 0.64 | | 1.12×${10}^{-1}$ | 2 | TT+CT+GG+AA+AG+AG  GT+TT+CG+AG+AA+AA | -0.90 | | 6.34×${10}^{-3}$ | 0.090 |
|  | rs1065407+rs2287987+rs27044+rs26653+rs27434+rs17481856 | 13 | GG+CC+ CC+CC+ GG+AA  GT+TT+ GG+CG +AG+AA | 0.44 | | 2.29×${10}^{-1}$ | 2 | TT+CT+ CG+GG+ AA+AG  GT+TT+ GG+CG+AG+AA | -0.93 | | 6.41×${10}^{-3}$ | 0.084 |
|  | rs10050860+rs27044+rs26653+rs469876+rs17481856+rs17482078 | 14 | CT+CC+ CC+AA+ AA+TT    TT+GG +GG+AG+  AG+CT | 0.67 | | 6.61×${10}^{-3}$ | 0 | NA | NA | | NA | 0.002 |
|  | rs1065407+rs2287987+rs30187+rs27044+rs26653+rs27434 | 14 | GG+CC+ TT+GG+ CG+GG  GT+CT+ CT+CG+ CG+AG | 0.50 | | 1.69×${10}^{-1}$ | 2 | TT+TT+ CT+CG+ GG+AA  GT+CT+ TT+GG+CG+AG | -0.92 | | 6.63×${10}^{-3}$ | 0.090 |
|  | rs1065407+rs30187+rs27434+rs17481856+rs28096+rs13167972 | 18 | GG+TT+ GG+GG+ AA+GG  GT+CT+ AG+AA+ AG+AA | 1.52 | | 6.65×${10}^{-3}$ | 0 | NA | NA | | NA | 0.170 |
|  | rs30187+rs27044+rs26653+rs27434+rs17481856+rs13167972 | 17 | TT+GG+ CC+GG +GG+GG  CT+CG+ CG+AG+ AA+AG | 0.81 | | 6.69×${10}^{-3}$ | 0 | NA | NA | | NA | 0.099 |
|  | rs1065407+rs2287987+rs27044+rs26653+rs27434+rs13167972 | 31 | GG+CC+ GG+CC+ GG+AA  GT+TT+ CG+CG +AG+AG | 0.42 | | 1.98×${10}^{-1}$ | 2 | TT+CT+CG+GG+AA+AG  GT+TT+GG+CG+AG+AA | -0.92 | | 6.86×${10}^{-3}$ | 0.078 |
|  | rs1065407+rs2287987+rs10050860+rs26653+rs17481856+rs17482078 | 7 | GG+CC+ NA+CC+ AA+TT  GT+TT+ NA+GG+ AG+AG | -0.89 | | 2.89×${10}^{-1}$ | 2 | TT+CT+ NA+ CG+AG+ AG  GT+TT+NA+GG+AA+AA | -0.89 | | 7.01×${10}^{-3}$ | 0.022 |
|  | rs1065407+rs2287987+rs10050860+rs27044+rs26653+rs17482078 | 12 | GG+CC+ NA+GG +CC+ TT  GT+TT+ NA+CG+ GG+CT | 0.69 | | 2.78×${10}^{-2}$ | 2 | TT+CT+ NA+CG +CG+ CT  GT+TT+NA+GG+GG+ TT | -0.89 | | 7.01×${10}^{-3}$ | 0.037 |
|  | rs1065407+rs27044+rs26653+rs17481856+rs13167972+rs17482078 | 23 | GG+CC +CC+AA+AA+TT  GT+GG+ CG+AG+ AG+CT | 1.27 | | 7.20×${10}^{-3}$ | 1 | TT+CG+GG+AG+AG+ CT  GT+GG+CG+AA+AA+TT | -0.83 | | 5.58×${10}^{-2}$ | 0.036 |
|  | rs1065407+rs2287987+rs30187+rs26653+rs17481856+rs17482078 | 12 | GG+CT+ CC+CC +AA+TT  GT+TT +CG+CG+AA+TT | 0.38 | | 3.05×${10}^{-1}$ | 2 | TT+TT+TT+GG+AG+CT  GT+CT+CT+CG+AA+ TT | -0.89 | | 7.25×${10}^{-3}$ | 0.021 |
|  | rs1065407+rs2287987+rs30187+rs10050860+rs26653+rs17482078 | 12 | GG+CC +CC+NA+CC+TT  GT+TT +TT+NA+CG+CT | 0.25 | | 5.09×${10}^{-1}$ | 2 | TT+CT+ CT+NA+ GG+CT  GT+TT+TT+NA+CG +TT | -0.89 | | 7.25×${10}^{-3}$ | 0.014 |
|  | rs1065407+rs2287987+rs10050860+rs469876+rs17481856+rs17482078 | 8 | GG+CC +NA+AA+AA+TT  GT+TT+ NA+AG+AG+CT | -1.07 | | 1.91×${10}^{-1}$ | 2 | TT+CT+NA+AG+AG+CT  GT+TT+NA+AA+AA+ TT | -0.89 | | 7.25×${10}^{-3}$ | 0.015 |
|  | rs1065407+rs2287987+rs30187+rs10050860+rs26653+rs17481856 | 10 | GG+TT+ CC+NA+ CC+AA  GT+TT+ CT+NA+CG+AG | 0.33 | | 3.88×${10}^{-1}$ | 2 | TT+CT+TT+NA+GG+AG  GT+TT+CT+NA+CG+AA | -0.88 | | 7.50×${10}^{-3}$ | 0.087 |
|  | rs1065407+rs2287987+rs26653+rs17481856+rs13167972+rs17482078 | 17 | GG+TT+ CC+AA+ GG+TT  GT+CT+ GG+AG+  AA+CT | 0.92 | | 6.36×${10}^{-2}$ | 2 | TT+CT+ CG+AG+ AG+CT  GT+TT+GG+AA+AA+ TT | -0.88 | | 7.50×${10}^{-3}$ | 0.015 |
|  | rs1065407+rs2287987+rs10050860+rs26653+rs17481856+rs13167972 | 17 | GG+CC +NA+CC+AA+GG  GT+TT +NA+GG+AG+CT | 0.93 | | 1.69×${10}^{-2}$ | 2 | TT+CT+NA+CG+AG +CT  GT+TT+NA+GG+AA+ TT | -0.88 | | 7.76×${10}^{-3}$ | 0.070 |
|  | rs1065407+rs2287987+rs30187+rs26653+rs13167972+rs17482078 | 24 | GG+TT +CC+CC+GG+TT  GT+CT +TT+CG+AA+CT | 0.24 | | 5.03×${10}^{-1}$ | 2 | TT+CT+CT+GG+AG+ TT  GT+TT+TT+CG+AA+ CT | -0.88 | | 7.76×${10}^{-3}$ | 0.028 |
|  | rs1065407+rs2287987+rs469876+rs17481856+rs13167972+rs17482078 | 20 | GG+TT +AA+AA+GG+TT  GT+CT+AG+AG+AA+CT | 0.51 | | 2.45×${10}^{-1}$ | 2 | TT+CT+ AG+AG+ AG+CT  GT+TT+AA+AA+AA+ TT | -0.88 | | 7.76×${10}^{-3}$ | 0.022 |
|  | rs1065407+rs10050860+rs27044+rs27434+rs469876+rs13167972 | 30 | GT+TT+ CC+AA+AA+AA  TT+CT+ GG+AG +AG+AG | 1.06 | | 7.83×${10}^{-3}$ | 1 | TT+CT+CG+ AG+AG +AG | -0.89 | | 5.03×${10}^{-2}$ | 0.161 |
|  | rs1065407+rs26653+rs469876+rs17481856+rs13167972+rs17482078 | 26 | GT+CC +GG+AA+GG+TT  TT+GG +AA+AG+AA+CT | 0.60 | | 1.24×${10}^{-1}$ | 2 | TT+CG+AG+AG+AG+CT  GT+GG +AA+AA+AA+TT | -1.06 | | 7.85×${10}^{-3}$ | 0.125 |
|  | rs2287987+rs27044+rs469876+rs17481856+rs28096+rs17482078 | 14 | TT+CC  +AA+AA+GG+TT  CT+GG+AG+AG+AG+CT | -0.27 | | 6.21×${10}^{-1}$ | 2 | CT+CG+ AG+AG +AA+CT  TT+GG +AA+AA+AG+TT | -1.17 | | 7.93×${10}^{-3}$ | 0.022 |
|  | rs2287987+rs10050860+rs469876+rs17481856+rs28096+rs17482078 | 10 | CC+NA+ AA+AA+AA+TT  TT+NA+AG+AG+AG+CT | -0.39 | | 5.51×${10}^{-1}$ | 2 | CT+NA+AG+AG+AG+CT  TT+NA+AA+AA+AA+TT | -1.17 | | 7.93×${10}^{-3}$ | 0.015 |
|  | rs1065407+rs2287987+rs30187+rs26653+rs17481856+rs13167972 | 21 | GG+CC +CC+CC+AA+GG  GT+TT +CT+CG+AG+AA | 0.48 | | 2.11×${10}^{-1}$ | 2 | TT+CT+TT+GG+AG+AG  GT+TT+TT+GG+AA+AA | -0.88 | | 8.02×${10}^{-3}$ | 0.083 |
|  | rs1065407+rs2287987+rs30187+rs10050860+rs26653+rs13167972 | 23 | GG+CC +CC+NA+CC+GG  GT+TT +TT+NA+CG+AA | 0.18 | | 6.14×${10}^{-1}$ | 2 | TT+CT+CT+NA+GG+AG  GT+TT+TT+NA+CG+AA | -0.88 | | 8.02×${10}^{-3}$ | 0.085 |
|  | rs1065407+rs2287987+rs27044+rs469876+rs17481856+rs13167972 | 22 | GT+CC +CC+AA+AA+AA  TT+TT +GG+AG+AG+AG | 0.84 | | 5.55×${10}^{-2}$ | 2 | GG+CT +CG+AG+AG+AG  GT+TT+GG+AA+AA+AA | -0.88 | | 8.03×${10}^{-3}$ | 0.096 |
|  | rs1065407+rs2287987+rs10050860+rs469876+rs17481856+rs13167972 | 20 | GT+CC +NA+AA+AA+GG  TT+TT +NA+AG+AG+AA | 0.66 | | 6.52×${10}^{-2}$ | 2 | TT+CT+NA+AG+AG+AG  GT+TT+NA+AA+AA+AA | -0.88 | | 8.03×${10}^{-3}$ | 0.050 |
|  | rs1065407+rs10050860+rs26653+rs469876+rs17481856+rs13167972 | 24 | GT+TT +CC+GG+AA+GG  TT+CT +GG+AA+AG+AA | 0.64 | | 1.12×${10}^{-1}$ | 2 | TT+CT+ CG+AG+ AG+AG  GT+TT+ GG+AA +AA+AA | -1.05 | | 8.07×${10}^{-3}$ | 0.139 |
|  |  |  |  |  | |  |  |  |  | |  |  |
|  |  |  |  |  |  |  |  |  |  |  |  |  |
|  |  |  |  |  |  |  |  |  |  |  |  |  |
|  |  |  |  |  |  |  |  |  |  |  |  |  |
|  |  |  |  |  |  |  |  |  |  |  |  |  |
|  |  |  |  |  |  |  |  |  |  |  |  |  |
|  |  |  |  |  |  |  |  |  |  |  |  |  |
|  |  |  |  |  |  |  |  |  |  |  |  |  |
|  |  |  |  |  |  |  |  |  |  |  |  |  |
|  |  |  |  |  |  |  |  |  |  |  |  |  |
|  |  |  |  |  |  |  |  |  |  |  |  |  |
|  |  |  |  |  |  |  |  |  |  |  |  |  |
| #7Interaction Effects | rs1065407+rs2287987+rs26653+rs469876+rs17481856+rs13167972+rs17482078 | 28 | GG+TT+ CC+AA+ AA+GG+ TT  GT+CT+ GG+AG +AG+AA+ CT | | 0.31 | 3.92×${10}^{-1}$ | 3 | TT+CT+ CG+AG+ AG+ AG+ CT  GT+TT+ GG+ AA+ AA+ AA+ TT | | -1.00 | 1.60×${10}^{-3}$ | 0.020 |
|  | rs1065407+rs2287987+rs10050860+rs26653+rs469876+rs17481856+rs13167972 | 28 | GT+CC+ NA+CC+ GG+AA+ GG  TT+TT+ NA+GG+ AA+AG+ AA | | 0.49 | 1.20×${10}^{-1}$ | 3 | TT+CT+ NA+CG+ AG+AG+ AG  GT+TT+NA+GG+AA+ AA+AA | | -0.99 | 1.67×${10}^{-3}$ | 0.063 |
|  | rs1065407+rs2287987+rs10050860+rs26653+rs469876+rs17481856+rs17482078 | 16 | GG+CC+ NA+CC+ GG+AA+ TT  GT+TT+ NA+GG+ AA+AG+ CT | | -0.43 | 3.84×${10}^{-1}$ | 3 | TT+CT+ NA+CG+ AG+AG+ CT  GT+TT+ NA+GG +AA+AA+ TT | | -0.97 | 1.74×${10}^{-3}$ | 0.014 |
|  | rs1065407+rs2287987+rs10050860+rs27044+rs26653+rs469876+rs13167972 | 32 | GT+CC+ NA+CC+ CC+AA+ TT  TT+TT +NA+GG+GG+AG  +CT | | 0.59 | 5.31×${10}^{-2}$ | 3 | TT+CT+ NA+CG +CG+AG+ CT  GT+TT+ NA+ GG+ GG+AA+ TT | | -0.92 | 2.31×${10}^{-3}$ | 0.053 |
|  | rs1065407+rs2287987+rs30187+rs27044+rs26653+rs469876+rs13167972 | 45 | GT+CC +CC+CC+CC+AA +AA  TT+TT +CT+GG+GG+AG+ AG | | 0.14 | 6.27×${10}^{-1}$ | 3 | TT+CT+ TT+CG+CG+ AG+AG  GT+TT+CT+GG+GG+AA+AA | | -0.92 | 2.41×${10}^{-3}$ | 0.237 |
|  | rs1065407+rs2287987+rs30187+rs10050860+rs26653+rs469876+rs13167972 | 36 | GG+CC +CC+NA+ CC+AA+ AA  GT+TT +TT+NA+CG+AG+ AG | | -0.13 | 6.67×${10}^{-1}$ | 3 | TT+CT+CT+NA+GG+AG+ AG  GT+TT+TT+NA+ CG+AA+ AA | | -0.92 | 2.41×${10}^{-3}$ | 0.097 |
|  | rs1065407+rs30187+rs27044+rs26653+rs27434+rs17481856+rs13167972 | 30 | GG+TT+ GG+CC+ GG+GG+ AA  GT+CT+ CG+CG+ AG+AA+ AG | | 0.76 | 3.31×${10}^{-3}$ | 0 | NA | | NA | NA | 0.101 |
|  | rs1065407+rs30187+rs27044+rs27434+rs469876+rs17481856+rs13167972 | 26 | GG+TT+ CC+GG+ AA+GG+ AA  GT+CT+ GG+AG+ AG+ AA+ AG | | 1.28 | 3.54×${10}^{-3}$ | 1 | TT+CT+ CG+AA+ AG+AG+ AG | | -0.55 | 6.42×${10}^{-2}$ | 0.121 |
|  | rs1065407+rs30187+rs10050860+rs27044+rs28096+rs13167972+rs17482078 | 36 | GT+TT +CT+CC+AA+GG+ TT  TT+CT+ TT+GG+ AG+AA+ CT | | 1.06 | 3.59×${10}^{-3}$ | 0 | NA | | NA | NA | 0.002 |
|  | rs1065407+rs2287987+rs10050860+rs27044+rs26653+rs27434+rs17482078 | 16 | GG+TT +NA+GG+CC+GG +TT  GT+CT +NA+CG+CG+AG +CT | | 0.35 | 3.25×${10}^{-1}$ | 2 | TT+CT+ NA+CG+ GG+AA +CT  GT+TT+NA+GG+CG+AG+TT | | -0.97 | 4.37×${10}^{-3}$ | 0.012 |
|  | rs1065407+rs2287987+rs10050860+rs26653+rs27434+rs17481856+rs17482078 | 12 | GG+CC+ NA+CC+ GG+AA+ TT  GT+TT+ NA+CG+ AG+AG+ CT | | 0.25 | 5.09×${10}^{-1}$ | 2 | TT+CT+ NA+GG+ AA+AG+ CT  GT+TT+NA+CG+AG+AA+TT | | -0.97 | 4.37×${10}^{-3}$ | 0.010 |
|  | rs1065407+rs30187+rs10050860+rs27434+rs17481856+rs28096+rs13167972 | 26 | GT+TT+ TT+GG+ GG+AA+ GG  TT+CT+ CT+AG+ AA+AG+ AA | | 1.34 | 4.45×${10}^{-3}$ | 0 | NA | | NA | NA | 0.140 |
|  | rs1065407+rs2287987+rs10050860+rs17481856+rs28096+rs13167972+rs17482078 | 20 | GT+CC+ NA+ AA+ AA+GG+ TT  TT+TT+ NA+ AG+ AG+AA+ CT | | 0.68 | 1.51×${10}^{-1}$ | 2 | TT+CT+ NA+ AG+ AG+AG+ CT  GT+TT+NA+AA+AA+ AA+TT | | -0.81 | 4.52×${10}^{-3}$ | 0.026 |
|  | rs1065407+rs2287987+rs30187+rs26653+rs27434+rs17481856+rs17482078 | 13 | GG+CT+ TT+CC+ GG+GG+ TT  GT+TT+ CT+CG+ AG+AA+ CT | | 0.31 | 3.98×${10}^{-1}$ | 2 | TT+TT+ CT+GG+ AA+AG+ CT  GT+CT+ TT+CG+AG+AA+TT | | -0.96 | 4.53×${10}^{-3}$ | 0.015 |
|  | rs1065407+rs2287987+rs30187+rs10050860+rs26653+rs27434+rs17482078 | 14 | GG+CC +TT+NA+CC+ GG+ TT  GT+TT+ CT+NA+ CG+ AG+ CT | | 0.24 | 5.10×${10}^{-1}$ | 2 | TT+CT+ CT+NA+ GG+AA+ CT  GT+TT+ TT+NA+ CG+AG+ TT | | -0.96 | 4.53×${10}^{-3}$ | 0.008 |
|  | rs1065407+rs2287987+rs30187+rs10050860+rs26653+rs27434+rs17481856 | 11 | GG+CC+ TT+NA+ CC+GG+ GG  GT+TT+ CT+NA+ CG+AG+ AA | | 0.25 | 4.96×${10}^{-1}$ | 2 | TT+CT+ CT+ NA+ GG+AA+ AG  GT+TT+ TT+NA+ CG+AG+ AA | | -0.96 | 4.69×${10}^{-3}$ | 0.065 |
|  | rs1065407+rs2287987+rs26653+rs27434+rs17481856+rs13167972+rs17482078 | 24 | GG+CC+ TT+NA+ CC+GG+ GG  GT+TT+ CT+NA+ CG+AG+ AA | | 0.24 | 5.03×${10}^{-1}$ | 2 | TT+CT+ CT+NA+ GG+AA+ AG  GT+TT+TT+NA+CG+AG+AA | | -0.96 | 4.69×${10}^{-3}$ | 0.065 |
|  | rs1065407+rs2287987+rs10050860+rs26653+rs27434+rs17481856+rs13167972 | 23 | GG+CC+ NA+CC+ GG+ AA+ GG  GT+TT +NA+CG+AG+AG+ TT | | 0.18 | 6.14×${10}^{-1}$ | 2 | TT+CT+ NA+GG+ AA+AG+ AG  GT+TT+NA+CG+AG+AA+AA | | -0.95 | 4.85×${10}^{-3}$ | 0.082 |
|  | rs1065407+rs2287987+rs30187+rs26653+rs27434+rs13167972+rs17482078 | 26 | GG+CT+ TT+CC+ GG+GG+ TT  GT+TT+ CT+CG+  AG+AA+ CT | | 0.23 | 5.03×${10}^{-1}$ | 2 | TT+TT+ CT+GG+ AA+AG+ CT  GT+CT+ TT+CG+AG+AA+TT | | -0.95 | 4.85×${10}^{-3}$ | 0.021 |
|  | rs1065407+rs2287987+rs30187+rs26653+rs27434+rs17481856+rs13167972 | 22 | GG+CC+ TT+CC+ GG+GG+ GG  GT+TT+ CT+CG+ AG+AA+ AA | | 0.40 | 2.87×${10}^{-1}$ | 2 | TT+CT+ CT+GG+ AA+AG+ AG  GT+TT+TT+CG+AG+AA+ AA | | -0.95 | 5.02×${10}^{-3}$ | 0.097 |
|  | rs1065407+rs2287987+rs30187+rs10050860+rs26653+rs27434+rs13167972 | 25 | GG+CC+ TT+CC+ GG+GG+ GG  GT+TT+ CT+CG+ AG+AA+ AA | | 0.18 | 6.11×${10}^{-1}$ | 2 | TT+CT+ CT+GG+ AA+AG+ AG  GT+TT+TT+ CG+AG+ AA+AA | | -0.95 | 5.02×${10}^{-3}$ | 0.098 |
|  | rs1065407+rs30187+rs27434+rs17481856+rs28096+rs13167972+rs17482078 | 28 | GT+TT+ GG+GG+ AA+GG+ TT  TT+CT+ AG+AA+ AG+AA+ CT | | 1.23 | 5.49×${10}^{-3}$ | 0 | NA | | NA | NA | 0.131 |
|  | rs1065407+rs30187+rs10050860+rs17481856+rs28096+rs13167972+rs17482078 | 27 | GT+CC+ CT+AA+ AA+GG+ TT  TT+TT+TT+AG+AG+ AA+CT | | 1.22 | 5.63×${10}^{-3}$ | 1 | TT+CT+ TT+AG+ AG+AG+ CT | | -0.77 | 9.99×${10}^{-2}$ | 0.002 |
|  | rs1065407+rs10050860+rs26653+rs27434+rs17481856+rs13167972+rs17482078 | 22 | GG+CT +CC+GG+ AA+GG+ TT  GT+TT+ CG+AG+ AG+AA+ CT | | 0.51 | 1.81×${10}^{-1}$ | 2 | TT+TT+GG+AA+AG+AG+CT  GT+CT+CG+AG+AA+AA+TT | | -0.91 | 6.13×${10}^{-3}$ | 0.002 |
|  | rs1065407+rs2287987+rs27044+rs26653+rs27434+rs17481856+rs17482078 | 17 | GG+TT+ CC+CC +GG+AA+TT  GT+CT+ GG+CG +AG+CT | | 0.28 | 4.22×${10}^{-1}$ | 2 | TT+CT+ CG+GG +AA+AG+CT  GT+TT+GG+CG+AG+AA+TT | | -0.93 | 6.21×${10}^{-3}$ | 0.016 |
|  | rs1065407+rs2287987+rs10050860+rs27044+rs26653+rs27434+rs17481856 | 15 | GG+CC +NA+GG+CC+GG+ AA  GT+TT+ NA+CG+ CG+AG+ AG | | 0.23 | 5.17×${10}^{-1}$ | 2 | TT+CT+ NA+CG+ GG+AA+ AG  GT+TT+ NA+GG+ CG+AG+ AA | | -0.93 | 6.41×${10}^{-3}$ | 0.055 |
|  | rs1065407+rs2287987+rs30187+rs27044+rs26653+rs27434+rs17482078 | 18 | GG+TT+ CT+GG +CC+GG+TT  GT+CT+ TT+CG+ CG+AG+ AG | | 0.33 | 3.29×${10}^{-1}$ | 2 | TT+CT +TT+CG+ GG+AA+ CT  GT+TT+ CT+GG+ CG+AG+ TT | | -0.93 | 6.42×${10}^{-3}$ | 0.015 |
|  | rs1065407+rs2287987+rs30187+rs27044+rs26653+rs27434+rs17481856 | 14 | GG+CC+TT+GG+CC+GG+GG  GT+TT+ CT+CG+ CG+AG+ AA | | 0.50 | 1.69×${10}^{-1}$ | 2 | TT+CT+ CT+CG+ GG+AA+ AG  GT+TT+ TT+GG+ CG+AG+ AA | | -0.92 | 6.63×${10}^{-3}$ | 0.103 |
|  | rs1065407+rs2287987+rs30187+rs10050860+rs27044+rs26653+rs27434 | 16 | GG+CC+ TT+NA +GG+CC+ GG  GT+TT+ CT+NA+ CG+CG+ AG | | 0.29 | 4.09×${10}^{-1}$ | 2 | TT+CT+ CT+NA+ CG+ GG+  AA  GT+TT+TT+NA+GG+CG+AG | | -0.92 | 6.63×${10}^{-3}$ | 0.069 |
|  | rs1065407+rs2287987+rs27044+rs26653+rs27434+rs13167972+rs17482078 | 35 | GG+TT +GG+CC+GG+AA+ TT  GT+CT+ CG+CG+ AG+AG+ CT | | 0.35 | 2.75×${10}^{-1}$ | 2 | TT+CT+ CG+GG+ AA+AG+ CT  GT+TT+ GG+CG+AG+AA+TT | | -0.92 | 6.63×${10}^{-3}$ | 0.022 |
|  | rs1065407+rs2287987+rs27044+rs26653+rs27434+rs17481856+rs13167972 | 34 | GG+CC +CC+CC+ GG+AA +AA  GT+TT+ GG+CG+ AG+AG+ AG | | 0.35 | 2.70×${10}^{-1}$ | 2 | TT+CT+ CG+ GG+AA +AG+AG  GT+TT+GG+ CG+AG+ AA+AA | | -0.92 | 6.86×${10}^{-3}$ | 0.108 |
|  | rs1065407+rs2287987+rs10050860+rs27044+rs26653+rs27434+rs13167972 | 34 | GG+CC+ NA+GG+ CC+GG+ AA  GT+TT+ NA+CG +CG+AG +AG | | 0.30 | 3.40×${10}^{-1}$ | 2 | TT+CT+ NA+CG +GG+AG+ AG  GT+TT+NA+GG+CG+ AA+AA | | -0.92 | 6.86×${10}^{-3}$ | 0.077 |
|  | rs1065407+rs2287987+rs30187+rs27044+rs26653+rs27434+rs13167972 | 35 | GG+CC+ TT+CC +CC+GG+ AA  GT+TT+ CT+GG+ CG+AG+ AG | | 0.40 | 2.07×${10}^{-1}$ | 2 | TT+CT+CT+CG+ GG+ AA+AG  GT+TT+TT+GG+CG+AG+AA | | -0.92 | 7.09×${10}^{-3}$ | 0.081 |
|  | rs1065407+rs2287987+rs30187+rs10050860+rs26653+rs17481856+rs17482078 | 13 | GG+CC+ CC+NA +CC+AA+ TT  GT+TT+ CT+NA +CG+AG+ CT | | 0.31 | 4.04×${10}^{-1}$ | 2 | TT+CT+TT+NA+GG+AG+CT  GT+TT +CT+NA +CG+AA +TT | | -0.89 | 7.25×${10}^{-3}$ | 0.019 |
|  | rs1065407+rs10050860+rs27044+rs26653+rs469876+rs17481856+rs17482078 | 21 | GG+CT+ CC+CC +AA+AA +TT  GT+TT+ GG+AG+ AG+AG +CT | | 0.71 | 7.38×${10}^{-3}$ | 2 | TT+TT+ CG+CG+ AG+AG+ CT  GT+CT+GG+GG+AA+AA+TT | | -0.97 | 1.54×${10}^{-2}$ | 0.002 |
|  | rs1065407+rs2287987+rs10050860+rs26653+rs17481856+rs13167972+rs17482078 | 18 | GG+CC+ NA+CC+ AA+GG+ TT  TT+CT+ NA+CG +AG+AA +CT | | 0.76 | 1.06×${10}^{-1}$ | 2 | TT+CT+NA+CG+AG+ AG+CT  GT+TT+NA+GG+AA+ AA+TT | | -0.88 | 7.50×${10}^{-3}$ | 0.008 |
|  | rs1065407+rs2287987+rs30187+rs26653+rs17481856+rs13167972+rs17482078 | 25 | GG+CT+ CC+CC+ AA+GG+ TT  TT+CT+ CT+CG +AG+AA+ CT | | 0.30 | 4.01×${10}^{-1}$ | 2 | TT+TT+ TT+GG +AG+AG+CT  GT+CT+CT+CG+AA+AA+TT | | -0.88 | 7.76×${10}^{-3}$ | 0.035 |
|  | rs1065407+rs2287987+rs30187+rs10050860+rs26653+rs13167972+rs17482078 | 25 | GG+TT+ CC+NA+ GG+AG+ TT  GT+CT +TT+NA+CG+AA+ CT | | 0.17 | 6.25×${10}^{-1}$ | 2 | TT+CT+ CT+NA+ GG+AG +CT  GT+TT+TT+NA+CG+ AA+TT | | -0.88 | 7.76×${10}^{-3}$ | 0.021 |
|  | rs1065407+rs2287987+rs10050860+rs469876+rs17481856+rs13167972+rs17482078 | 22 | GT+CC +NA+AA+AA+GG +TT  TT+TT +NA+AG+AG+AA +CT | | 0.62 | 7.21×${10}^{-2}$ | 2 | TT+CT+ NA+AG +AG+ AG+CT  GT+TT+NA+AA+AA+AA+TT | | -0.88 | 7.76×${10}^{-3}$ | 0.012 |
|  | rs1065407+rs10050860+rs26653+rs469876+rs17481856+rs13167972+rs17482078 | 27 | GT+CT+ CC+GG+ AA+ GG+ TT  TT+TT+ GG+AA +AG+AA+CT | | 0.51 | 1.81×${10}^{-1}$ | 2 | TT+TT+ CG+AG +AG+AG+CT  GT+CT+GG+AA+AA+AA+TT | | -1.06 | 7.85×${10}^{-3}$ | 0.002 |
|  | rs2287987+rs10050860+rs27044+rs469876+rs17481856+rs28096+rs17482078 | 15 | CC+NA+ CC+AA+ AA+GG+ TT    TT+NA+ GG+AG+ AG+AA+ CT | | -0.39 | 4.63×${10}^{-1}$ | 2 | CT+NA+ GG+AG+ AG+AA+ CT  TT+NA+ CG+AA+ AA+AG+ TT | | -1.17 | 7.93×${10}^{-3}$ | 0.014 |
|  | rs1065407+rs2287987+rs30187+rs10050860+rs26653+rs17481856+rs13167972 | 24 | GG+CC +CC+NA+CC+AA+ GG  GT+TT+ CT+NA+ CG+AG +AA | | 0.24 | 4.97×${10}^{-1}$ | 2 | TT+CT+ TT+NA+ GG+AG+ AG  GT+TT+CT+NA+CG+AA+AA | | -0.88 | 8.02×${10}^{-3}$ | 0.069 |
|  | rs1065407+rs10050860+rs27434+rs17481856+rs28096+rs13167972+rs17482078 | 27 | GT+CT+ AA+AA+ AA+GG+ TT  TT+TT+ AG+AG +AG+AA+CT | | 1.17 | 8.49×${10}^{-3}$ | 1 | TT+TT+AG+ AG+AG+ AG+CT | | -0.77 | 9.84×${10}^{-2}$ | 0.002 |
|  | rs10050860+rs27044+rs26653+rs27434+rs469876+rs17481856+rs17482078 | 16 | CT+CC+ CC+GG+ AA+AA+ TT  TT+GG+ CG+AG+ AG+AG+ CT | | 0.65 | 8.5×${10}^{-3}$ | 0 | NA | | NA | NA | 0.002 |
|  | rs1065407+rs2287987+rs26653+rs27434+rs469876+rs17481856+rs17482078 | 22 | GG+CT+ CC+GG +GG+ AA +TT  GT+TT+ CG+AG+ AA+AG+ CT | | 0.10 | 7.64×${10}^{-1}$ | 2 | TT+TT+GG+AA+AG+ AG+CT | | -0.89 | 9.05×${10}^{-3}$ | 0.018 |
|  | rs1065407+rs2287987+rs27044+rs27434+rs469876+rs17481856+rs17482078 | 15 | GG+TT+ CC+AA+ AA+AA+ TT  GT+CT+ GG+AA +AA+AA +TT | | -0.67 | 2.28×${10}^{-1}$ | 2 | TT+CT+ CG+AG +AG+AG+CT  GT+TT+GG+AA+AA+AA+TT | | -0.89 | 9.05×${10}^{-3}$ | 0.016 |
|  | rs1065407+rs2287987+rs10050860+rs27434+rs469876+rs17481856+rs17482078 | 10 | GG+CC+ NA+GG+ AA+AA +TT  GT+TT+ NA+AG+ AG+AG+ CT | | -1.37 | 8.58×${10}^{-2}$ | 2 | TT+CT+NA+AG+AG+AG+CT  GT+TT+NA+AA+AA+ AA+TT | | -0.89 | 9.05×${10}^{-3}$ | 0.015 |
|  | rs1065407+rs2287987+rs27044+rs26653+rs27434+rs469876+rs17482078 | 25 | GG+TT+ GG+CC+ GG+AA+ TT  GT+CT+ CG+CG+ AG+AG+ CT | | 0.23 | 4.47×${10}^{-1}$ | 2 | TT+CT+ CG+GG +AA+AG+CT  GT+TT+GG+GG+AG+ AA+TT | | -0.89 | 9.05×${10}^{-3}$ | 0.025 |
|  | rs1065407+rs2287987+rs10050860+rs27044+rs27434+rs469876+rs17482078 | 14 | GT+CC+ NA+CC+ AA+AA+ TT  TT+TT+ NA+GG+ AG+AG+ CT | | -0.56 | 3.21×${10}^{-1}$ | 2 | TT+CT+ NA+CG +AG+AG+CT  GT+TT+NA+ GG+AA +AA+TT | | -0.89 | 9.05×${10}^{-3}$ | 0.018 |
|  | rs1065407+rs27044+rs26653+rs27434+rs469876+rs17481856+rs17482078 | 24 | GG+CC+ CC+GG+ GG+AA+ TT  GT+GG+ CG+AG+ AA+AG+ CT | | 0.24 | 4.69×${10}^{-1}$ | 2 | TT+CG+ GG+AA +AG+AG+CT  GT+GG+CG+AG+AA+AA+TT | | -1.09 | 9.18×${10}^{-3}$ | 0.106 |
|  | rs1065407+rs2287987+rs27044+rs26653+rs27434+rs469876+rs17481856 | 24 | GG+CC+ CC+CC+ GG+AA+ AA  GT+TT+ GG+CG+ AG+AG+ AG | | 0.19 | 5.34×${10}^{-1}$ | 2 | TT+CT+ CG+GG+ AA+AG+ AG  GT+TT+GG+CG+AG+AA+AA | | -0.89 | 9.35×${10}^{-3}$ | 0.178 |
|  | rs1065407+rs2287987+rs10050860+rs26653+rs27434+rs469876+rs17481856 | 21 | GG+CC+NA+CC+GG+ GG+AA  GT+TT+NA+CG+AG+ AA+AG | | 0.05 | 8.80×${10}^{-1}$ | 2 | TT+CT+NA+CG+GG+AG+ AG  GT+TT+NA+GG+CG+AA+AA | | -0.89 | 9.35×${10}^{-3}$ | 0.085 |
|  | rs1065407+rs2287987+rs10050860+rs27044+rs27434+rs469876+rs17481856 | 14 | GT+CC+NA+CC+AA+ AA+AA  TT+TT+NA+GG+AG+ AG+AG | | -0.89 | 1.36×${10}^{-1}$ | 2 | TT+CT+NA+CG+AG+ AG+AG  GT+TT+NA+GG+AA+ AA+AA | | -0.89 | 9.35×${10}^{-3}$ | 0.069 |
|  | rs1065407+rs2287987+rs10050860+rs27044+rs26653+rs27434+rs469876 | 24 | GG+CC+NA+GG+CC+ GG+AA  TT+CT+NA+CG+CG+ AG+AG | | 0.19 | 5.34×${10}^{-1}$ | 2 | TT+CT+NA+CG+GG+AA+AG  GT+TT+NA+GG+CG+ AG+AA | | -0.89 | 9.35×${10}^{-3}$ | 0.057 |
|  | rs1065407+rs2287987+rs30187+rs27434+rs469876+rs17481856+rs17482078 | 12 | GG+CT+TT+GG+AA +GG+TT  GT+TT+CT+AG+AG+ AA+TT | | -1.07 | 1.10×${10}^{-1}$ | 2 | TT+TT+CT+AA+AG+ AG+CT  GT+CT+TT+AG+AA+AA+TT | | -0.89 | 9.35×${10}^{-3}$ | 0.015 |
|  | rs1065407+rs2287987+rs30187+rs26653+rs27434+rs469876+rs17482078 | 24 | GG+CT+TT+CC+GG+ GG+TT  GT+TT+CT+CG+AG +AA+CT | | 0.10 | 7.55×${10}^{-1}$ | 2 | TT+TT+CT+GG+AA+ AG+CT  GT+CT+TT+CG+AG+AA+TT | | -0.89 | 9.35×${10}^{-3}$ | 0.026 |
|  | rs1065407+rs2287987+rs30187+rs27044+rs27434+rs469876+rs17482078 | 17 | GG+TT+CT+CC+AA +AA+TT  GT+CT+TT+GG+AG+ AG+CT | | -0.58 | 2.62×${10}^{-1}$ | 2 | TT+CT+TT+GG+AG+ AG+CT  GT+TT+CT+CG+AA+AA+TT | | -0.89 | 9.35×${10}^{-3}$ | 0.026 |
|  | rs1065407+rs2287987+rs30187+rs10050860+rs27434+rs469876+rs17482078 | 13 | GG+CC+CC+NA+AA+ AA+TT  GT+TT+CT+NA+AG+ AG+CT | | -1.18 | 7.47×${10}^{-2}$ | 2 | TT+CT+TT+NA+AG+AG+CT  GT+TT+CT+NA+AA+AA+TT | | -0.89 | 9.35×${10}^{-3}$ | 0.006 |
|  | rs1065407+rs10050860+rs27044+rs26653+rs27434+rs469876+rs17481856 | 22 | GG+TT+CC+CC+GG+ GG+AA  GT+CT+GG+CG+AG+ AA+AG | | 0.26 | 4.54×${10}^{-1}$ | 2 | TT+CT+CG+GG+AA+AG+AG  GT+TT+GG+CG+AG+AA+AA | | -1.09 | 9.41×${10}^{-3}$ | 0.174 |
|  | rs1065407+rs26653+rs27434+rs469876+rs17481856+rs13167972+rs17482078 | 34 | GG+CC+GG+GG+AA+ GG+TT  GT+CG+AG+AA+AG+ AA+CT | | 0.12 | 6.92×${10}^{-1}$ | 2 | TT+GG+ AA+AG+  AG+AG +CT  GT+GG+ AG+AA+ AA+AA+ TT | | -1.08 | 9.65×${10}^{-3}$ | 0.137 |
|  | rs1065407+rs27044+rs26653+rs27434+rs469876+rs13167972+rs17482078 | 44 | GT+GG+CC+GG+AA+ AA+TT  TT+CG+CG+AG+AG+ AG+CT | | 0.31 | 2.80×${10}^{-1}$ | 2 | TT+CG+GG+AA+AG+ AG+CT  GT+GG +CG+AG+AA+AA +TT | | -1.08 | 9.65×${10}^{-3}$ | 0.150 |
|  | rs1065407+rs30187+rs27044+rs26653+rs27434+rs469876+rs17482078 | 24 | GG+TT+GG+CC+ GG+  GG+TT  GT+CT+CG+CG+AG+ AA+CT | | -0.05 | 8.94×${10}^{-1}$ | 2 | TT+CT+CG+GG+AA  +AG+CT  GT+TT+GG+CG+AG+AA+TT | | -1.08 | 9.65×${10}^{-3}$ | 0.157 |
| #8 Interaction Effects | rs1065407+rs2287987+rs10050860+rs26653+rs469876+rs17481856+rs13167972+rs17482078 | 30 | GT+CC +NA+CC+ GG +AA+GG+ TT  TT+TT+NA+GG+AA+ AG+AA+ CT | | 0.47 | 1.28×${10}^{-1}$ | 3 | TT+CT+NA++CG+AG+AG+ AG+CT  GT+TT+NA+GG+ AA+AA +AA+TT | | -1.00 | 1.60×${10}^{-3}$ | 0.017 |
|  | rs1065407+rs2287987+rs30187+rs10050860+rs27044+rs26653+rs469876+rs13167972 | 47 | GT+CC+CC+NA+CC+ CC+AA+AA  TT+TT+CT+NA+GG+ GG+AG+ AG | | 0.06 | 8.35×${10}^{-1}$ | 3 | TT+CT+TT+NA+CG+ CG+AG+ AG  GT+TT+CT+NA+GG+GG+AA+ AA | | -0.92 | 2.41×${10}^{-3}$ | 0.074 |
|  | rs1065407+rs2287987+rs30187+rs10050860+rs26653+rs27434+rs17481856+rs17482078 | 14 | GG+CC+CT+NA+CC+ GG+GG+ TT  GT+TT+CT+NA+CG+ AG+AA+ CT | | 0.24 | 5.10×${10}^{-1}$ | 2 | TT+CT+CT+NA+GG+ AA+AG+ CT  GT+TT+TT+NA+CG+AG+AA+ AG | | -0.96 | 4.53×${10}^{-3}$ | 0.010 |
|  | rs1065407+rs2287987+rs10050860+rs26653+rs27434+rs17481856+rs13167972+rs17482078 | 25 | GG+CC+NA+CC+GG+ AA+GG+ TT  TT+TT+NA+CG+AG+ AG+AA+CT | | 0.17 | 6.25×${10}^{-1}$ | 2 | TT+CT+NA+GG+AA+ AG+AG+ CT  GT+TT+NA+CG+AG+AA+AA+ TT | | -0.96 | 4.69×${10}^{-3}$ | 0.013 |
|  | rs1065407+rs2287987+rs30187+rs26653+rs27434+rs17481856+rs13167972+rs17482078 | 26 | GG+CT+TT+CC+GG+ GG+GG+TT  GT+TT+CT+CG+AG +AA+AA+ CT | | 0.23 | 5.03×${10}^{-1}$ | 2 | TT+TT+CT+GG+AA+AG+AG +CT  GT+CT+TT+CG+AG+AA+AA+ TT | | -0.95 | 4.85×${10}^{-3}$ | 0.019 |
|  | rs1065407+rs2287987+rs30187+rs10050860+rs26653+rs27434+rs13167972+rs17482078 | 27 | GG+CC+TT+NA+CC +GG+GG+ TT  GT+TT+CT+NA+CG +AG+AG+ AA+CT | | 0.17 | 6.21×${10}^{-1}$ | 2 | TT+CT+CT+NA+GG+AA+AG+ CT  GT+TT+TT+NA+CG+AG+AA+ TT | | -0.95 | 4.85×${10}^{-3}$ | 0.016 |
|  | rs1065407+rs2287987+rs30187+rs10050860+rs26653+rs27434+rs17481856+rs13167972 | 25 | GG+CC+TT+NA+CC+ GG+GG+ GG  GT+TT+CT+NA+CG +AG+AA +AA | | 0.18 | 6.11×${10}^{-1}$ | 2 | TT+CT+CT+NA+GG+AA+AG+ AG  GT+TT+CT+NA+CG+AG+AA+ AA | | -0.95 | 5.02×${10}^{-3}$ | 0.076 |
|  | rs1065407+rs2287987+rs10050860+rs27044+rs26653+rs27434+rs17481856+rs17482078 | 18 | GG+CC+NA+CC+CC+ GG+AA+ TT  GT+TT+NA+GG+CG+ AG+AG+ CT | | 0.21 | 5.30×${10}^{-1}$ | 2 | TT+CT+NA+CG+GG+ AA+AG+ CT  GT+TT+NA+GG+CG+ AG+AA+ TT | | -0.93 | 6.21×${10}^{-3}$ | 0.021 |
|  | rs1065407+rs2287987+rs30187+rs27044+rs26653+rs27434+rs17481856+rs17482078 | 18 | GG+TT+TT+GG+CC +GG+GG +TT  GT+CT+CG+GG+CG +AG+AA+ CC | | 0.33 | 3.29×${10}^{-1}$ | 2 | TT+CT+CT+CG+GG+AA+AG+ CT  GT+TT+TT+GG+CG+ AG+AA+ TT | | -0.93 | 6.42×${10}^{-3}$ | 0.014 |
|  | rs1065407+rs2287987+rs30187+rs10050860+rs27044+rs26653+rs27434+rs17482078 | 19 | GG+CC+TT+NA+GG+ CC+GG+TT  GT+TT+CT+NA+CG+ CG+AG+CT | | 0.27 | 4.23×${10}^{-1}$ | 2 | TT+CT+CT+NA+CG+ CG+AG+ CT  GT+TT+TT+ NA+GG +GG+AA +TT | | -0.93 | 6.42×${10}^{-3}$ | 0.011 |
|  | rs1065407+rs2287987+rs30187+rs10050860+rs27044+rs26653+rs27434+rs17481856 | 16 | GG+CC+TT+NA+GG+ CC+GG+ GG  GT+TT+CT+NA+CG+ CG+AG+ AA | | 0.29 | 4.09×${10}^{-1}$ | 2 | TT+CT+CT+NA+CG+ GG+AA+ AG  GT+TT+TT+NA+GG+ CG+AG+ AA | | -0.92 | 6.63×${10}^{-3}$ | 0.060 |
|  | rs1065407+rs2287987+rs27044+rs26653+rs27434+rs17481856+rs13167972+rs17482078 | 38 | GG+TT+CC+CC+GG +AA+AA +TT  GT+CT+GG+CG+AG+ AG+AG+CT | | 0.28 | 3.58×${10}^{-1}$ | 2 | TT+CT+CG+GG+AA+ AG+AG+ CT  GT+TT+GG+CG+AG+ AA+AA+ TT | | -0.92 | 6.63×${10}^{-3}$ | 0.021 |
|  | rs1065407+rs2287987+rs10050860+rs27044+rs26653+rs27434+rs13167972+rs17482078 | 36 | GG+TT+NA+GG+CC+ GG+GG+ TT  GT+CT+NA+CG+CG+ AG+AA+ CT | | 0.29 | 3.52×${10}^{-1}$ | 2 | TT+CT+NA+CG+GG+ AA+AG+ CT  GT+TT+NA+ GG+CG+AG+AA+ TT | | -0.92 | 6.63×${10}^{-3}$ | 0.024 |
|  | rs1065407+rs2287987+rs10050860+rs27044+rs26653+rs27434+rs17481856+rs13167972 | 37 | GG+CC+NA+CC+CC+ GG+AA+ AA  GT+TT+NA+GG+CG+ AG+AG+ AG | | 0.24 | 4.33×${10}^{-1}$ | 2 | TT+CT+NA+CG+GG+AA+AG+ AG  GT+TT+NA+GG+CG+AG+AA+ AA | | -0.92 | 6.86×${10}^{-3}$ | 0.067 |
|  | rs1065407+rs2287987+rs30187+rs27044+rs26653+rs27434+rs13167972+rs17482078 | 39 | GG+TT+TT+CC+CC+ GG+AA+TT  GT+CT+CT+GG+CG+ AG+AG+ CT | | 0.33 | 2.82×${10}^{-1}$ | 2 | TT+CT+CT+CG+GG+ AA+AG+ CT  GT+TT+TT+GG+CG+ AG+AA+ TT | | -0.92 | 6.86×${10}^{-3}$ | 0.015 |
|  | rs1065407+rs2287987+rs30187+rs27044+rs26653+rs27434+rs17481856+rs13167972 | 35 | GG+CC+TT+CC+CC+ GG+GG+ AA  GT+CT+CT+GG+CG+ AG+AA+ AG | | 0.40 | 2.07×${10}^{-1}$ | 2 | TT+CT+CT+CG+GG+AA+AG+ AG  GT+TT+TT+GG+CG+AG+AA+ AA | | -0.92 | 7.09×${10}^{-3}$ | 0.092 |
|  | rs1065407+rs2287987+rs30187+rs10050860+rs27044+rs26653+rs27434+rs13167972 | 38 | GG+CC+TT+NA+CC+ CC+GG+AA  GT+TT+CT+NA+GG+ CG+AG+ AG | | 0.29 | 3.45×${10}^{-1}$ | 2 | TT+CT+CT+NA+CG+GG+AA+ AG  GT+TT+TT+NA+GG+CG+AG+ AA | | -0.92 | 7.09×${10}^{-3}$ | 0.052 |
|  | rs1065407+rs2287987+rs30187+rs10050860+rs26653+rs17481856+rs13167972+rs17482078 | 26 | GG+CC+ CC+NA+CC+ AA+GG+ TT  GT+TT+CT+NA+ CG+AG +AA+CT | | 0.23 | 5.10×${10}^{-1}$ | 2 | TT+CT+TT+NA+GG+AG+AG+ CT  GT+TT+CT+NA+CG+ AA+AA+ TT | | -0.88 | 7.76×${10}^{-3}$ | 0.017 |
|  | rs1065407+rs2287987+rs27044+rs26653+rs27434+rs469876+rs17481856+rs17482078 | 27 | GG+TT+CC+CC+CC+ AA+AA+TT  GT+CT+GG+CG+AG+ AG+ CT | | 0.14 | 6.52×${10}^{-1}$ | 2 | TT+CT+ CG+GG +AA+AG+AG+ CT  GT+TT+GG+CG+AG+AA+AA+ TT | | -0.89 | 9.05×${10}^{-3}$ | 0.029 |
|  | rs1065407+rs2287987+rs10050860+rs26653+rs27434+rs469876+rs17481856+rs17482078 | 23 | GG+CC+NA+CC+GG+ GG+AA+TT  GT+TT+NA+CG+AG+ AA+AG+CT | | 0.04 | 8.86×${10}^{-1}$ | 2 | TT+CT+NA+GG+AA+AG+AG+ CT  GT+TT+NA+CG+AG+AA+AA+ TT | | -0.89 | 9.05×${10}^{-3}$ | 0.012 |
|  | rs1065407+rs2287987+rs10050860+rs27044+rs27434+rs469876+rs17481856+rs17482078 | 16 | GT+CC+NA+CC+AA+ AA+AA+TT  TT+TT+NA+GG+AG+AG+AG+CT | | -0.77 | 1.60×${10}^{-1}$ | 2 | TT+CT+NA+CG+AG+ AG+AG+ CT  GT+TT+NA+GG+AA+AA+AA+ TT | | -0.89 | 9.05×${10}^{-3}$ | 0.013 |
|  | rs1065407+rs2287987+rs10050860+rs27044+rs26653+rs27434+rs469876+rs17482078 | 26 | GG+CC+NA+GG+CC+ GG+AA+TT  GT+TT+NA+CG+CG+AG+AG+CT | | 0.18 | 5.46×${10}^{-1}$ | 2 | TT+CT+NA+CG+GG+AA+AG+ CT  GT+TT+NA+GG+CG+AG+AA+ TT | | -0.89 | 9.05×${10}^{-3}$ | 0.012 |
|  | rs1065407+rs10050860+rs27044+rs26653+rs27434+rs469876+rs17481856+rs17482078 | 25 | GG+CT+CC+CC+GG+ AA+TT  GT+TT+GG+ CG+AG+ AA+AG+CT | | 0.18 | 5.81×${10}^{-1}$ | 2 | TT+TT+CG+GG+AA+AG+AG+ CT  GT+CT+GG+CG+AG+ AA+AA+ TT | | -1.09 | 9.18×${10}^{-3}$ | 0.002 |
|  | rs1065407+rs2287987+rs10050860+rs27044+rs26653+rs27434+rs469876+rs17481856 | 26 | GG+CC+NA+CC+CC+ GG+GG+ AA  GT+TT+NA+GG+CG+AG+AA+ AG | | 0.10 | 7.54×${10}^{-1}$ | 2 | TT+CT+NA+CG+GG+ AA+AG+ AG  GT+TT+NA+GG+CG+AG+AA+ AA | | -0.89 | 9.35×${10}^{-3}$ | 0.073 |
|  | rs1065407+rs2287987+rs30187+rs26653+rs27434+rs469876+rs17481856+rs17482078 | 24 | GG+CT+TT+CC+GG+ GG+GG+TT  GT+TT+CT+CG+AG+ AA+AA+ CT | | 0.10 | 7.55×${10}^{-1}$ | 2 | TT+TT+CT+GG+AA+ AG+AG+ CT  GT+CT+TT+CG+AG+AA+AA +TT | | -0.89 | 9.35×${10}^{-3}$ | 0.025 |
|  | rs1065407+rs2287987+rs30187+rs27044+rs27434+rs469876+rs17481856+rs17482078 | 17 | GT+TT+TT+CC+GG+AA+GG+TT  TT+CT+CT+ GG+AG+ AG+AA+CT | | -0.58 | 2.62×${10}^{-1}$ | 2 | TT+CT+CT+CG+AA+AG+AG+ CT  GT+TT+TT+GG+AG+ AA+AA+ TT | | -0.89 | 9.35×${10}^{-3}$ | 0.016 |
|  | rs1065407+rs2287987+rs30187+rs10050860+rs27434+rs469876+rs17481856+rs17482078 | 13 | GG+CC+TT+NA+GG+AA+GG+TT  GT+TT+CT+NA+AG+AG+AA+CT | | -1.18 | 7.47×${10}^{-2}$ | 2 | TT+CT+CT+NA+AA+AG+AG +CT  GT+TT+TT+NA+AG+AA+AA+ TT | | -0.89 | 9.35×${10}^{-3}$ | 0.012 |
|  | rs1065407+rs2287987+rs30187+rs27044+rs26653+rs27434+rs469876+rs17482078 | 28 | GG+TT+TT+CC+CC+GG+GG+TT  GT+CT+CT+GG+CG+ AG+AA+CT | | 0.18 | 5.40×${10}^{-1}$ | 2 | TT+CT+CT+CG+GG+AA+AG+ CT  GT+TT+TT+GG+CG+AG+AA+ TT | | -0.89 | 9.35×${10}^{-3}$ | 0.025 |
|  | rs1065407+rs2287987+rs30187+rs10050860+rs26653+rs27434+rs469876+rs17482078 | 25 | GG+CC+TT+NA+CC+ GG+GG+TT  GT+TT+ CT+NA+CG+AG+ AA+CT | | 0.05 | 8.73×${10}^{-1}$ | 2 | TT+CT+CT+NA+GG+AA+AG+ CT  GT+TT+TT+NA+CG+AG+AA+TT | | -0.89 | 9.35×${10}^{-3}$ | 0.006 |
|  | rs1065407+rs2287987+rs30187+rs10050860+rs27044+rs27434+rs469876+rs17482078 | 18 | GT+CC+CT+NA+CC+ AA+AA+ TT  TT+TT+TT+ NA+GG+ AG+AG+ CT | | -0.67 | 1.88×${10}^{-1}$ | 2 | TT+CT+TT+NA+ CG+ AG+AG+ CT  GT+TT+CT+NA+GG+AA+AA+ TT | | -0.89 | 9.35×${10}^{-3}$ | 0.013 |
|  | rs1065407+rs2287987+rs10050860+rs27044+rs27434+rs469876+rs17481856+rs28096 | 33 | GT+CC+NA+CC+GG+ AA+AA+ GG  TT+TT+NA+GG+AA+ AG+AG+ AG | | -0.89 | 9.35×${10}^{-3}$ | 0 | NA | | NA | NA | 0.091 |
|  | rs1065407+rs27044+rs26653+rs27434+rs469876+rs17481856+rs13167972+rs17482078 | 46 | GG+CC+CC+GG+AA+ AA+AA+ TT  GT+TT+CG+AG+AG+ AG+AG+ CT | | 0.22 | 4.29×${10}^{-1}$ | 2 | TT+CG+GG+ AA+AG+ AG+AG+ CT  GT+GG +CG+AG+AA+AA+ AA+ TT | | -1.08 | 9.65×${10}^{-3}$ | 0.147 |
|  | rs1065407+rs10050860+rs26653+rs27434+rs469876+rs17481856+rs13167972+rs17482078 | 35 | GG+CT+CC+GG+GG+ AA+GG+TT  GT+TT+CG+AG+AA+ AG+AA+ CT | | 0.07 | 8.12×${10}^{-1}$ | 2 | TT+TT+GG+AA+AG+ AG+AG+ CT  GT+CT+CG+AG+AA+ AA+AA+ TT | | -1.08 | 9.65×${10}^{-3}$ | 0.002 |
|  | rs1065407+rs10050860+rs27044+rs26653+rs27434+rs469876+rs13167972+rs17482078 | 45 | GT+CT+GG+CC+GG+AA+GG+TT  TT+TT+CG+CG+AG+ AG+AA+CT | | 0.26 | 3.50×${10}^{-1}$ | 2 | TT+TT+CG+GG+AA+AG+AG+ CT  GT+CT+GG+CG+AG+AA+AA+ TT | | -1.08 | 9.65×${10}^{-3}$ | 0.002 |
|  | rs1065407+rs30187+rs27044+rs26653+rs27434+rs469876+rs17481856+rs17482078 | 24 | GG+TT+GG+CC+GG +GG+GG+ TT  GT+CT+ CG+CG+AG+AA+AA+ CT | | -0.05 | 8.94×${10}^{-1}$ | 2 | TT+CT+CG+GG+AA+AG+AG +CT  GT+TT+GG+CG+AG+ AA+AA+ TT | | -1.08 | 9.65×${10}^{-3}$ | 0.105 |
|  | rs1065407+rs30187+rs10050860+rs27044+rs26653+rs27434+rs469876+rs17482078 | 25 | GG+TT+CT+CC+CC+ GG+GG+TT  GT+CT+TT+GG+CG+ AG+AA+ CT | | -0.13 | 7.47×${10}^{-1}$ | 2 | TT+CT+TT+CG+GG+AA+AG+ CT  GT+TT+CT+GG+CG+AG+AA+ TT | | -1.08 | 9.65×${10}^{-3}$ | 0.002 |
|  | rs1065407+rs2287987+rs30187+rs27044+rs26653+rs27434+rs469876+rs17481856 | 25 | GG+CC+TT+CC+CC+ GG+GG+  GG  GT+TT+CT+GG+CG +AG+AA +AA | | 0.24 | 4.33×${10}^{-1}$ | 2 | TT+CT+CT+CG+GG+AA+AG +AG  GT+TT+TT+GG+CG+AG+AA+ AA | | -0.88 | 9.65×${10}^{-3}$ | 0.088 |
|  | rs1065407+rs2287987+rs30187+rs10050860+rs26653+rs27434+rs469876+rs17481856 | 23 | GG+CC+TT+NA+CC +GG+GG+ GG  GT+TT+CT+NA+CG +AG+AA+ AA | | 0.05 | 8.67×${10}^{-1}$ | 2 | TT+CT+CT+NA+GG+ AA+AG+ AG  GT+TT+TT+NA+CG+AG+AA+ AA | | -0.88 | 9.65×${10}^{-3}$ | 0.085 |
|  | rs1065407+rs2287987+rs30187+rs10050860+rs27044+rs27434+rs469876+rs17481856 | 16 | GT+CC+TT+NA+CC+ GG+AA+ GG  TT+TT+CT+NA+GG +AG+AG+ AA | | -0.76 | 1.64×${10}^{-1}$ | 2 | TT+CT+CT+NA+CG+AA+AG+ AG  GT+TT+TT+NA+GG+AG+AA+ AA | | -0.88 | 9.65×${10}^{-3}$ | 0.081 |
|  | rs1065407+rs2287987+rs30187+rs10050860+rs27044+rs26653+rs27434+rs469876 | 27 | GG+CC+CT+NA+GG +CC+GG +GG  GT+TT+TT+NA+CG+ CG+AG + AA | | 0.14 | 6.34×${10}^{-1}$ | 2 | TT+CT+TT+NA+CG+GG+AA+ AG  GT+TT+CT+NA+GG+CG+AG+ AA | | -0.88 | 9.65×${10}^{-3}$ | 0.056 |
|  | rs1065407+rs2287987+rs26653+rs27434+rs469876+rs17481856+rs13167972+rs17482078 | 37 | GG+CT+CC+GG+GG +AA+GG +TT  GT+TT+CG+AG+AA+ AG+AA+ CT | | -0.09 | 7.77×${10}^{-1}$ | 2 | TT+TT+GG+AA+AG+AG+AG+ CT  GT+CT+CG+AG+AA+AA+AA+ TT | | -0.88 | 9.66×${10}^{-3}$ | 0.029 |
|  | rs1065407+rs2287987+rs27044+rs27434+rs469876+rs17481856+rs13167972+rs17482078 | 37 | GG+TT+CC+AA+AA+ AA+AA+TT  GT+CT+GG+AG+AG+ AG+AG+CT | | 0.49 | 1.44×${10}^{-1}$ | 2 | TT+CT+CG+AG+AG+AG+AG+ CT  GT+TT+GG+AA+AA+ AA+AA+ TT | | -0.88 | 9.66×${10}^{-3}$ | 0.024 |
|  | rs1065407+rs2287987+rs10050860+rs27434+rs469876+rs17481856+rs13167972+rs17482078 | 27 | GT+CC+ NA+GG+ AA+AA+ GG+ TT  TT+TT+NA+AA+AG+ AG+AA+CT | | 0.19 | 6.09×${10}^{-1}$ | 2 | TT+CT+NA+AG+AG+ AG+AG+ CT  GT+TT+NA+ AA+AA+ AA+AA+ TT | | -0.88 | 9.66×${10}^{-3}$ | 0.014 |
|  | rs1065407+rs2287987+rs27044+rs26653+rs27434+rs469876+rs13167972+rs17482078 | 47 | GG+TT+CC+CC+GG+ AA+AA+TT  GT+CT+GG+CG+AA+ AG+AG+CT | | 0.13 | 6.37×${10}^{-1}$ | 2 | TT+CT+ CG+GG+ AA+AG+ AG+CT  GT+TT+GG+CG+AG+AA+AA+ TT | | -0.88 | 9.66×${10}^{-3}$ | 0.029 |
|  | rs1065407+rs2287987+rs10050860+rs27044+rs27434+rs469876+rs13167972+rs17482078 | 36 | GT+CC+NA+CC+AA+ AA+AA+TT  TT+TT+NA+AA+AG+ AG+AG+CT | | 0.56 | 1.02×${10}^{-1}$ | 2 | TT+CT+NA+CG+AG+AG+AG+ CT  GT+TT+NA+GG+AA+AA+AA +TT | | -0.88 | 9.66×${10}^{-3}$ | 0.023 |
|  | rs1065407+rs30187+rs10050860+rs27434+rs17481856+rs28096+rs13167972+rs17482078 | 29 | GT+TT+CT+GG+GG +AA+GG+ TT  TT+CT+TT+AG+AA+ AG+AA+CT | | 1.09 | 9.66×${10}^{-3}$ | 0 | NA | | NA | NA | 0.002 |
|  | rs1065407+rs10050860+rs27044+rs26653+rs27434+rs469876+rs17481856+rs13167972 | 44 | GG+TT+CC+CC+GG+ GG+AA+ AA  GT+CT+GG+CG+AG +AA+ AG+AG | | 0.23 | 4.16×${10}^{-1}$ | 2 | TT+CT+CG+GG+AA+AA+AG +AG  GT+TT+GG+CG+AG+AG+AA+ AA | | -1.08 | 9.89×${10}^{-3}$ | 0.193 |
|  | rs1065407+rs30187+rs10050860+rs27044+rs26653+rs27434+rs469876+rs17481856 | 23 | GG+TT+TT+GG+CC+ GG+GG +GG  GT+CT+CT+CG+CG+ AG+AA +AA | | 0.33 | 3.03×${10}^{-1}$ | 2 | TT+CT+CT+CG+GG+AA+AG+ AG  GT+TT+TT+GG+CG+AG+AA+ AA | | -1.08 | 9.89×${10}^{-3}$ | 0.082 |
|  | rs1065407+rs2287987+rs27044+rs26653+rs27434+rs469876+rs17481856+rs13167972 | 46 | GG+CC+GG+CC+GG+ AA+AA+ AA  GT+TT+GG+CG+AG+ AG+AG+ AG | | 0.10 | 7.29×${10}^{-1}$ | 2 | TT+CT+CG+GG+AA+AG+AG+ AG  GT+TT+GG+CG+AG+AA+AA+ AA | | -0.88 | 9.97×${10}^{-3}$ | 0.191 |
|  | rs1065407+rs2287987+rs10050860+rs26653+rs27434+rs469876+rs17481856+rs13167972 | 36 | GG+CC+NA+CC+GG+ GG+AA+ GG  GT+TT+NA+CG+AG+ AA+AG+ AA | | -0.13 | 6.67×${10}^{-1}$ | 2 | TT+CT+NA+GG+AA+ AG+AG+ AG  GT+TT+NA+CG+AG+AA+AA+ AA | | -0.88 | 9.97×${10}^{-3}$ | 0.082 |
|  | rs1065407+rs2287987+rs10050860+rs27044+rs27434+rs469876+rs17481856+rs13167972 | 36 | GT+CC+NA+CC+AA +AA+AA +AA  TT+TT+NA+GG+AG+ AG+AG+ AG | | 0.44 | 1.87×${10}^{-1}$ | 2 | TT+CT+NA+CG+AG+AG+AG+ AG  GT+TT+NA+GG+AA+AA+AA+ AA | | -0.88 | 9.97×${10}^{-3}$ | 0.066 |
|  | rs1065407+rs2287987+rs10050860+rs27044+rs26653+rs27434+rs469876+rs13167972 | 46 | GG+CC+NA+CC+CC+GG+AA+AA  GT+TT+NA+GG+CG+AG+AG+AG | | 0.10 | 7.29×${10}^{-1}$ | 2 | TT+CT+NA+CG+GG+AA+AG +AG  GT+TT+NA+GG+CG+AG+AA +AA | | -0.88 | 9.97×${10}^{-3}$ | 0.076 |
|  | rs1065407+rs2287987+rs30187+rs27434+rs469876+rs17481856+rs13167972+rs17482078 | 29 | GG+CT+TT+GG+AA+GG+AA+TT  GT+TT+CT+AG+AGAA+AG+CT | | 0.19 | 6.06×${10}^{-1}$ | 2 | TT+TT+CT+AA+AG+AG+AG +CT  GT+CT+TT+AG+AA+AA+AA +TT | | -0.88 | 9.97×${10}^{-3}$ | 0.058 |
|  | rs1065407+rs2287987+rs30187+rs26653+rs27434+rs469876+rs13167972+rs17482078 | 39 | GG+TT+CT+CC+GG+GG+GG+TT  GT+CT+CT+CG+AG+AA+AA+CT | | -0.08 | 7.97×${10}^{-1}$ | 2 | GT+TT+CT+GG+AA+AG+AG +CT  TT+CT+TT+CG+AG+AA+AA+ TT | | -0.88 | 9.97×${10}^{-3}$ | 0.033 |
|  | rs1065407+rs2287987+rs30187+rs27044+rs27434+rs469876+rs13167972+rs17482078 | 39 | GG+TT+TT+CC+AA+ AA+AA+CT  GT+CT+CT+GG+AG +AG+AG+ TT | | 0.47 | 1.48×${10}^{-1}$ | 2 | TT+CT+CT+CG+AG+AG+AG +CT  GT+TT+TT+GG+AA+AA+AA+ TT | | -0.88 | 9.97×${10}^{-3}$ | 0.021 |
|  | rs1065407+rs2287987+rs30187+rs10050860+rs27434+rs469876+rs13167972+rs17482078 | 30 | GT+CC+CC+NA+AA+ AA+GG+TT  TT+TT+CT+NA+AG+ AA+CT | | 0.12 | 7.39×${10}^{-1}$ | 2 | TT+CT+CT+NA+AG+AG+AG+ CT  GT+TT+TT+NA+AA+AA+AA+ TT | | -0.88 | 9.97×${10}^{-3}$ | 0.026 |
|  | rs1065407+rs2287987+rs30187+rs10050860+rs27044+rs26653+rs17481856+rs17482078 | 18 | GG+CC+CC+NA+CC+ CC+AA+TT  GT+TT+CT+NA+GG+ CG+AG+CT | | 0.33 | 3.35×${10}^{-1}$ | 2 | TT+CT+TT+NA+CG+GG+AG+ CT  GT+TT+CT+NA+GG+ CG+AA+TT | | -0.85 | 1.02×${10}^{-2}$ | 0.016 |
|  | rs1065407+rs2287987+rs30187+rs26653+rs27434+rs469876+rs17481856+rs13167972 | 35 | GG+CC+TT+CC+GG +GG+GG +AA  GT+TT+CT+CG+AG+ AA+AA +AG | | -0.03 | 9.12×${10}^{-1}$ | 2 | TT+CT+CTGG+AA+ AG+AG+ AG  GT+TT+TT+CG+AG+AA+AA +AA | | -0.88 | 1.03×${10}^{-2}$ | 0.119 |
| #9 Interaction Effects | rs1065407+rs2287987+rs30187+rs10050860+rs26653+rs27434+rs17481856+rs13167972+rs17482078 | 27 | GG+CC+TT+NA+CC+GG+GG+GG+TT  GT+TT+CT+NA+CG+AG+AA+AA+CT | | 0.17 | 6.21×${10}^{-1}$ | 2 | TT+CT+CT+NA+GG+AA+AG+AG+CT  GT+TT+TT+NA+CG+AG+AA+AA+TT | | -0.95 | 0.005 | 0.024 |
|  | rs1065407+rs2287987+rs30187+rs10050860+rs27044+rs26653+rs27434+rs17481856+rs17482078 | 19 | GG+CC+TT+NA+GG+CC+GG+GG+TT  GT+TT+CT+NA+CG+CG+AG+AA+CT | | 0.27 | 4.23×${10}^{-1}$ | 2 | TT+CT+CT+NACG+GG+AA+AG+CT  GT+TT+TT+NA+GG+CG+AG+AA+TT | | -0.93 | 0.006 | 0.016 |
|  | rs1065407+rs2287987+rs10050860+rs27044+rs26653+rs27434+rs17481856+rs13167972+rs17482078 | 39 | GG+TT+NA+CC+CC+GG+AA+AA+TT  GT+CT+NA+GG+CG+AG+AG+AG+CT | | 0.23 | 4.45×${10}^{-1}$ | 2 | TT+CT+NA+CG+GG+AA+AG+AG+CT  GT+TT+NA+GG+CG+AG+AA+AA+TT | | -0.92 | 0.007 | 0.014 |
|  | rs1065407+rs2287987+rs30187+rs27044+rs26653+rs27434+rs17481856+rs13167972+rs17482078 | 39 | GG+TT+TT+CC+CC+GG+GG+AA+TT  GT+CT+CT+GG+CG+AG+AA+AG+CT | | 0.33 | 2.82×${10}^{-1}$ | 2 | TT+CT+CT+CG+GG+AA+AG+AG+CT  GT+TT+TT+GG+CG+AG+AA+AA+TT | | -0.92 | 0.007 | 0.020 |
|  | rs1065407+rs2287987+rs30187+rs10050860+rs27044+rs26653+rs27434+rs13167972+rs17482078 | 40 | GG+CC+TT+NA+CC+CC+GG+AA+TT  GT+TT+CT+NA+GG+CG+AG+AG+CT | | 0.28 | 3.57×${10}^{-1}$ | 2 | TT+CT+CT+NA+CG+GG+AA+AG+CT  GT+TT+TT+NA+GG+CG+AG+AA+TT | | -0.92 | 0.007 | 0.006 |
|  | rs1065407+rs2287987+rs30187+rs10050860+rs27044+rs26653+rs27434+rs17481856+rs13167972 | 38 | GG+CC+TT+NA+CC+CC+GG+GG+AA  GT+TT+CT+NA+GG+CG+AG+AA+CT | | 0.29 | 3.45×${10}^{-1}$ | 2 | TT+CT+CT+NA+CG+GGAA+AG+AG  GT+TT+TT+ NA+GG+CG+AG+AA+AA | | -0.92 | 0.007 | 0.053 |
|  | rs1065407+rs2287987+rs10050860+rs27044+rs26653+rs27434+rs469876+rs17481856+rs17482078 | 28 | GG+CC+NA+CC+CC+GG+AA+AA+TT  GT+TT+NA+GG+CG+AG+AG+AG+CT | | 0.09 | 7.63×${10}^{-1}$ | 2 | TT+CT+NA+CG+GG+AA+AG+AG+CT  GT+TT+NA+GG+CG+AG+AA+AA+TT | | -0.89 | 0.009 | 0.013 |
|  | rs1065407+rs2287987+rs30187+rs27044+rs26653+rs27434+rs469876+rs17481856+rs17482078 | 28 | GG+TT+TT+CC+CC+GG+GG+GG+TT  GT+CT+CT+GG+CG+AG+AA+AA+CT | | 0.18 | 5.40×${10}^{-1}$ | 2 | TT+CT+CT+CG+GG+AA+AG+AG+CT  GT+TT+TT+GG+CG+AG+AA+AA+TT | | -0.89 | 0.009 | 0.029 |
|  | rs1065407+rs2287987+rs30187+rs10050860+rs26653+rs27434+rs469876+rs17481856+rs17482078 | 25 | GG+CC+TT+NA+CC+GG+GG+GG+TT  GT+TT+CT+NA+CG+AG+AA+AA+CT | | 0.05 | 8.73×${10}^{-1}$ | 2 | TT+CT+CT+NA+GG+AA+AG+AG+CT  GT+TT+TT+NA+CG+AG+AA+AA+TT | | -0.89 | 0.009 | 0.015 |
|  | rs1065407+rs2287987+rs30187+rs10050860+rs27044+rs27434+rs469876+rs17481856+rs17482078 | 18 | GT+CC+TT+NA+CC+GG+AA+GG+TT  TT+TT+CT+NA+GG+AG+AG+AA+CT | | -0.67 | 1.×${10}^{-1}$ | 2 | TT+CT+CT+NA+CG+AA+  AG+AG+CT  GT+TT+TT+NA+GG+AG+AA+AA+TT | | -0.89 | 0.009 | 0.023 |
|  | rs1065407+rs2287987+rs30187+rs10050860+rs27044+rs26653+rs27434+rs469876+rs17482078 | 29 | GG+CC+CT+NA+CC+CC+GG+GG+TT  GT+TT+TT+NA+GG+CG+AG+AA+CT | | 0.14 | 6.44×${10}^{-1}$ | 2 | TT+CT+TT+NA+CG+GG+AA+AG+CT  GT+TT+CT+NA+GG+CG+AG+AA+TT | | -0.89 | 0.009 | 0.013 |
|  | rs1065407+rs10050860+rs27044+rs26653+rs27434+rs469876+rs17481856+rs13167972+rs17482078 | 47 | GG+CT+CC+CC+GG+GG+AA+AA+TT  GT+TT+GG+CG+AG+AA+AG+AG+CT | | 0.18 | 5.17×${10}^{-1}$ | 2 | TT+TT+CG+GG+AA+AG+AG+AG+CT  GT+CT+GG+CG+AG+AA+AA+AA+TT | | -1.08 | 0.010 | 0.002 |
|  | rs1065407+rs30187+rs10050860+rs27044+rs26653+rs27434+rs469876+rs17481856+rs17482078 | 25 | GG+TT+CT+CC+CC+GG+GG+GG+TT  GT+CT+TT+GG+CG+AG+AA+AA+CT | | -0.13 | 7.47×${10}^{-1}$ | 2 | TT+CT+TT+CG+GG+AA+AG+AG+CT  GT+TT+CT+GG+CG+AG+AA+AA+TT | | -1.08 | 0.010 | 0.002 |
|  | rs1065407+rs2287987+rs30187+rs10050860+rs27044+rs26653+rs27434+rs469876+rs17481856 | 27 | GG+CC+TT+NA+GG+CC+GG+GG+GG  GT+TT+CT+NA+CG+CG+AG+AA+AA | | 0.14 | 6.34×${10}^{-1}$ | 2 | TT+CT+CT+NA+CG+GG+AA+AG+AG  GT+TT+TT+NA+GG+CG+AG+AA+AA | | -0.88 | 0.010 | 0.052 |
|  | rs1065407+rs2287987+rs27044+rs26653+rs27434+rs469876+rs17481856+rs13167972+rs17482078 | 49 | GG+TT+CC+CC+GG+AA+AA+AA+TT  GT+CT+GG+CG+AG+AG+AG+AG+CT | | 0.05 | 8.42×${10}^{-1}$ | 2 | TT+CT+CG+GG+AA+AG+AG+AG+CT  GT+TT+GG+CG+AG+AA+AA+AA+TT | | -0.88 | 0.010 | 0.022 |
|  | rs1065407+rs2287987+rs10050860+rs26653+rs27434+rs469876+rs17481856+rs13167972+rs17482078 | 38 | GG+CC+NA+CC+GG+GG+AA+GG+TT  GT+TT+NA+CG+AG+AA+AG+AA+CT | | -0.13 | 6.69×${10}^{-1}$ | 2 | TT+CT+NA+GG+AA+AG+AG+AG+CT  GT+TT+NA+CG+AG+AA+AA+AA+TT | | -0.88 | 0.010 | 0.017 |
|  | rs1065407+rs2287987+rs10050860+rs27044+rs27434+rs469876+rs17481856+rs13167972+rs17482078 | 38 | GT+CC+NA+CC+AA+AA+AA+AA+TT  TT+TT+NA+GG+AG+AG+AG+AG+CT | | 0.42 | 1.98×${10}^{-1}$ | 2 | TT+CT+NA+CG+AG+AG+AG+AG+CT  GT+TT+NA+GG+AA+AA+AA+AA+TT | | -0.88 | 0.010 | 0.013 |
|  | rs1065407+rs2287987+rs10050860+rs27044+rs26653+rs27434+rs469876+rs13167972+rs17482078 | 48 | GG+TT+NA+CC+CC+GG+AA+AA+TT  GT+CT+NA+GG+CG+AG+AG+AG+CT | | 0.09 | 7.38×${10}^{-1}$ | 2 | TT+CT+NA+CG+GG+AA+AG+AG+CT  GT+TT+NA+GG+CG+AG+AA+AA+TT | | -0.88 | 0.010 | 0.013 |
|  | rs1065407+rs2287987+rs10050860+rs27044+rs26653+rs27434+rs469876+rs17481856+rs13167972 | 48 | GG+CC+NA+CC+CC+GG+AA+AA+AA  GT+TT+NA+GG+CG+AG+AG+AG+AG | | 0.02 | 9.42×${10}^{-1}$ | 2 | TT+CT+NA+CG+GG+AA+AG+AG+AG  GT+TT+NA+GG+CG+AG+AA+AA+AA | | -0.88 | 0.010 | 0.077 |
|  | rs1065407+rs2287987+rs30187+rs26653+rs27434+rs469876+rs17481856+rs13167972+rs17482078 | 39 | GG+CT+TT+CC+GG+GG+GG+AA+TT  GT+TT+CT+CG+AG+AA+AA+AG+CT | | -0.08 | 7.97×${10}^{-1}$ | 2 | TT+TT+CT+GG+AA+AG+AG+AG+CT  GT+CT+TT+CG+AG+AA+AA+AA+TT | | -0.88 | 0.010 | 0.015 |
|  | rs1065407+rs2287987+rs30187+rs27044+rs27434+rs469876+rs17481856+rs13167972+rs17482078 | 39 | GT+TT+TT+CC+GG+AA+GG+AA+TT  TT+CT+CT+GG+AG+AG+AA+AG+CT | | 0.47 | 1.48×${10}^{-1}$ | 2 | TT+CT+CT+CG+AA+AG+AG+AG+CT  GT+TT+TT+GG+AG+AA+AA+AA+TT | | -0.88 | 0.010 | 0.020 |
|  | rs1065407+rs2287987+rs30187+rs10050860+rs27434+rs469876+rs17481856+rs13167972+rs17482078 | 30 | GT+CC+TT+NA+GG+AA+GG+GG+TT  TT+TT+CT+NA+AG+AG+ AA+AA+CT | | 0.12 | 7.39×${10}^{-1}$ | 2 | TT+CT+CT+NA+AA+AG+AG+AG+CT  GT+TT+TT+NA+AG+AA+AA+AA+TT | | -0.88 | 0.010 | 0.023 |
|  | rs1065407+rs2287987+rs30187+rs27044+rs26653+rs27434+rs469876+rs13167972+rs17482078 | 50 | GG+TT+TT+CC+CC+GG+AA+AA+TT  GT+CT+CT+GG+CG+AG+AG+AG+CT | | 0.10 | 7.26×${10}^{-1}$ | 2 | TT+CT+CT+CG+GG+AA+AG+AG+CT  GT+TT+TT+GG+CG+AG+AA+AA+TT | | -0.88 | 0.010 | 0.026 |
|  | rs1065407+rs2287987+rs30187+rs10050860+rs26653+rs27434+rs469876+rs13167972+rs17482078 | 40 | GG+CC+TT+NA+CC+GG+GG+GG+TT  GT+TT+CT+NA+CG+AG+AA+AA+CT | | -0.12 | 6.90×${10}^{-1}$ | 2 | TT+CT+CT+NA+GG+AA+AG+AG+CT  GT+TT+TT+NA+CG+AG+AA+AA+TT | | -0.88 | 0.010 | 0.011 |
|  | rs1065407+rs2287987+rs30187+rs10050860+rs27044+rs27434+rs469876+rs13167972+rs17482078 | 40 | GT+CC+CT+NA+CC+AA+AA+AA+TT  TT+TT+TT+NA+GG+AG+AG+AG+CT | | 0.41 | 2.00×${10}^{-1}$ | 2 | TT+CT+TT+NA+CG+AG+AG+AG+CT  GT+TT+CT+NA+GG+AA+AA+AA+TT | | -0.88 | 0.010 | 0.009 |
|  | rs1065407+rs2287987+rs30187+rs27044+rs26653+rs27434+rs469876+rs17481856+rs13167972 | 47 | GG+CC+TT+CC+CC+GG+AA+GG+AA  GT+TT+CT+GG+CG+AG+AG+AA+AG | | 0.14 | 6.17×${10}^{-1}$ | 2 | TT+CT+CT+CG+GG+AA+AG+AG+AG  GT+TT+TT+GG+CG+AG+AA+AA+AA | | -0.88 | 0.010 | 0.093 |
|  | rs1065407+rs2287987+rs30187+rs10050860+rs26653+rs27434+rs469876+rs17481856+rs13167972 | 38 | GG+CC+TT+NA+CC+GG+GG+GG+AA  GT+TT+CT+NA+CG+AG+AA+AA+AG | | -0.12 | 6.88×${10}^{-1}$ | 2 | TT+CT+CT+NA+GG+AA+AG+AG+AG  GT+TT+TT+NA+CG+AG+AA+AA+AA | | -0.88 | 0.010 | 0.073 |
|  | rs1065407+rs2287987+rs30187+rs10050860+rs27044+rs27434+rs469876+rs17481856+rs13167972 | 38 | GT+CC+TT+NA+CC+GG+AA+GG+AA  TT+TT+CT+NA+GG+AG+AG+AA+AG | | 0.43 | 1.90×${10}^{-1}$ | 2 | TT+CT+CT+NA+CG+AA+AG+AG+AG  GT+TT+TT+NA+GG+AG+AA+AA+AA | | -0.88 | 0.010 | 0.062 |
|  | rs1065407+rs2287987+rs30187+rs10050860+rs27044+rs26653+rs27434+rs469876+rs13167972 | 49 | GG+CC+TT+NA+CC+CC+GG+GG+AA  GT+TT+CT+NA+GG+CG+AG+AA+AG | | 0.06 | 8.20×${10}^{-1}$ | 2 | TT+CT+CT+NA+CG+GG+AA+AG+AG  GT+TT+TT+NA+GG+CG+AG+AA+AA | | -0.88 | 0.010 | 0.065 |
|  | rs1065407+rs2287987+rs10050860+rs27044+rs27434+rs469876+rs17481856+rs28096+rs17482078 | 35 | GG+CC+NA+CC+GG+AA+AA+GG+TT  GT+TT+NA+GG+AA+AG+AG+AG+CT | | -0.85 | 1.05×${10}^{-2}$ | 0 | NA | | NA | NA | 0.021 |
|  | rs1065407+rs2287987+rs30187+rs10050860+rs27044+rs26653+rs17481856+rs13167972+rs17482078 | 39 | GG+TT+CC+NA+CC+CC+AA+AA+TT  GT+CT+CT+NA+GG+CG+AG+AG+CT | | 0.32 | 2.88×${10}^{-1}$ | 2 | TT+CT+TT+NA+CG+GG+AG+AG+CT  GT+TT+CT+NA+GG+CG+AA+AA+TT | | -0.85 | 0.011 | 0.011 |
|  | rs1065407+rs2287987+rs30187+rs10050860+rs26653+rs27434+rs17481856+rs28096+rs17482078 | 29 | GG+CC+TT+NA+CC+GG+GG+GG+TT  GT+TT+CT+NA+CG+AG+AA+AG+CT | | -0.16 | 6.64×${10}^{-1}$ | 2 | TT+CT+CT+NA+GG+AA+AG+AA+CT  GT+TT+TT+NA+CG+AG+AA+AG+TT | | -0.85 | 0.013 | 0.008 |
|  | rs1065407+rs2287987+rs10050860+rs26653+rs27434+rs17481856+rs28096+rs13167972+rs17482078 | 48 | GG+CC+NA+CC+GG+AA+GG+GG+TT  GT+TT+NA+CG+AG+AG+AG+AA+CT | | 0.05 | 8.59×${10}^{-1}$ | 2 | TT+CT+NA+CG+AG+AG+AG+AA+CT  GT+TT+NA+GG+AA+AA+AA+AG+TT | | -0.85 | 0.013 | 0.014 |
|  | rs1065407+rs2287987+rs30187+rs26653+rs27434+rs17481856+rs28096+rs13167972+rs17482078 | 49 | GG+TT+TT+CC+GG+GG+GG+GG+TT  GT+CT+CT+CG+AG+AA+AG+AA+CT | | 0.09 | 7.39×${10}^{-1}$ | 2 | TT+CT+CT+GG+AA+AG+AA+AG+CT  GT+TT+TT+CG+AG+AA+AG+AA+TT | | -0.85 | 0.014 | 0.016 |
|  | rs1065407+rs2287987+rs30187+rs10050860+rs26653+rs27434+rs28096+rs13167972+rs17482078 | 50 | GG+CC+TT+NA+CC+GG+GG+GG+TT  GT+TT+CT+NA+CG+AG+AG+AA+CT | | 0.05 | 8.45×${10}^{-1}$ | 2 | TT+CT+CT+NA+GG+AA+AA+AG+CT  GT+TT+TT+NA+CG+AG+AG+AA+TT | | -0.85 | 0.014 | 0.021 |
|  | rs1065407+rs2287987+rs30187+rs10050860+rs26653+rs27434+rs17481856+rs28096+rs13167972 | 48 | GG+CC+TT+NA+CC+GG+GG+GG+GG  GT+TT+CT+NA+CG+AG+AA+AG+AA | | 0.06 | 8.38×${10}^{-1}$ | 2 | TT+CT+CT+NA+GG+AA+AG+AA+AG  GT+TT+TT+NA+CG+AG+AA+AG+AA | | -0.84 | 0.014 | 0059 |
|  | rs1065407+rs2287987+rs30187+rs10050860+rs27044+rs26653+rs469876+rs17481856+rs17482078 | 28 | GG+CC+CC+NA+CC+CC+AA+AA+TT  GT+TT+CT+NA+GG+CG+AG+AG+CT | | 0.18 | 5.49×${10}^{-1}$ | 2 | TT+CT+TT+NA+CG+GG+AG+AG+CT  GT+TT+CT+NA+GG+CG+AA+AA+TT | | -0.81 | 0.015 | 0.019 |
|  | rs1065407+rs2287987+rs10050860+rs27044+rs26653+rs469876+rs17481856+rs13167972+rs17482078 | 37 | GT+CC+NA+CC+CC+AA+AA+AA+TT  TT+TT+NA+GG+CG+AG+AG+AG+CT | | 0.50 | 8.50×${10}^{-2}$ | 2 | TT+CT+NA+CG+GG+AG+AG+AG+CT  GT+TT+NA+GG+CG+AA+AA+AA+TT | | -0.81 | 0.015 | 0.011 |
|  | rs1065407+rs2287987+rs30187+rs10050860+rs27044+rs27434+rs469876+rs17481856+rs28096 | 34 | GT+CC+TT+NA+CC+GG+AA+GG+GG  TT+TT+CT+NA+GG+AG+AG+AA+AG | | -0.81 | 1.56×${10}^{-2}$ | 0 | NA | | NA | NA | 0.058 |
|  | rs1065407+rs2287987+rs30187+rs27044+rs26653+rs469876+rs17481856+rs13167972+rs17482078 | 49 | GG+TT+CC+CC+CC+AA+AA+AA+TT  GT+CT+CT+GG+CG+AG+AG+AG+CT | | 0.13 | 6.37×${10}^{-1}$ | 2 | TT+CT+TT+CG+GG+AG+AG+AG+CT  GT+TT+CT+GG+CG+AA+AA+AA+TT | | -0.81 | 0.016 | 0.020 |
|  | rs1065407+rs2287987+rs30187+rs10050860+rs26653+rs469876+rs17481856+rs13167972+rs17482078 | 39 | GG+CC+CC+NA+CC+GG+AA+GG+TT  GT+TT+CT+NA+CG+AA+AG+AA+CT | | -0.08 | 7.87×${10}^{-1}$ | 2 | TT+CT+TT+NA+CG+AG+AG+AG+CT  GT+TT+CT+NA+GG+AA+AA+AA+TT | | -0.81 | 0.016 | 0.020 |
|  | rs1065407+rs2287987+rs30187+rs10050860+rs27044+rs469876+rs17481856+rs13167972+rs17482078 | 38 | GT+CC+TT+NA+CC+AA+AA+AA+TT  TT+TT+CT+NA+CG+AG+AG+AG+CT | | 0.42 | 1.98×${10}^{-1}$ | 2 | TT+CT+CT+NA+GG+AG+AG+AG+CT  GT+TT+TT+NA+CG+AA+AA+AA+TT | | -0.81 | 0.016 | 0.025 |
|  | rs1065407+rs2287987+rs30187+rs10050860+rs27044+rs26653+rs469876+rs13167972+rs17482078 | 49 | GT+TT+CT+NA+CC+CC+AA+AA+TT  TT+CT+TT+NA+GG+GG+AG+AG+CT | | 0.05 | 8.42×${10}^{-1}$ | 2 | TT+CT+TT+NA+CG+CG+AG+AG+CT  GT+TT+CT+NA+GG+GG+AA+AA+TT | | -0.81 | 0.016 | 0.024 |
|  | rs1065407+rs2287987+rs30187+rs10050860+rs27044+rs26653+rs469876+rs17481856+rs13167972 | 48 | GT+CC+CC+NA+CC+CC+AA+AA+AA  TT+TT+CT+NA+GG+CG+AG+AG+AG | | 0.10 | 7.28×${10}^{-1}$ | 2 | TT+CT+TT+NA+CG+GG+AG+AG+AG+  GT+TT+CT+NA+GG+CG+AA+AA+AA | | -0.80 | 0.016 | 0.071 |
|  | rs30187+rs10050860+rs27044+rs26653+rs27434+rs469876+rs17481856+rs13167972+rs17482078 | 31 | TT+CT+CC+CC+GG+AA+GG+GG+TT  CT+TT+GG+CG+AG+AG+AA+AA+CT | | 0.43 | 2.13×${10}^{-1}$ | 2 | CT+TT+GG+CG+AA+AG+AG+AG+CT  TT+CT+CG+GG+AG+AA+AA+AA+TT | | -0.97 | 0.016 | 0.002 |
|  | rs1065407+rs30187+rs10050860+rs27044+rs26653+rs469876+rs17481856+rs13167972+rs17482078 | 47 | GT+CC+CT+CC+CC+AA+AA+AA+TT  TT+CT+TT+GG+CG+AG+AG+AG+CT | | 0.26 | 3.48×${10}^{-1}$ | 2 | TT+TT+TT+ CG+GG+AG+AG+AG+ CT  GT+CT+CT+GG+CG+AA+AA+AA+TT | | -0.96 | 0.017 | 0.002 |
|  | rs1065407+rs2287987+rs30187+rs10050860+rs27044+rs27434+rs469876+rs28096+rs17482078 | 36 | GT+CC+CT+NA+CC+AA+AA+GG+TT  TT+TT+TT+NA+GG+AG+AG+AG+CT | | -0.77 | 1.73×${10}^{-2}$ | 0 | NA | | NA | NA | 0.008 |
|  | rs1065407+rs10050860+rs27044+rs26653+rs27434+rs469876+rs17481856+rs28096+rs17482078 | 50 | GG+CT+CC+CC+GG+AA+AA+GG+TT  GT+TT+GG+CG+AG+AG+AG+AG+CT | | -0.08 | 7.62×${10}^{-1}$ | 2 | TT+TT+CG+GG+AA+AG+AG+AA+CT  GT+CT+GG+CG+AG+AA+AA+AG+TT | | -0.99 | 0.019 | 0.002 |
|  | rs1065407+rs27044+rs26653+rs27434+rs469876+rs17481856+rs28096+rs13167972+rs17482078 | 80 | GG+CC+CC+GG+AA+AA+GG+GG+TT  GT+GG+CG+AG+AG+AG+AG+AA+CT | | -0.02 | 9.24×${10}^{-1}$ | 2 | TT+CG+GG+AA+AG+AG+AA+AG+CT  GT+GG+CG+AG+AA+AA+AG+AA+TT | | -0.99 | 0.020 | 0.150 |
|  | rs1065407+rs10050860+rs26653+rs27434+rs469876+rs17481856+rs28096+rs13167972+rs17482078 | 69 | GG+CT+CC+GG+AA+AA+GG+GG+TT  GT+TT+CG+AG+AG+AG+AG+AA+CT | | 0.03 | 9.06×${10}^{-1}$ | 2 | TT+TT+GG+AA+AG+AG+AA+AA+CT  GT+CT+CG+AG+AA+AA+AG+AG+TT | | -0.99 | 0.020 | 0.002 |
|  | rs1065407+rs10050860+rs27044+rs26653+rs27434+rs469876+rs28096+rs13167972+rs17482078 | 79 | GG+CT+GG+CC+GG+AA+GG+GG+TT  GT+TT+CG+CG+AG+AG+AG+AA+CT | | 0.00 | 9.88×${10}^{-1}$ | 2 | TT+TT+CG+GG+AA+AG+AA+AG+CT  GT+CT+GG+CG+AG+AA+AG+AA+TT | | -0.99 | 0.020 | 0.002 |
|  | rs1065407+rs30187+rs27044+rs26653+rs27434+rs469876+rs17481856+rs28096+rs17482078 | 49 | GG+TT+GG+CC+GG+AA+GG+GG+TT  GT+CT+CG+CG+AG+AG+AA+AG+CT | | -0.24 | 3.88×${10}^{-1}$ | 2 | TT+CT+CG+GG+AA+AG+AG+AA+CT  GT+TT+GG+CG+AG+AA+AA+AG+TT | | -0.99 | 0.020 | 0.077 |
|  | rs1065407+rs30187+rs10050860+rs27044+rs26653+rs27434+rs469876+rs28096+rs17482078 | 50 | GG+CT+CT+GG+CC+GG+GG+GG+TT  GT+TT+TT+CG+CG+AG+AA+AG+CT | | -0.27 | 3.22×${10}^{-1}$ | 2 | TT+TT+TT+ CG+GG+AA+ AG+AA+CT  GT+CT+CT+GG+CG+AG+AA+AG+TT | | -0.99 | 0.020 | 0.002 |
|  | rs1065407+rs2287987+rs30187+rs10050860+rs27044+rs469876+rs17481856+rs28096+rs17482078 | 35 | GT+CC+CC+NA+CC+AA+AA+GG+TT  TT+TT+CT+NA+GG+AG+AG+AG+CT | | -0.74 | 2.31×${10}^{-2}$ | 0 | NA | | NA | NA | 0.022 |
|  | rs1065407+rs2287987+rs30187+rs27044+rs27434+rs469876+rs17481856+rs28096+rs17482078 | 35 | GT+TT+TT+CC+GG+AA+GG+GG+TT  TT+CT+CT+GG+AG+AG+AA+AG+CT | | -0.74 | 2.39×${10}^{-2}$ | 0 | NA | | NA | NA | 0.045 |
| #10 Interaction Effects | rs1065407+rs2287987+rs30187+rs10050860+rs27044+rs26653+rs27434+rs17481856+rs13167972+rs17482078 | 40 | GG+CC+TT+NA+CC+CC+GG+GG+AA+TT  GT+TT+CT+NA+GG+CG+AG+AA+AG+CT | | 0.28 | 3.57×${10}^{-1}$ | 2 | TT+CT+CT+NA+CG+GG+AA+AG+AG+CT  GT+TT+TT+NA+GG+CG+AG+AA+AA+TT | | -0.92 | 6.86×${10}^{-3}$ | 0.010 |
|  | rs1065407+rs2287987+rs30187+rs10050860+rs27044+rs26653+rs27434+rs469876+rs17481856+rs17482078 | 29 | 1)GG+CC+TT+NA+CC+CC+GG+GG+GG+TT  GT+TT+CT+NA+GG+CG+AG+AA+AA+CT | | 0.14 | 6.44×${10}^{-1}$ | 2 | TT+CT+CT+NA+CG+GG+AA+AG+AG+CT  GT+TT+TT+NA+GG+CG+AG+AA+AA+TT | | -0.89 | 9.35×${10}^{-3}$ | 0.017 |
|  | rs1065407+rs2287987+rs10050860+rs27044+rs26653+rs27434+rs469876+rs17481856+rs13167972+rs17482078 | 50 | GG+CC+NA+CC+CC+GG+AA+AA+AA+TT  GT+TT+NA+GG+CG+AG+AG+AG+AG+CT | | 0.02 | 9.48×${10}^{-1}$ | 2 | TT+CT+NA+CG+GG+AA+AG+AG+AG+CT  GT+TT+NA+GG+CG+AG+AA+AA+AA+TT | | -0.88 | 9.66×${10}^{-3}$ | 0.010 |
|  | rs1065407+rs2287987+rs30187+rs27044+rs26653+rs27434+rs469876+rs17481856+rs13167972+rs17482078 | 50 | GG+TT+TT+CC+CC+GG+AA+GG+AA+TT  GT+CT+CT+GG+CG+AG+AG+AA+AG+CT | | 0.10 | 7.26×${10}^{-1}$ | 2 | TT+CT+CT+CG+GG+AA+AG+AG+AG+CT  GT+TT+TT+GG+CG+AG+AA+AA+AA+TT | | -0.88 | 9.97×${10}^{-3}$ | 0.028 |
|  | rs1065407+rs2287987+rs30187+rs10050860+rs26653+rs27434+rs469876+rs17481856+rs13167972+rs17482078 | 40 | GG+CC +TT+NA+CC+GG +GG+GG+GG+TT  GT+TT+ CT+NA+ CG+AG+ AA+AA+ AA+CT | | -0.12 | 6.90×${10}^{-1}$ | 2 | TT+CT+ CT+NA +GG+AA+ AG+AG+ AG+CT  GT+TT +TT+NA +CG+AG+AA+AA +AA +TT | | -0.88 | 9.97×${10}^{-3}$ | 0.013 |
|  | rs1065407+rs2287987+rs30187+rs10050860+rs27044+rs27434+rs469876+rs17481856+rs13167972+rs17482078 | 40 | GT+CC+ TT+NA+ CC+GG+ AA+GG +AA+TT  TT+TT+ CT+NA +GG+AG+ AG+AA+ AG+CT | | 0.41 | 2.00×${10}^{-1}$ | 2 | TT+CT+ CT+NA+ CG+AA+ AG+AG +AG+CT  GT+TT+ TT+NA+ GG+AG+ AA+AA+ AA+TT | | -0.88 | 9.97×${10}^{-3}$ | 0.020 |
|  | rs1065407+rs2287987+rs30187+rs10050860+rs27044+rs26653+rs27434+rs469876+rs13167972+rs17482078 | 51 | GG+CC+ TT+NA+ CC+CC+ GG+AA+ AA+TT  GT+TT+ CT+NA+ GG+CG+ AG+AG+ AG+CT | | 0.06 | 8.28×${10}^{-1}$ | 2 | TT+CT+ CT+NA+ CG+GG +AA+AG+AG+CT  GT+TT +TT+NA +GG+CG+AG+AA+  AA+TT | | -0.88 | 9.97×${10}^{-3}$ | 0.009 |
|  | rs1065407+rs2287987+rs30187+rs10050860+rs27044+rs26653+rs27434+rs469876+rs17481856+rs13167972 | 49 | GG+CC+ TT+NA+ CC+CC+ GG+AA+  GG+AA  GT+TT+ CT+NA+ GG+CG+ AG+AG+ AA+AG | | 0.06 | 8.20×${10}^{-1}$ | 2 | TT+CT+ CT+ NA+ CG+GG+ AA+AG+ AG+AG  GT+TT+ TT+NA+ GG+CG+ AG+AA+ AA+AA | | -0.88 | 1.03×${10}^{-2}$ | 0.047 |
|  | rs1065407+rs2287987+rs30187+rs10050860+rs26653+rs27434+rs17481856+rs28096+rs13167972+rs17482078 | 50 | GG+CC+ TT+NA+ CC+GG+ GG+GG+ GG+TT  GT+TT+ CT+NA+ CG+AG+ AA+AG+ AA+CT | | 0.05 | 8.45×${10}^{-1}$ | 2 | TT+CT+ CT+NA+ GG+AA+ AG+AA+ AG+CT  GT+TT+ TT+NA +CG+AG+AA+AG +AA+TT | | -0.85 | 1.35×${10}^{-2}$ | 0.014 |
|  | rs1065407+rs2287987+rs30187+rs10050860+rs27044+rs26653+rs469876+rs17481856+rs13167972+rs17482078 | 50 | GT+CC+ CC+NA+ CC+CC+ AA+AA+ AA+TT  TT+TT+ CT+NA+ GG+CG+ AG+AG +AG+CT | | 0.09 | 7.37×${10}^{-1}$ | 2 | TT+CT+ TT+NA+ CG+GG+ AG+AG+ AG+ CT  GT+TT +CT+NA +GG+CG+AA+AA+ AA +TT | | -0.81 | 1.56×${10}^{-2}$ | 0.023 |
|  | rs1065407+rs2287987+rs30187+rs10050860+rs27044+rs27434+rs469876+rs17481856+rs28096+rs17482078 | 36 | GT+CC+ TT+NA+ CC+GG+ AG+GG+ GG+TT  TT+TT +CT+NA  +GG+AG+ AG+AA+ AG+CT | | -0.77 | 1.73×${10}^{-2}$ | 0 | NA | | NA | NA | 0.010 |
|  | rs1065407+rs10050860+rs27044+rs26653+rs27434+rs469876+rs17481856+rs28096+rs13167972+rs17482078 | 81 | GG+CT+CC+CC+GG+AA+AA+GG+GGTT  GT+TT+GG+CG+AG+AG+AG+AG+AA+CT | | -0.05 | 8.38×${10}^{-1}$ | 2 | TT+TT+CG+GG+AA+AG+AG+AA+AG+CT  GT+CT+GG+CG+AG+AA+AA+AG+AA+  TT | | -0.99 | 2.00×${10}^{-2}$ | 0.002 |
|  | rs1065407+rs30187+rs10050860+rs27044+rs26653+rs27434+rs469876+rs17481856+rs28096+rs17482078 | 50 | GG+TT+CT+GG+CC+GG+AA+GG+GG+TT  GT+CT+TT+CG+CG+AG+AG+AA+AA+CT | | -0.27 | 3.22×${10}^{-1}$ | 2 | TT+CT+TT+CG+GG+AA+AG+AG+AA+CT  GT+TT+CT+GG+CG+AG+AA+AA+AG+TT | | -0.99 | 2.00×${10}^{-2}$ | 0.002 |
|  | rs1065407+rs2287987+rs30187+rs10050860+rs27044+rs26653+rs27434+rs17481856+rs13167972+rs17482078 | 40 | GG+CC+TT+NA+CC+CC+GG+GG+AA+TT  GT+TT+CT+NA+GG+CG+AG+AA+AG+CT | | 0.28 | 3.57×${10}^{-1}$ | 2 | TT+CT+CT+NA+CG+GG+AA+AG+AG+CT  GT+TT+TT+NA+GG+CG+AG+AA+AA+TT | | -0.92 | 6.86×${10}^{-3}$ | 0.010 |
| #11Interaction Effects |  |  |  | |  |  |  |  | |  |  |  |
|  | rs1065407+rs2287987+rs30187+rs10050860+rs27044+rs26653+rs27434+rs469876+rs17481856+rs13167972+rs17482078 | 51 | 1)GG+CC+ TT+NA+ CC+CC+ GG+AA+ GG+AA+ TT  2)GT+TT+ CT+NA+ GG+CG+ AG+AG+ AA  +AG+CT | | 0.06 | 8.28×${10}^{-1}$ | 2 | 1)TT+CT+ CT+NA+ CG+GG +AA+ AG+AG+ AG+CT  2)GT+TT +TT+NA +GG+CG+ AG+AA+ AA+AA+ TT | | -0.88 | 9.97×${10}^{-3}$ | 0.018 |
