# Supplementary material for: ERAP1 polymorphisms interactions and their association with Behçet’s disease susceptibly: Application of Model-Based Multifactor Dimension Reduction Algorithm (MB-MDR)
Source: PLoS One. 2020 Feb 5;15(2):e0227997. doi: 10.1371/journal.pone.0227997 (PMC7001967; doi:10.1371/journal.pone.0227997)
Supplement: Attachment — Submitted filename: Response to reviewers.doc [file pone.0227997.s003.doc]

**Manuscript ID: PONE-D-19-28263 entitled “ERAP1 polymorphisms interactions and their association with Behçet’s disease susceptibly: Application of Model-Based Multifactor Dimension Reduction Algorithm (MB-MDR)”**

**Dear academic editor,**

**Zezhi Li,**

We are grateful to the reviewers and the editorial board of *PLOS ONE*, for their constructive criticisms on our paper. We revised the manuscript accordingly. The modifications are given as point-by-point responses to the comments of the reviewers. All changes in the manuscript are highlighted in yellow color. Also, all authors declare that they are agreed on the revision. We wish to thank the comments and hope that the revised version of the manuscript may be now suitable for publication.

Yours sincerely,

**Anoshirvan Kazemnejad**: Professor of Biostatistics, Department of Biostatistics, Faculty of Medical Sciences, Tarbiat Modares University, P.O.Box: 14115-111, Tehran, IR, Iran. E-mail address: kazem_an@modares.ac.ir

**Mahdi Mahmoudi:** Rheumatology Research Center, Tehran University of Medical Sciences, P.O. Box: 14117-13137, Tehran, IR. E-mail address: mahmoudim@tums.ac.ir

**Comments to the Author**

**Review Comments to the Author**

Please use the space provided to explain your answers to the questions above. You may also include additional comments for the author, including concerns about dual publication, research ethics, or publication ethics. (Please upload your review as an attachment if it exceeds 20,000 characters)

Reviewer #1: Behçet’s disease (BD) is a chronic multi-systemic vasculitis with a considerable prevalence in Asian countries. Riahi et al.’s manuscript investigates interactions of ERAP1 single nucleotide polymorphisms (SNPs) using a novel data mining method called Model-based multifactor dimensionality reduction (MB-MDR). They have included 748 BD patients and 776 healthy controls. Their results indicated that the TT genotype of rs1065407 had a synergistic effect on BD susceptibility, considering the significant main effect. In the second order of interactions, CC genotype of rs2287987 and GG genotype of rs1065407 had the most prominent synergistic effect (β=12.74). The mentioned genotypes also had significant interactions with CC genotype of rs26653 and TT genotype of rs30187 in the third-order (β=12.74 and β=12.73, respectively). In general, their results support their statement. However, there is still some issue that needs to be resolved.

1. It will be easy to understand the manuscript if the author explains how they define genotype.

**Response:** Thanks for your comment. We have added the explanation of genotypes to the method section.

2. It will be interesting if the author explains the difference between MB-MDR and other analysis methods?

**Response:** As mentioned at the end of the introduction section, conventional approaches (e.g., stepwise logistic regression) used for the analysis of genomic data are oversimplified and usually cannot consider all possible significant interactions between multiple polymorphisms. For example, logistics regression for identifying the most important of interactions between multiple polymorphisms is not an appropriate method in a large number of dichotomous variables (or SNPs), small sample size, and sparse data (1, 2). In logistic regression for high dimensional and sparse data, parameter estimation is a costly and non-accurate procedure that introduces large standard errors because sample sizes are too small compared to the order of interaction size. As a consequence, many false positives are generated when dealing with such data (3). In other words, logistic regression performs poorly when there is a dimensionality problem (4). With no ‘best’ statistical approach available, full combinatorial approaches (e.g., Multifactor Dimensionality Reduction) may be optimal for detecting SNPs interactions. MDR approach is now a reference in the epistasis and SNPs interactions detection field. No parameters are estimated (i.e., nonparametric), and no assumptions are made on the genetic model (i.e., model-free) under this supervised classification approach. However, MDR suffers from some major drawbacks, including that crucial interactions could be missed owing to pooling too many cells together or that proposed MDR analyses will only reveal at most one significant epistasis model, the selection being based on computationally demanding cross-validation and permutation strategies (5). To overcome the aforementioned hurdles, Model-Based MDR is a flexible framework to detect gene–gene or SNP-SNP interactions. Besides, MB-MDR has adequate power, even the presence of error sources or noise/ genotyping errors, missing genotypes, phenotypic mixtures, and genetic heterogeneity (5). The mentioned details were added in the introduction section.

3. I am wondering what is the advantage of MB-MDR compared with other methods when they analyze data?

**Response:** As mentioned in the response of the previous comment, Model-Based MDR is a flexible framework to detect gene–gene or SNP-SNP interactions with adequate power even the presence of error sources or noise/ genotyping errors, missing genotypes, phenotypic mixtures and genetic heterogeneity (5). Please see the before comment’s answer.

4. I am wondering if the author will get similar results (interactions of ERAP1 SNPs) by using other analysis methods?

**Response:** Based on the previous comment’s answer, logistic regression has a high computational burden and non-accurate estimations with large standard errors. However, the results of MDR were reported as follow:

Figure: Entropy-based network of interactions between12 ERAP1 SNPs.

For example, the results of entropy in MDR:

| Attribute | H(A) | H(A\|C) | I(A; C) |  |
| --- | --- | --- | --- | --- |
| --------- | ---- | ------ | ------ |  |
| Y | 1 | 1 | 0 |  |
| rs1065407 | 1.4731 | 1.4196 | 0.0534 |  |
| rs2287987 | 0.9805 | 0.8194 | 0.1611 |  |
| rs30187 | 1.518 | 1.4962 | 0.0218 |  |
| rs10050860 | 0.8635 | 0.6771 | 0.1864 |  |
| rs27044 | 1.2802 | 1.2662 | 0.0139 |  |
| rs26653 | 1.486 | 1.4505 | 0.0354 |  |
| rs27434 | 1.425 | 1.4107 | 0.0142 |  |
| rs469876 | 1.1953 | 1.1863 | 0.009 |  |
| rs17481856 | 0.7089 | 0.7075 | 0.0014 |  |
| rs28096 | 1.4858 | 1.4455 | 0.0402 |  |
| rs13167972 | 1.5345 | 1.5059 | 0.0285 |  |

| Attribute A | Attribute B | H(AB) | H(AB\|C) | I(A;B) | IG(A;B;C) | I(AB;C) |  |
| --- | --- | --- | --- | --- | --- | --- | --- |
| ----------- | ----------- | ----- | ------- | ------ | -------- | ------- |  |
| rs1065407 | Y | 0.9889 | 0.9489 | 1.4842 | -0.0136 | 0.0399 |  |
| rs2287987 | Y | 0.7689 | 0.606 | 1.2116 | 0.0018 | 0.1629 |  |
| rs2287987 | rs1065407 | 0.7778 | 0.61 | 1.6757 | -0.0467 | 0.1678 |  |
| rs30187 | Y | 0.9376 | 0.9314 | 1.5804 | -0.0157 | 0.0062 |  |
| rs30187 | rs1065407 | 1 | 0.9377 | 1.9911 | -0.0131 | 0.0622 |  |
| rs30187 | rs2287987 | 0.7807 | 0.6112 | 1.7178 | -0.0134 | 0.1695 |  |
| rs10050860 | Y | 0.7659 | 0.5795 | 1.0976 | 0 | 0.1864 |  |
| rs10050860 | rs1065407 | 0.7659 | 0.5795 | 1.5706 | -0.0534 | 0.1864 |  |
| rs10050860 | rs2287987 | 0.7748 | 0.6087 | 1.0691 | -0.1813 | 0.1662 |  |
| rs10050860 | rs30187 | 0.7659 | 0.5795 | 1.6156 | -0.0218 | 0.1864 |  |
| rs27044 | Y | 0.9936 | 0.9899 | 1.2865 | -0.0103 | 0.0037 |  |
| rs27044 | rs1065407 | 0.9906 | 0.9463 | 1.7626 | -0.0231 | 0.0443 |  |
| rs27044 | rs2287987 | 0.7893 | 0.6335 | 1.4713 | -0.0192 | 0.1558 |  |
| rs27044 | rs30187 | 0.9361 | 0.9293 | 1.8621 | -0.029 | 0.0068 |  |
| rs27044 | rs10050860 | 0.7659 | 0.5795 | 1.3777 | -0.0139 | 0.1864 |  |
| rs26653 | Y | 0.9103 | 0.8887 | 1.5757 | -0.0139 | 0.0216 |  |
| rs26653 | rs1065407 | 1 | 0.9277 | 1.959 | -0.0165 | 0.0723 |  |
| rs26653 | rs2287987 | 0.7893 | 0.6335 | 1.6771 | -0.0407 | 0.1558 |  |
| rs26653 | rs30187 | 0.9856 | 0.9697 | 2.0184 | -0.0414 | 0.0159 |  |
| rs26653 | rs10050860 | 0.7659 | 0.5795 | 1.5835 | -0.0354 | 0.1864 |  |
| rs26653 | rs27044 | 0.9863 | 0.962 | 1.7799 | -0.0251 | 0.0243 |  |
| rs27434 | Y | 0.9986 | 0.9914 | 1.4263 | -0.0071 | 0.0072 |  |
| rs27434 | rs1065407 | 0.9996 | 0.9406 | 1.8984 | -0.0087 | 0.059 |  |
| rs27434 | rs2287987 | 0.7921 | 0.643 | 1.6133 | -0.0262 | 0.1491 |  |
| rs27434 | rs30187 | 0.9974 | 0.9891 | 1.9456 | -0.0278 | 0.0083 |  |
| rs27434 | rs10050860 | 0.7659 | 0.5795 | 1.5225 | -0.0142 | 0.1864 |  |
| rs27434 | rs27044 | 0.9974 | 0.9891 | 1.7078 | -0.0199 | 0.0083 |  |
| rs27434 | rs26653 | 0.9961 | 0.9849 | 1.9149 | -0.0385 | 0.0112 |  |
| rs469876 | Y | 0.9551 | 0.9505 | 1.2402 | -0.0045 | 0.0045 |  |
| rs469876 | rs1065407 | 0.9964 | 0.9549 | 1.6719 | -0.0209 | 0.0415 |  |
| rs469876 | rs2287987 | 0.7807 | 0.6297 | 1.395 | -0.0191 | 0.151 |  |
| rs469876 | rs30187 | 0.9753 | 0.9324 | 1.738 | 0.012 | 0.0429 |  |
| rs469876 | rs10050860 | 0.7659 | 0.5795 | 1.2929 | -0.009 | 0.1864 |  |
| rs469876 | rs27044 | 0.8712 | 0.8636 | 1.6042 | -0.0153 | 0.0076 |  |
| rs469876 | rs26653 | 0.9796 | 0.9206 | 1.7017 | 0.0145 | 0.059 |  |
| rs469876 | rs27434 | 1 | 0.9813 | 1.6203 | -0.0046 | 0.0186 |  |
| rs17481856 | Y | 0.9812 | 0.9812 | 0.7277 | -0.0014 | 0 |  |
| rs17481856 | rs1065407 | 0.9889 | 0.9489 | 1.1931 | -0.0149 | 0.0399 |  |
| rs17481856 | rs2287987 | 0.7807 | 0.6379 | 0.9087 | -0.0196 | 0.1428 |  |
| rs17481856 | rs30187 | 0.9974 | 0.9901 | 1.2296 | -0.0159 | 0.0073 |  |
| rs17481856 | rs10050860 | 0.7659 | 0.5795 | 0.8065 | -0.0014 | 0.1864 |  |
| rs17481856 | rs27044 | 0.9936 | 0.9899 | 0.9955 | -0.0116 | 0.0037 |  |
| rs17481856 | rs26653 | 0.8441 | 0.8053 | 1.3509 | 0.002 | 0.0388 |  |
| rs17481856 | rs27434 | 0.9977 | 0.99 | 1.1362 | -0.008 | 0.0077 |  |
| rs17481856 | rs469876 | 0.9999 | 0.9919 | 0.9043 | -0.0023 | 0.0081 |  |
| rs28096 | Y | 0.9643 | 0.9297 | 1.5214 | -0.0056 | 0.0346 |  |
| rs28096 | rs1065407 | 0.9993 | 0.9376 | 1.9595 | -0.0319 | 0.0617 |  |
| rs28096 | rs2287987 | 0.7807 | 0.6209 | 1.6855 | -0.0415 | 0.1598 |  |
| rs28096 | rs30187 | 0.9725 | 0.929 | 2.0313 | -0.0186 | 0.0435 |  |
| rs28096 | rs10050860 | 0.7659 | 0.5795 | 1.5833 | -0.0402 | 0.1864 |  |
| rs28096 | rs27044 | 0.9665 | 0.9292 | 1.7995 | -0.0168 | 0.0373 |  |
| rs28096 | rs26653 | 0.9949 | 0.9359 | 1.9768 | -0.0166 | 0.059 |  |
| rs28096 | rs27434 | 0.9706 | 0.9324 | 1.9402 | -0.0163 | 0.0382 |  |
| rs28096 | rs469876 | 0.9842 | 0.9494 | 1.6968 | -0.0144 | 0.0348 |  |
| rs28096 | rs17481856 | 0.9643 | 0.9297 | 1.2304 | -0.007 | 0.0346 |  |
| rs13167972 | Y | 0.8817 | 0.8593 | 1.6528 | -0.0062 | 0.0224 |  |
| rs13167972 | rs1065407 | 0.9906 | 0.9487 | 2.0169 | -0.0401 | 0.0418 |  |
| rs13167972 | rs2287987 | 0.7865 | 0.6323 | 1.7285 | -0.0354 | 0.1542 |  |
| rs13167972 | rs30187 | 0.9771 | 0.9337 | 2.0754 | -0.007 | 0.0434 |  |
| rs13167972 | rs10050860 | 0.7659 | 0.5795 | 1.632 | -0.0285 | 0.1864 |  |
| rs13167972 | rs27044 | 0.8993 | 0.8746 | 1.9153 | -0.0177 | 0.0247 |  |
| rs13167972 | rs26653 | 0.9155 | 0.8517 | 2.105 | -0.0002 | 0.0638 |  |
| rs13167972 | rs27434 | 0.9779 | 0.9376 | 1.9815 | -0.0025 | 0.0403 |  |
| rs13167972 | rs469876 | 0.9138 | 0.8916 | 1.816 | -0.0154 | 0.0221 |  |
| rs13167972 | rs17481856 | 0.8712 | 0.8496 | 1.3722 | -0.0083 | 0.0216 |  |
| rs13167972 | rs28096 | 0.9945 | 0.9455 | 2.0257 | -0.0197 | 0.049 |  |

Reviewer #2: Comments for “ERAP1 polymorphisms interactions and their association with Behçet’s disease susceptibly: Application of Model-Based Multifactor Dimension Reduction Algorithm (MB-MDR)”

In this study, Parisa et al. evaluated the potential synergistic and antagonism effect of ERAP1 SNPs on patients with Behçet’s disease (BD) by using a new method MB-MDR. The analysis results are based on a considerable number of cases (748) and healthy controls (776), which could comprehensively assess the correlation between ERAP1 SNPs and disease occurrence in the area surveyed; thus, it supplies a way to predict the risk of disease. While the mechanism of Behçet’s disease is uncertain and multiple factors could contribute to this disease, animal models for this disease is unavailable at present, acquisition of the statistics from a clinical sample is the only way we approach this disease. Therefore, our analysis methods largely determine the reliability of the statistic. This study provides the data calculated by the “mbmdr” R package version 3.5.1, the result could be insufficiently supported due to the following three major concerns.

Response: Thanks for your comments. The mentioned corrections were done.

1. Although this method MB-MDR is powerful, it doesn’t mean its results are more reliable than other methods. Because the data are confronted with other research data, for example, conclusions from Kirino et al. 2013 and your former conclusion in Mahmoudi et al. 2018.

Response: In general, MB-MDR has different results compared to other algorithms in the mentioned studies because of the different model’s assumptions and methodology. However, typical approaches perform poorly when there is a dimensionality problem for identifying interactions in genetics studies. On the other hand, MDR reduces dimensions by converting a high-dimensional model to a one-dimensional one (4). Nevertheless, MDR as a non-parametric algorithm suffers from some major drawbacks, including that critical interactions could be missed owing to pooling too many cells together or that proposed MDR analyses will only reveal the most significant epistasis model based on computationally demanding cross-validation and permutation strategies (5). Parametric algorithm (MB-MDR) tend to have particular model assumptions, which lead to our ability to determine statistical significance under such assumptions. In a non-parametric test (MDR), we often have fewer assumptions to evaluate, but also differences in how statistical significance is determined. For example, if we run a non-parametric test, such as MDR, there is no P-value table. Based on the permutation test in cross-validation of both MDR, MB-MDR, both algorithms considered as appropriate and reference methods in the epistasis and SNPs interactions detection field (5).

2. Since DB is a chronic disease caused by many factors, evaluation of the synergism of SNPs in an individual gene could not be meaningful, while the synergism of SNPs between or among multiple genes could be more reliable.

Response: Thank you for your valuable comment. As you have mentioned, there are many factors contributing to BD’s pathophysiology, including various genes and also multiple environmental variables. Indeed, evaluating these factors, especially the gene-gene interactions, would shed more light on the underlying mechanisms of BD. However, this article is the starting point where we explored the application of a robust statistical method to investigate the interactions between ERAP1 SNPs in BD. Undoubtedly, our team is determined to test such powerful methods to evaluate the synergism of different genes’ SNPs in BD and other multifactor rheumatic diseases in future studies.

3. In this study, there are only tables, which are not direct-viewing diagrams, especially for the Table 2, if authors draw a diagram showing the main and interaction effects, it could be better for understanding by the readers.

Response: Thank you for the comment, the network based on the results of Table 2 and 3 were shown and added as Figure 1 in the results section.

Figure 1: SNP-SNP entropy-based interaction network of 12 ERAP1 SNPs.

Reviewer #3:

1. In the Introduction section, I would like the author of this paper to give a more thorough description of how ERAP1 gene polymorphisms are associated with HLA-B*51.

Response: Thank you for your comment. We have provided more explanation regarding the possible association of ERAP1 gene polymorphisms and HLA-B*51 in the introduction section.

2. In the Discussion section, the author should discuss the advantage of the MB-MDR method

Response: Thanks. The advantages of the MB-MDR method were added at the end of the discussion section.

1.	Briggs F, Ramsay P, Madden E, Norris J, Holers V, Mikuls TR, et al. Supervised machine learning and logistic regression identifies novel epistatic risk factors with PTPN22 for rheumatoid arthritis. Genes and immunity. 2010;11(3):199.

2.	Johnstone IM, Titterington DM. Statistical challenges of high-dimensional data. The Royal Society Publishing; 2009.

3.	Niel C, Sinoquet C, Dina C, Rocheleau G. A survey about methods dedicated to epistasis detection. Frontiers in genetics. 2015;6:285.

4.	Kim H, Jeong H-B, Jung H-Y, Park T, Park M. Multivariate Cluster-Based Multifactor Dimensionality Reduction to Identify Genetic Interactions for Multiple Quantitative Phenotypes. BioMed research international. 2019;2019.

5.	John JMM, Van Lishout F, Van Steen K. Model-Based Multifactor Dimensionality Reduction to detect epistasis for quantitative traits in the presence of error-free and noisy data. European Journal of Human Genetics. 2011;19(6):696.
